# Supplementary material for: Frequency and mechanisms of LINE-1 retrotransposon insertions at CRISPR/Cas9 sites
Source: Nat Commun. 2022 Jun 27;13:3685. doi: 10.1038/s41467-022-31322-3 (PMC9237045; doi:10.1038/s41467-022-31322-3)
Supplement: Supplementary file 1 — Supplementary Information [file 41467_2022_31322_MOESM1_ESM.pdf]

Supplementary Information

Frequency and mechanisms of LINE-1 retrotransposon insertions at CRISPR/Cas9 sites

Jianli Tao[1#,*], Qi Wang[1#], Carlos Mendez-Dorantes[2], Kathleen H. Burns[2], Roberto Chiarle[1,3*]

[1]Department of Pathology, Boston Children's Hospital and Harvard Medical School, Boston, MA 02115, USA

[2]Department of Oncologic Pathology, Dana-Farber Cancer Institute, Boston, MA 02115, USA

[3]Department of Molecular Biotechnology and Health Sciences, University of Torino, Torino, 10126 Italy

[#]These authors contributed equally

*email: roberto.chiarle@childrens.harvard.edu; jianli.tao@childrens.harvard.edu

The PDF file includes:

- Supplementary Figures 1-14
- Supplementary Tables 1-5
- Supplementary Sequences 1-13

Supplementary Figures

Supplementary Figure 1. CRISPR/Cas9 facilitates *de novo* L1-ORFeus insertions at the *MYC* locus in HEK293T cells.

Supplementary Figure 2. CRISPR/Cas9 facilitates *de novo* L1-ORFeus insertions at the *RAG1* locus in HEK293T cells.

Supplementary Figure 3. CRISPR/Cas9 facilitates *de novo* L1-ORFeus insertions at the *CCR5* locus in HEK293T cells.

Supplementary Figure 4. Development of PolyA-seq to capture genome-wide *de novo* L1-ORFeus insertion events.

Supplementary Figure 5. RT-dependent *de novo* L1-ORFeus insertions at CRISPR/Cas9 off-target sites in HEK293T cells.

Supplementary Figure 6. Detailed characterization of *de novo* L1-ORFeus insertions at CRISPR/Cas9 target site in HeLa cells.

Supplementary Figure 7. RT-dependent *de novo* L1-ORFeus insertions at CRISPR/Cas9 target site in U2OS cells.

Supplementary Figure 8. RT-dependent *de novo* L1RP insertions at CRISPR/Cas9 target site in HEK293T cells.

Supplementary Figure 9. Safety evaluation of prime editing at the *MYC* (+2-4AAA del) site in HEK293T cells.

Supplementary Figure 10. Safety evaluation of prime editing at the *MYC* (+5GtoC) or *FANCF* (+5GtoT) site in HEK293T cells.

Supplementary Figure 11. Characterization of PE3 (*MYC* +5GtoC) and PE3 (*FANCF* +5GtoT) editing in HEK293T cells.

Supplementary Figure 12. Characterization of *de novo* L1-ORFeus insertions in PE3 edited HEK293T cells.

Supplementary Figure 13. *De novo* L1-ORFeus insertions are rare in base editing in HEK293T cells.

Supplementary Figure 14. Endogenous L1 sequences found at CRISPR/Cas9 target sites in HEK293T cells.

Supplementary Figure 1

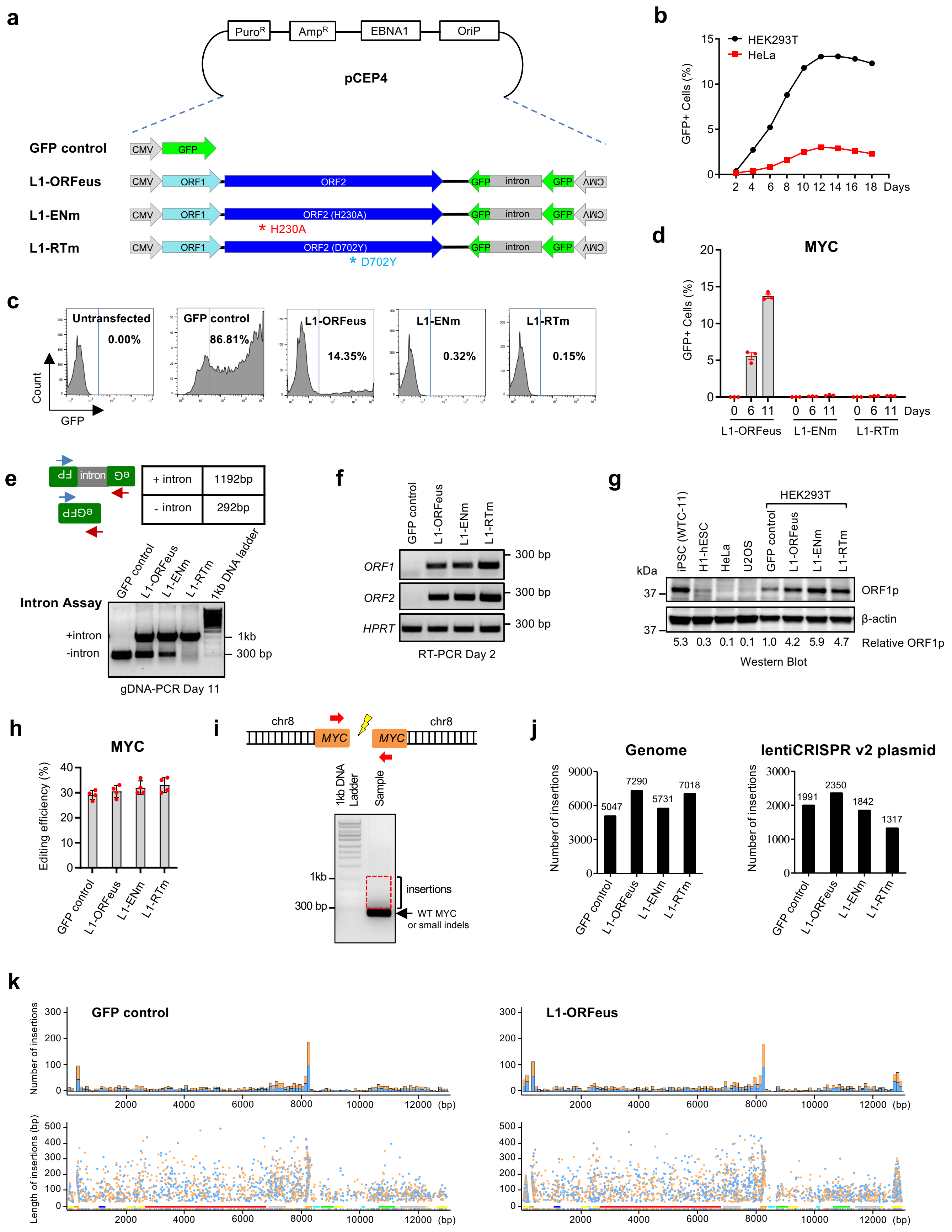

Supplementary Figure 1 to be continued

Supplementary Figure 1 Continued

k (continued)

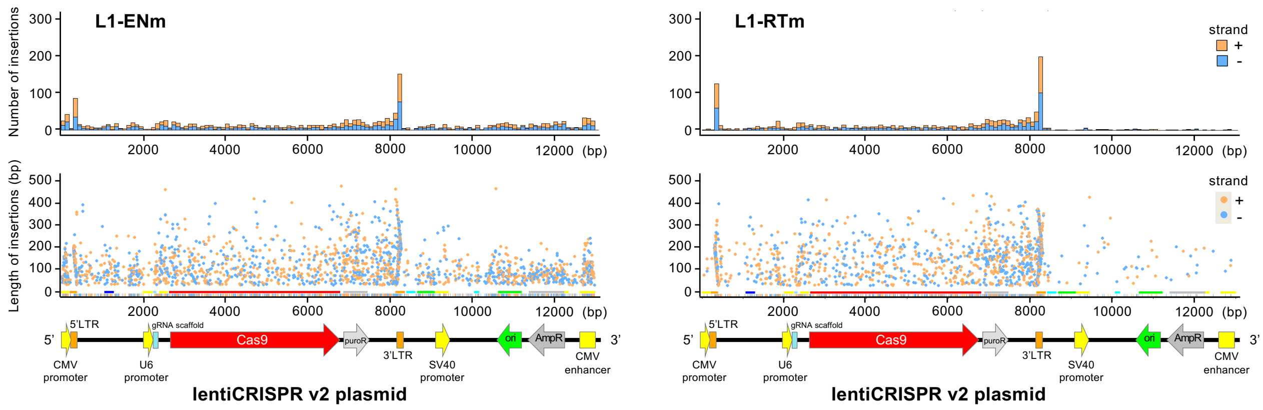

l

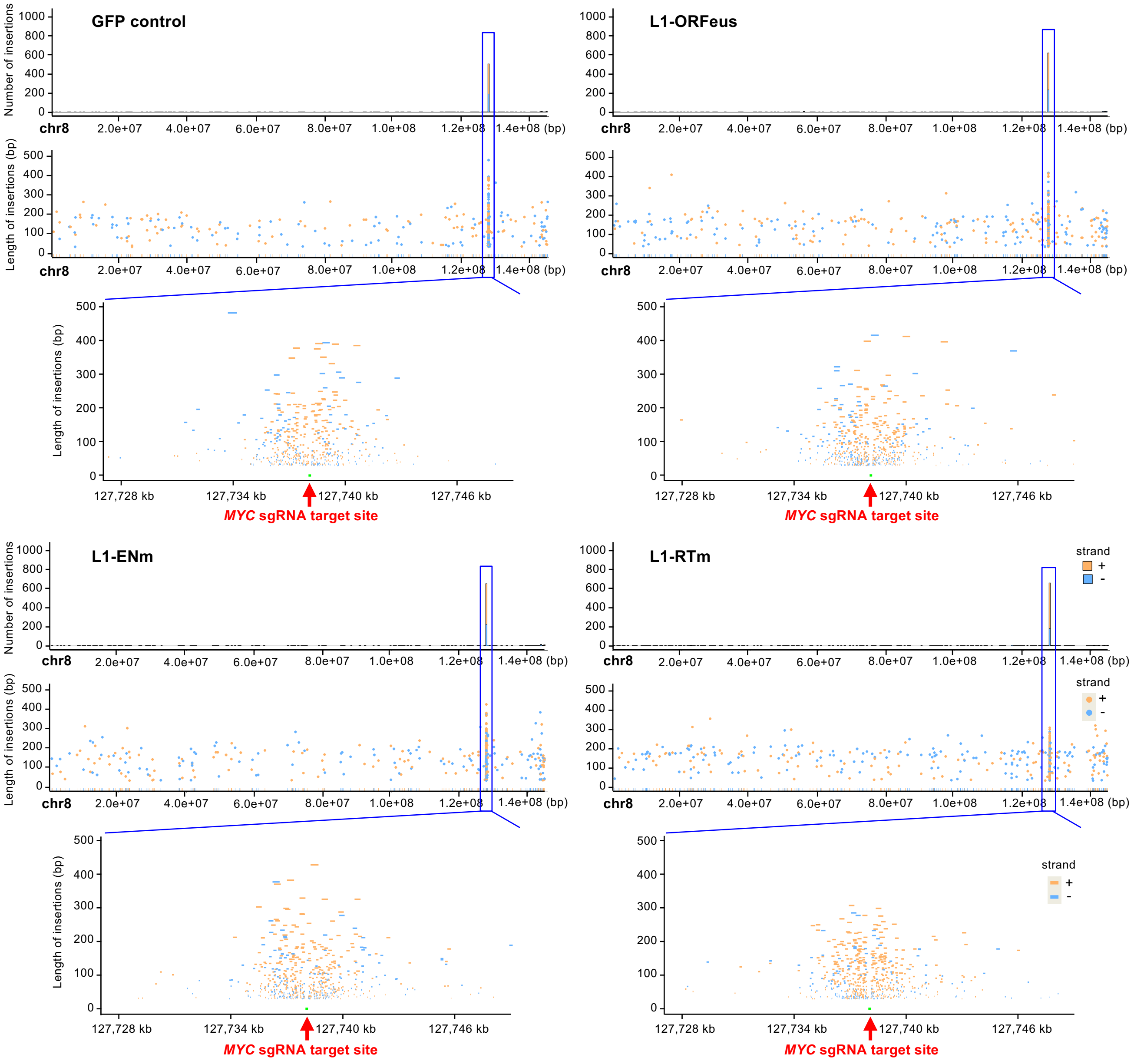

Supplementary Figure 1. CRISPR/Cas9 facilitates *de novo* L1-ORFeus insertions at the *MYC* locus in HEK293T cells. a, Schematic representation of pCEP4 GFP control, L1-ORFeus, L1-ENm and L1-RTm plasmids used in our study. CMV=CMV promoter; ORF=open reading frame; Intron=human gamma globin intron. **b**, Percentage of GFP-positive cells in HEK293T or HeLa cells at the indicated days after transfection of the L1-ORFeus reporter plasmid. Puromycin selection was performed 2 days post-transfection, and GFP-positive cells were analyzed by flow cytometry. **c-g**, Experiments were performed as shown in **Fig. 1b**, then GFP-positive HEK293T cells were analyzed at day 11 (**c**) or at day 6 or day 11 (**d**). Data are Mean±SD, n=3 independent experiments. The PCR assay across the intron of the GFP reporter gene (Intron assay) was performed at day 11. Splicing of the artificial intron from GFP results in a shorter PCR product (from 1192 bp to 292 bp) (**e**, bottom); blue and red arrows indicate the forward or reverse primer, respectively (**e**, top). RT-PCR was performed to analyze the RNA levels of ORF1 and ORF2 at day 2 (**f**). Western Blot was performed to detect L1 ORF1p protein (along with other indicated cell lines) (**g**); the relative expression of the ORF1p protein was measured by densitometry using Image J. **h**, Editing efficiency of CRISPR/Cas9 at the *MYC* target site in HEK293T cells. Data are Mean±SD, n=4 independent experiments. **i**, Top: Schematic design of improved amplicon sequencing at *MYC* locus. Bottom: to reduce background from uncut or small indels (i.e. the strong PCR band), we enrich the insertion events by gel excision between 300 bp to 1 kb. **j**, Numbers of insertions mapped to the human genome (**j**, left) or lentiCRISPR v2 plasmid (**j**, right) obtained by amplicon sequencing at the *MYC* locus targeted by *MYC* CRISPR/Cas9 in HEK293T cells expressing L1-ORFeus, L1-ENm, L1-RTm or GFP control. **k**,**l**, Numbers and fragment lengths of lentiCRISPR v2 plasmid insertions (**k**) or chromosome 8 insertions (**l**) obtained by amplicon sequencing at the *MYC* locus in HEK293T cells targeted by *MYC* CRISPR/Cas9 expressing L1-ORFeus, L1-ENm, L1-RTm or GFP control. Source data are provided as a Source Data file.

Supplementary Figure 2

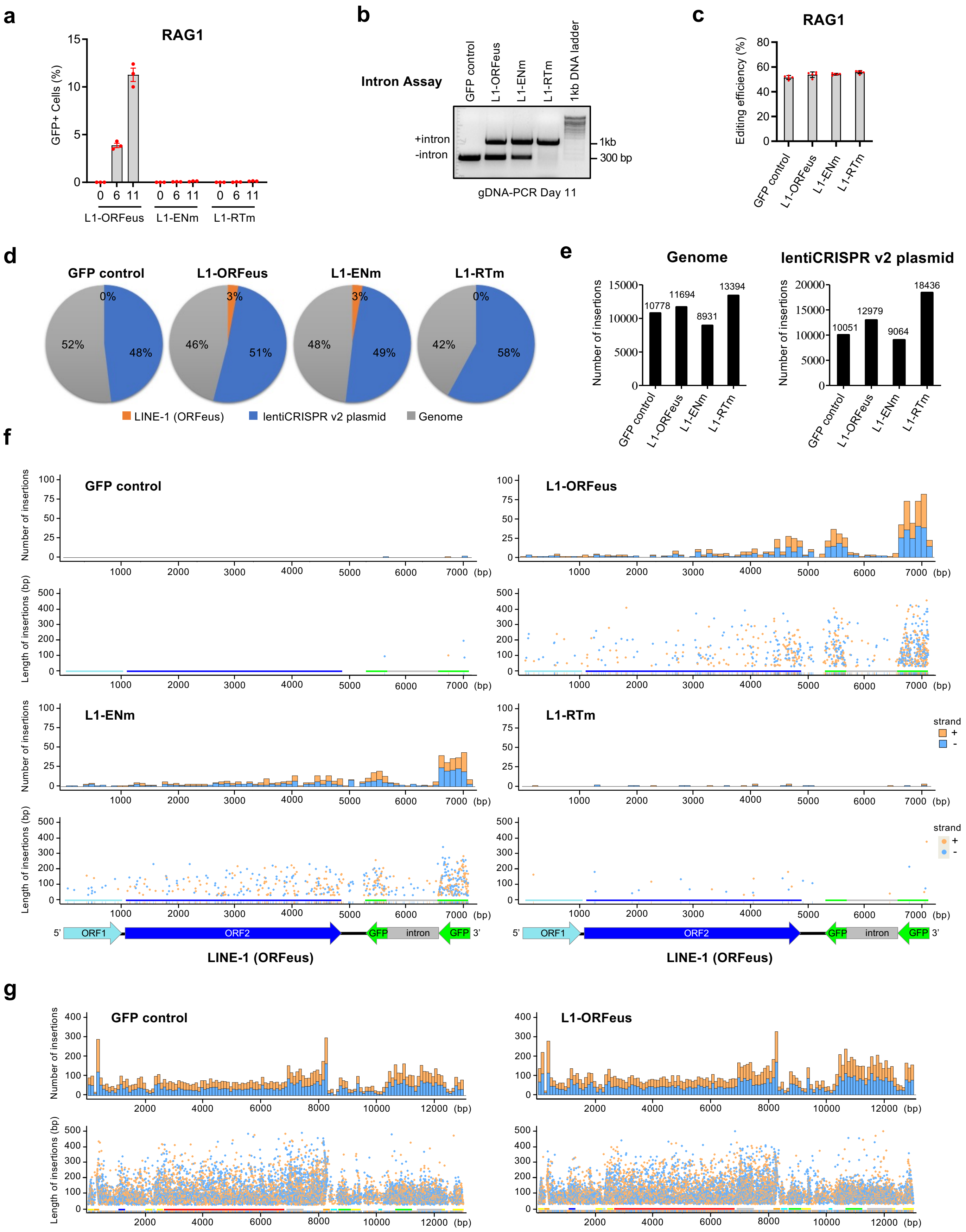

Supplementary Figure 2 to be continued

Supplementary Figure 2 Continued

g (continued)

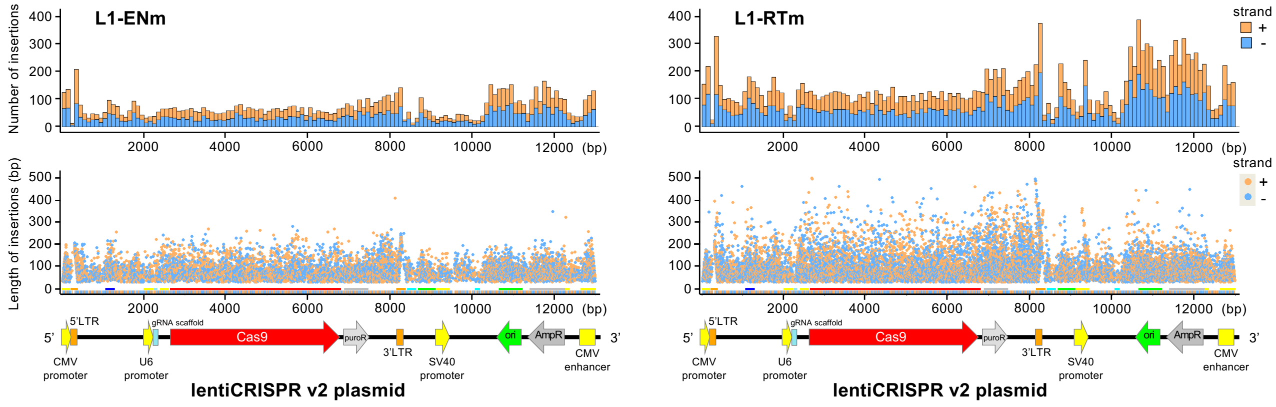

h

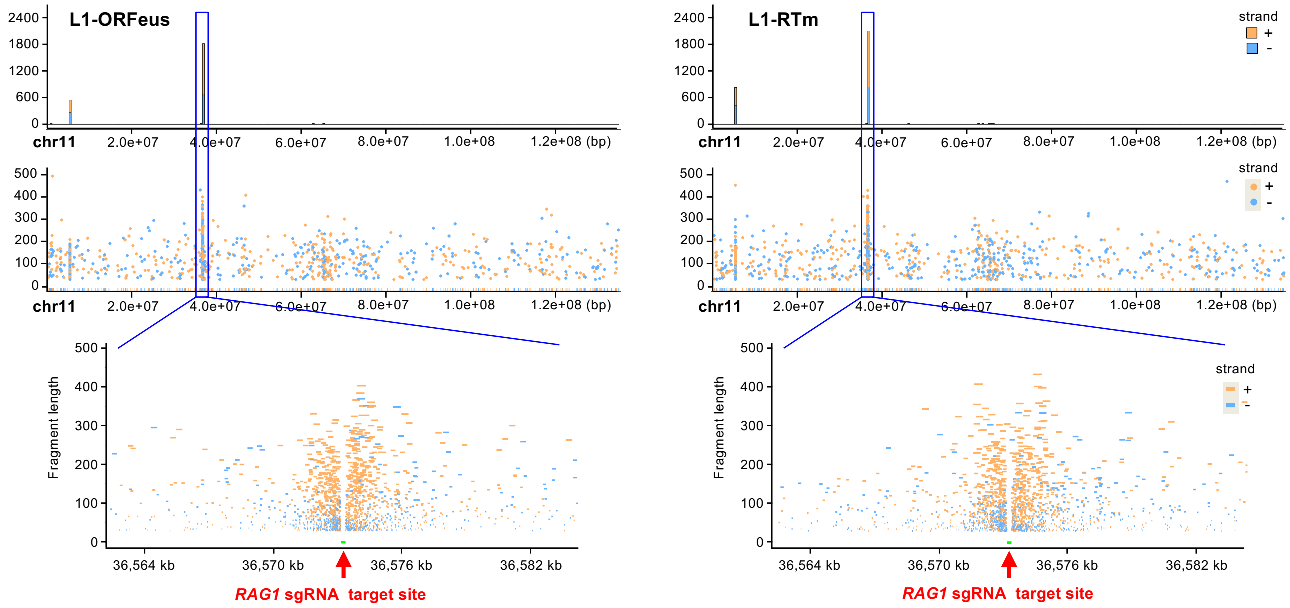

i

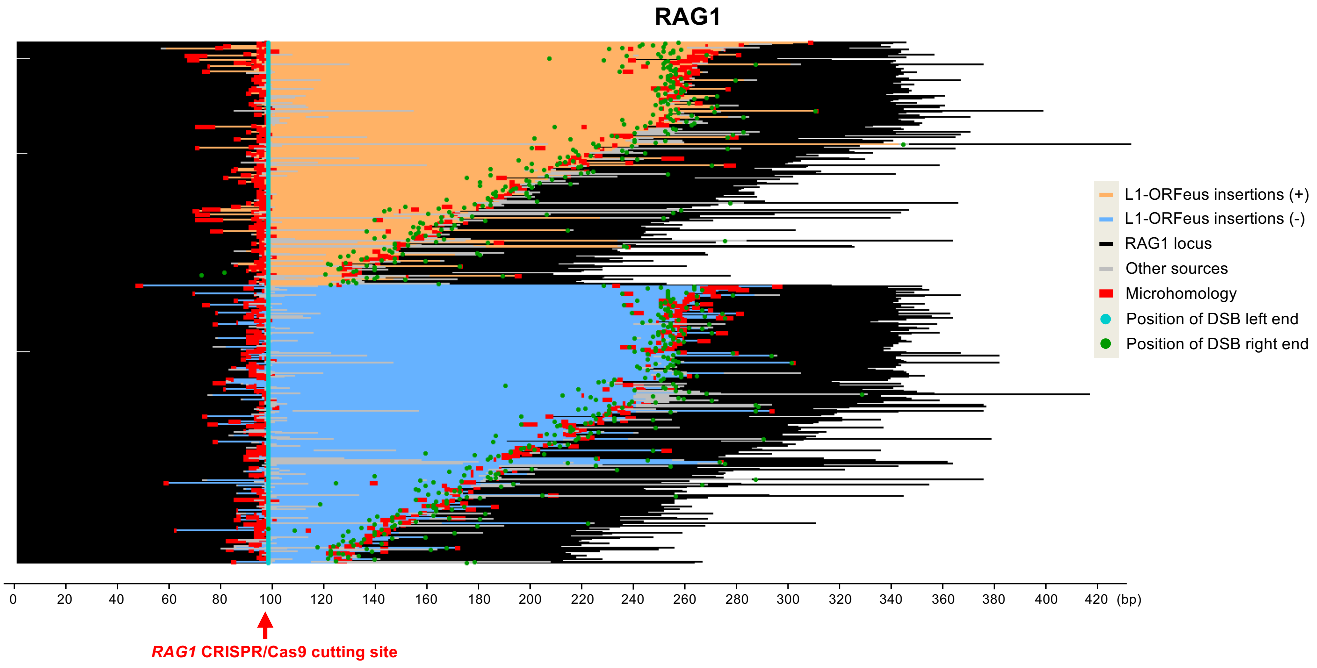

Supplementary Figure 2. CRISPR/Cas9 facilitates *de novo* L1-ORFeus insertions at the *RAG1* locus in HEK293T cells. **a**,**b**, Percentage of GFP-positive HEK293T cells at day 6 or day 11 after L1-ORFeus transfection and introducing CRISPR/Cas9-mediated DSBs at the *RAG1* locus (**a**) (Data are Mean±SD, n=3 independent experiments). Intron assay was performed to probe the L1-ORFeus retrotransposition events by genomic DNA-PCR at day 11 (**b**). **c**, Editing efficiency of CRISPR/Cas9 at the *RAG1* target site in HEK293T cells expressing L1-ORFeus, L1-ENm, L1-RTm or GFP control. Data are Mean±SD, n=5 independent experiments. **d**, Pie charts show the relative abundance of three main types of insertions (i.e., L1-ORFeus, lentiCRISPR v2 plasmid and genomic fragments) obtained by amplicon sequencing at the *RAG1* locus in HEK293T cells targeted by *RAG1* CRISPR/Cas9 expressing L1-ORFeus, L1-ENm, L1-RTm or GFP control. **e**, Numbers of insertions mapped to the human genome (**e**, left) or lentiCRISPR v2 plasmid (**e**, right) obtained by amplicon sequencing at the *RAG1* locus in HEK293T cells targeted by *RAG1* CRISPR/Cas9 expressing L1-ORFeus, L1-ENm, L1-RTm or GFP control. **f-h**, Numbers and fragment lengths of L1-ORFeus insertions (**f**), lentiCRISPR v2 plasmid insertions (**g**) or chromosome 11 insertions (**h**) obtained by amplicon sequencing at the *RAG1* locus in HEK293T cells targeted by *RAG1* CRISPR/Cas9 expressing L1-ORFeus, L1-ENm, L1-RTm or GFP control. Example L1-ORFeus insertions bridging the intron of GFP obtained by amplicon sequencing at the *RAG1* CRISPR/Cas9 target site in HEK293T cells expressing L1-ORFeus are shown in **Supplementary Sequence 7**. **i**, Similar to **Fig. 2b**, L1-ORFeus junction analysis obtained by amplicon sequencing at the *RAG1* locus targeted by *RAG1* CRISPR/Cas9 in HEK293T cells expressing L1-ORFeus. Source data are provided as a Source Data file.

Supplementary Figure 3

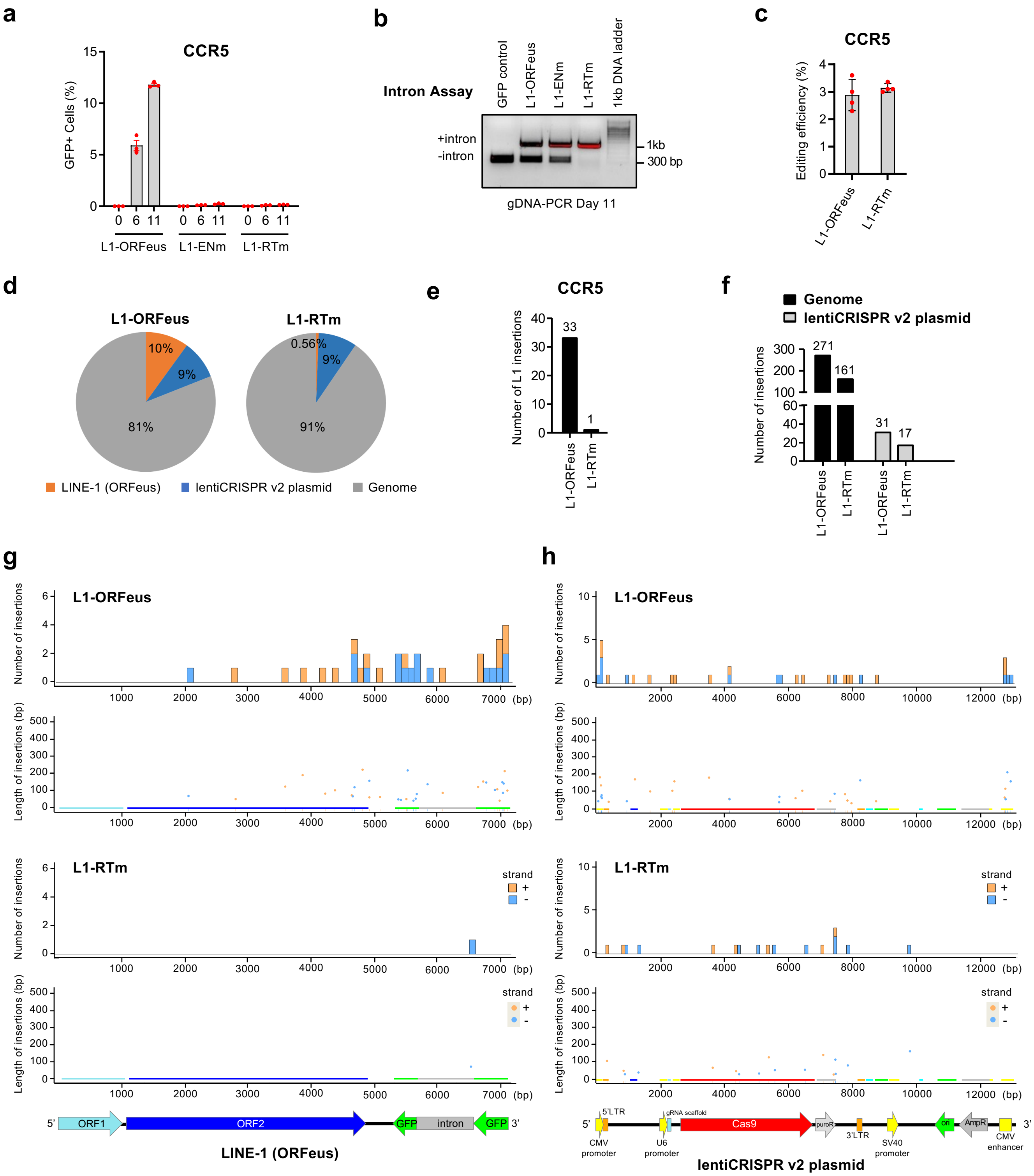

Supplementary Figure 3. CRISPR/Cas9 facilitates *de novo* L1-ORFeus insertions at the *CCR5* locus in HEK293T cells. a,**b**, Percentage of GFP-positive HEK293T cells at day 6 or day 11 after L1-ORFeus transfection and introducing CRISPR/Cas9-mediated DSBs at the *CCR5* locus (**a**) (Data are Mean±SD, n=3 independent experiments), Intron assay was performed by genomic DNA-PCR at day 11 (**b**). **c**, Editing efficiency of CRISPR/Cas9 at the *CCR5* target site in HEK293T cells expressing L1-ORFeus or L1-RTm. Data are Mean±SD, n=4 independent experiments. **d**, Pie charts show the relative abundance of three main types of insertions (i.e., L1-ORFeus, lentiCRISPR v2 plasmid and genomic fragments) obtained by amplicon sequencing at the *CCR5* locus in HEK293T cells targeted by *CCR5* CRISPR/Cas9 expressing L1-ORFeus or L1-RTm. **e**,**f**, Numbers of insertions mapped to LINE-1 (ORFeus) (**e**), the human genome (**f**, left) or lentiCRISPR v2 plasmid (**f**, right) obtained by amplicon sequencing at the *CCR5* locus in HEK293T cells targeted by *CCR5* CRISPR/Cas9 expressing L1-ORFeus or L1-RTm. **g**,**h**, Numbers and fragment lengths of L1-ORFeus insertions (**g**) or lentiCRISPR v2 plasmid insertions (**h**) obtained by amplicon sequencing at the *CCR5* locus in HEK293T cells targeted by *CCR5* CRISPR/Cas9 expressing L1-ORFeus or L1-RTm. Example L1-ORFeus insertion bridging the intron of GFP obtained by amplicon sequencing at the *CCR5* CRISPR/Cas9 target site in HEK293T cells expressing L1-ORFeus are shown in **Supplementary Sequence 8**. The only L1-ORFeus insertional sequence obtained by amplicon sequencing at the *CCR5* CRISPR/Cas9 target site in HEK293T cells expressing L1-RTm is shown in **Supplementary Sequence 10.** The orientation of insertions is shown in orange when the fragment is oriented 5' to 3' (+) or blue when 3' to 5' (-). Source data are provided as a Source Data file.

Supplementary Figure 4

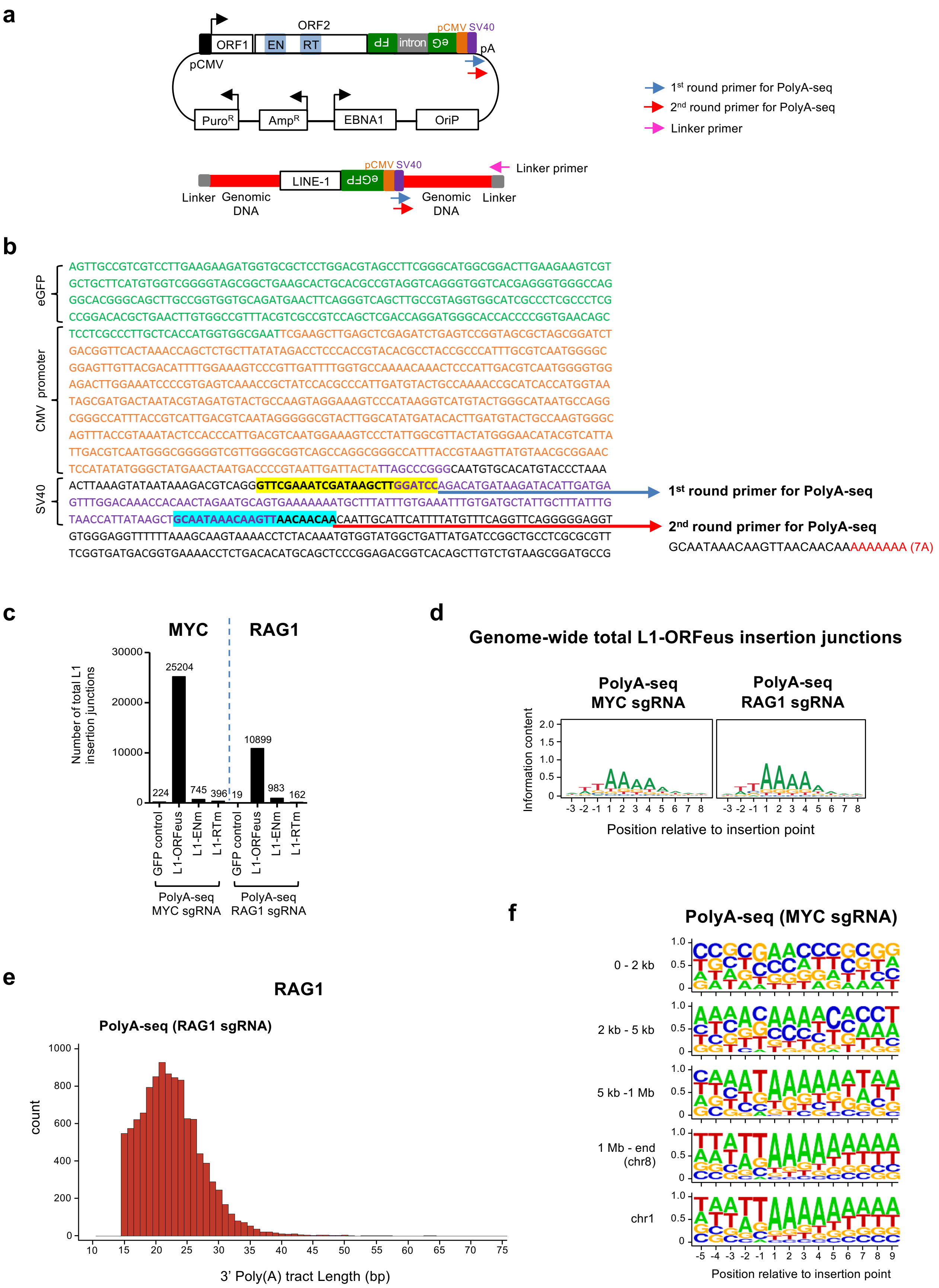

Supplementary Figure 4 to be continued

Supplementary Figure 4 Continued

g

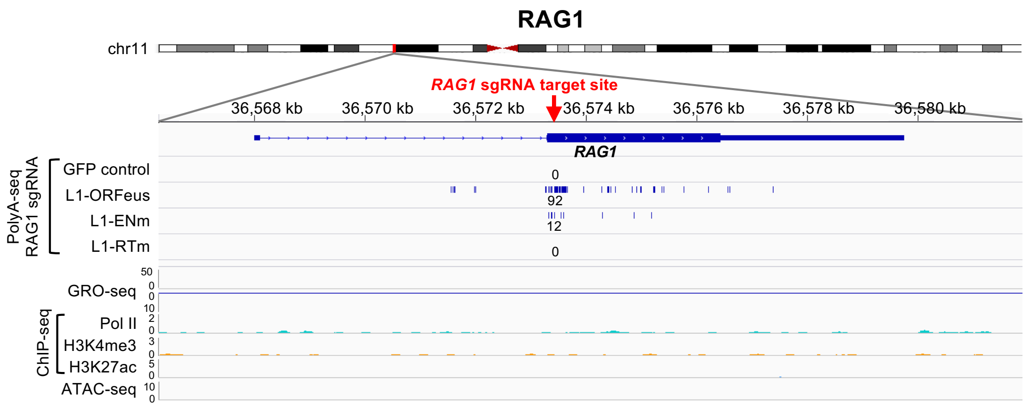

h

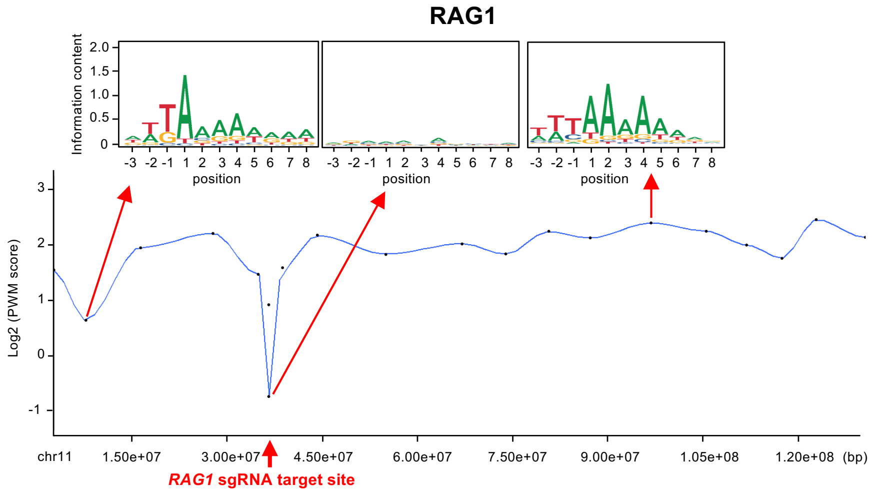

Supplementary Figure 4. Development of PolyA-seq to capture genome-wide *de novo* L1-ORFeus insertion events. a, Schematic representation of PolyA-seq by one-sided, nested amplification approach. The two-round forward primers were designed at the end of L1 GFP cassette (**a**, top), thus enable us to map the L1 retrotransposition sites genome-wide with the help of reverse linker primer (**a**, bottom). **b**, Detailed view of primers designed for PolyA-seq. PolyA-seq originates from SV40 PolyA and imposes a requirement for L1 insertions to have a minimal 15 bp Poly(A) tail (7A are imposed to primer directly and 8A are added during the sequence analysis). **c**, Numbers of total L1-ORFeus insertions mapped to the human genome obtained by PolyA-seq in HEK293T cells targeted by *MYC* CRISPR/Cas9 (**c**, left) or *RAG1* CRISPR/Cas9 (**c**, right) expressing L1-ORFeus, L1-ENm, L1-RTm or GFP control. Example sequences of PolyA-seq from *MYC* CRISPR editing in HEK293T cells expressing L1-ORFeus are shown in **Supplementary Sequence 11. d**, Sequence logo representing the consensus motif detected at L1 pre-integration sites obtained by PolyA-seq in HEK293T cells targeted by *MYC* CRISPR/Cas9 (**d**, left) or *RAG1* CRISPR/Cas9 (**d**, right) expressing L1-ORFeus. **e**, Distribution of 3' Poly(A) tract lengths of L1 insertions obtained by PolyA-seq in HEK293T cells expressing L1-ORFeus and targeted by *RAG1* CRISPR/Cas9. **f**, Sequence logo of L1 EN consensus motif surrounding *MYC* CRISPR/Cas9 target site obtained by PolyA-seq in HEK293T cells targeted by *MYC* CRISPR/Cas9 expressing L1-ORFeus. **g**, Detailed view of the distribution of L1-ORFeus insertions at the *RAG1* locus obtained by PolyA-seq in HEK293T cells targeted by *RAG1* CRISPR/Cas9 expressing L1-ORFeus, L1-ENm, L1-RTm or GFP control (**g**, top). Corresponding GRO-seq, ChIP-seq (including Pol II, H3K4me3 and H3K27ac) and ATAC-seq profiles are shown (**g**, bottom). **h**, Sequence logo representing the consensus motif in example regions proximal or distant to the *RAG1* CRISPR/Cas9 DSB.

Supplementary Figure 5

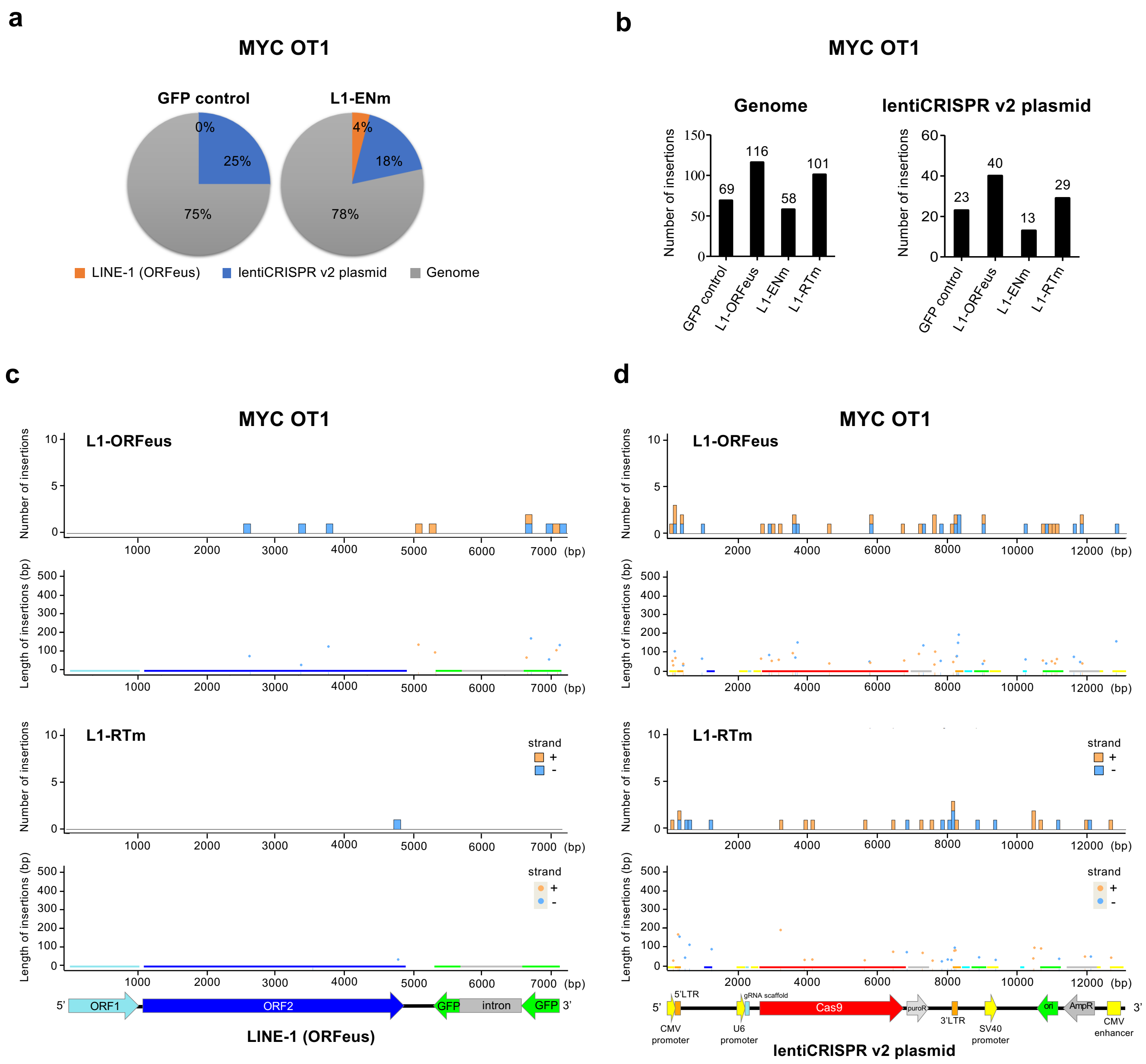

Supplementary Figure 5. RT-dependent *de novo* L1-ORFeus insertions at CRISPR/Cas9 off-target sites in HEK293T cells. a, Pie charts show the relative abundance of indicated three main types of insertions (i.e., L1-ORFeus, lentiCRISPR v2 plasmid and genomic fragments) obtained by amplicon sequencing at the MYC OT1 locus in HEK293T cells targeted by *MYC* CRISPR/Cas9 expressing GFP control or L1-ENm. **b**, Numbers of insertions mapped to the human genome (**b**, left) or lentiCRISPR v2 plasmid (**b**, right) obtained by amplicon sequencing at the MYC OT1 locus in HEK293T cells targeted by *MYC* CRISPR/Cas9 expressing L1-ORFeus, L1-ENm, L1-RTm or GFP control. **c,d**, Numbers and fragment lengths of L1-ORFeus insertions (**c**) or lentiCRISPR v2 plasmid insertions (**d**) obtained by amplicon sequencing at the MYC OT1 locus in HEK293T cells targeted by *MYC* CRISPR/Cas9 expressing L1-ORFeus or L1-RTm. The orientation of insertions is shown in orange when the fragment is oriented 5' to 3' (+) or blue when 3' to 5' (-).

Supplementary Figure 6

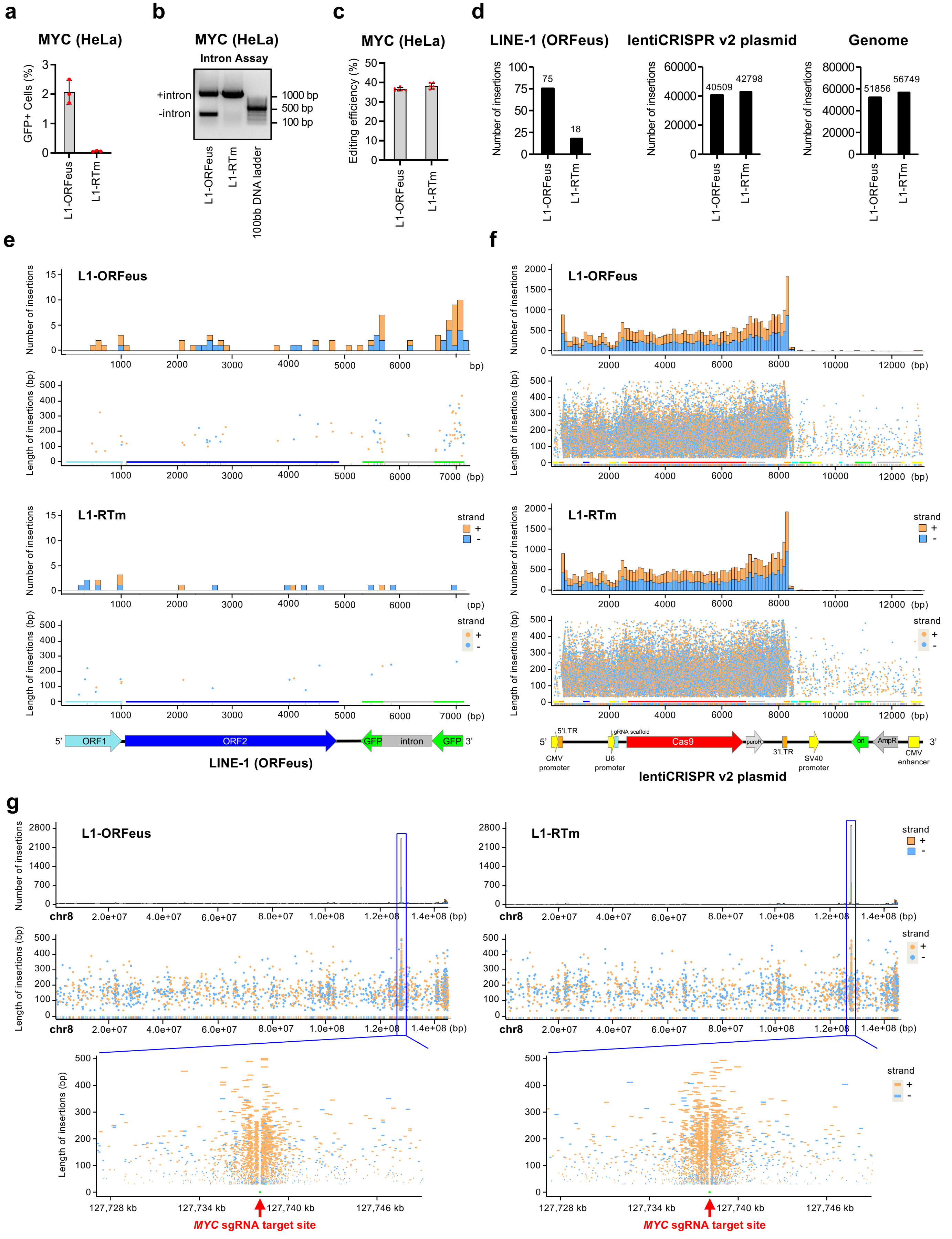

Supplementary Figure 6 to be continued

Supplementary Figure 6 Continued

h

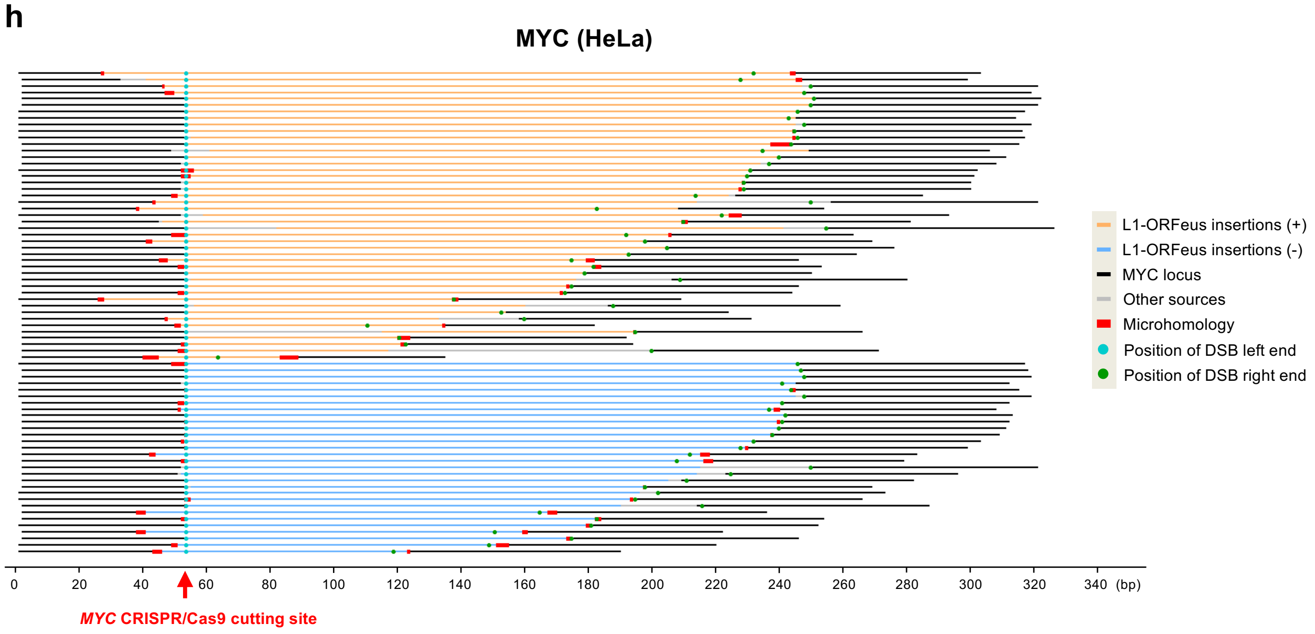

i

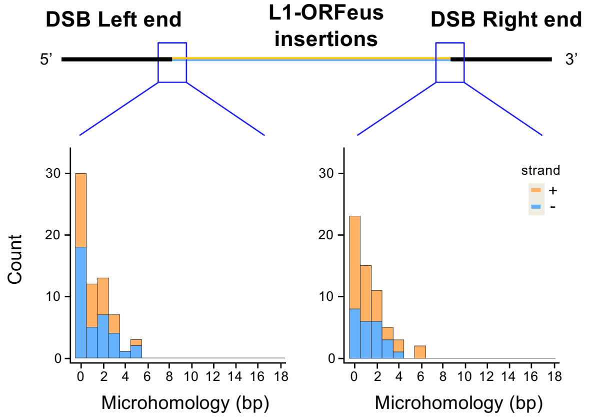

j

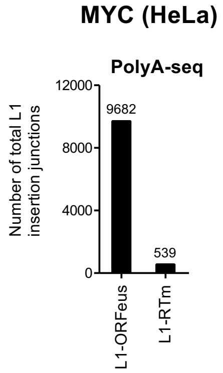

k

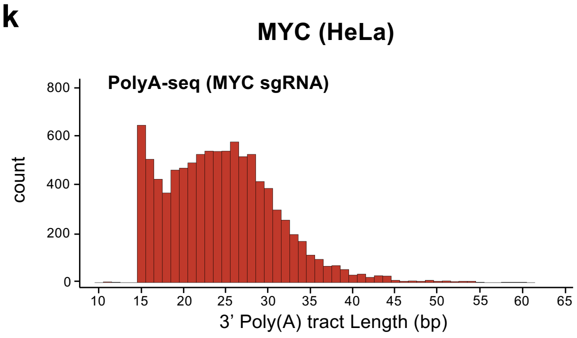

Supplementary Figure 6. Detailed characterization of *de novo* L1-ORFeus insertions at CRISPR/Cas9 target site in HeLa cells. a,**b**, Percentage of GFP-positive HeLa cells at day 11 after L1-ORFeus transfection and introducing CRISPR/Cas9-mediated DSBs at the *MYC* locus (**a**) (Data are Mean±SD, n=3 independent experiments); Intron assay was then performed to probe the L1-ORFeus retrotransposition events by genomic DNA-PCR (**b**). **c**, Editing efficiency of CRISPR/Cas9 at the *MYC* target site in HeLa cells expressing L1-ORFeus or L1-RTm. Data are Mean±SD, n=6 independent experiments. **d**, Numbers of insertions mapped to LINE-1 (ORFeus) (**d**, left), lentiCRISPR v2 plasmid (**d**, middle) or the human genome (**d**, right) obtained by amplicon sequencing at the *MYC* locus in HeLa cells targeted by *MYC* CRISPR/Cas9 expressing L1-ORFeus or L1-RTm. **e-g**, Numbers and fragment lengths of L1-ORFeus insertions (**e**), lentiCRISPR v2 plasmid insertions (**f**) or chromosome 8 genomic insertions (**g**) obtained by amplicon sequencing at the *MYC* locus in HeLa cells targeted by *MYC* CRISPR/Cas9 expressing L1-ORFeus or L1-RTm. Example L1-ORFeus insertions bridging the intron of GFP obtained by amplicon sequencing at the *MYC* CRISPR/Cas9 target site in HeLa cells expressing L1-ORFeus are shown in **Supplementary Sequence 9**. **h**, L1-ORFeus junction analysis obtained by amplicon sequencing at the *MYC* locus targeted by *MYC* CRISPR/Cas9 in HeLa cells expressing L1-ORFeus. The sequence alignment was centered on the left end of CRISPR-Cas9-mediated DSBs. Other sources include genomic fragments or plasmid insertions. **i**, Histogram plots of microhomology lengths in junctions (data from **h**) joining L1-ORFeus to the left or right end of the CRISPR/Cas9-mediated DSB in HeLa cells expressing L1-ORFeus. **j**, Numbers of total L1-ORFeus insertions mapped to the human genome obtained by PolyA-seq in HeLa cells targeted by *MYC* CRISPR/Cas9 expressing L1-ORFeus or L1-RTm. **k**, Distribution of 3' Poly(A) tract lengths of L1-ORFeus insertions obtained by PolyA-seq in HeLa cells expressing L1-ORFeus and targeted by *MYC* CRISPR/Cas9. Source data are provided as a Source Data file.

Supplementary Figure 7

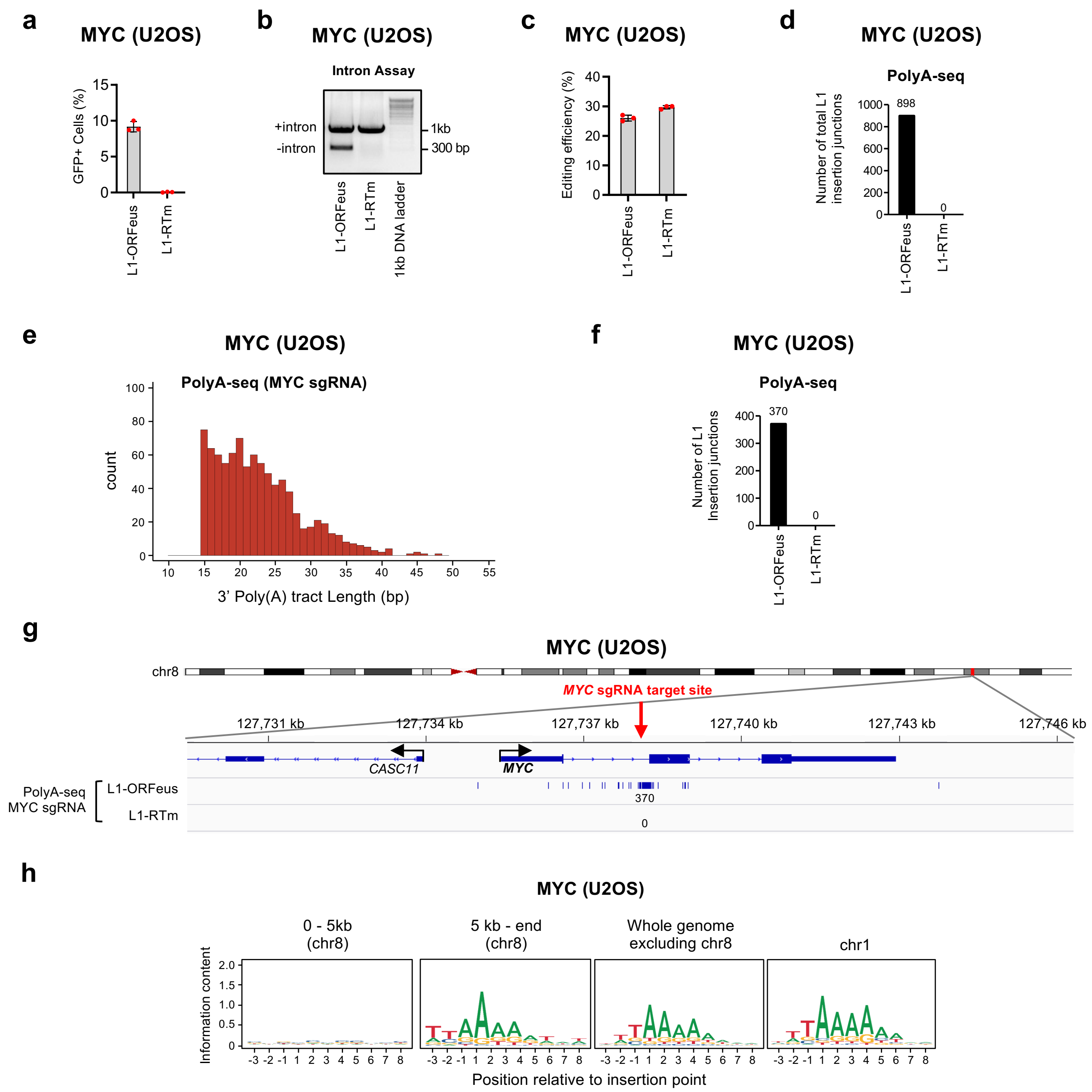

Supplementary Figure 7. RT-dependent *de novo* L1-ORFeus insertions at CRISPR/Cas9 target site in U2OS cells. a, Percentage of GFP-positive U2OS cells at day 11 after L1-ORFeus transfection and CRISPR/Cas9-mediated DSBs at the *MYC* locus. Data are Mean±SD, n=3 independent experiments. **b**, Intron assay was then performed. **c**, Editing efficiency of CRISPR/Cas9 at the *MYC* target site in U2OS cells expressing L1-ORFeus or L1-RTm. Data are Mean±SD, n=3 independent experiments. **d**, Total L1-ORFeus insertions mapped to the human genome obtained by PolyA-seq in U2OS cells targeted by *MYC* CRISPR/Cas9 and expressing L1-ORFeus or L1-RTm. **e**, Distribution of 3' Poly(A) tract lengths of L1-ORFeus insertions obtained by PolyA-seq in U2OS cells targeted by *MYC* CRISPR/Cas9 and expressing L1-ORFeus. **f**, Numbers of L1-ORFeus insertions at the *MYC* locus obtained by PolyA-seq targeted by *MYC* CRISPR/Cas9 in U2OS cells that express L1-ORFeus or L1-RTm. **g**, Detailed view of the distribution of L1-ORFeus insertions at the *MYC* locus obtained by PolyA-seq in U2OS cells targeted by *MYC* CRISPR/Cas9 and expressing L1-ORFeus or L1-RTm. **h**, Sequence logos representing the consensus motif detected at L1-ORFeus pre-integration sites in indicated regions proximal or distant to the *MYC* CRISPR/Cas9 DSB in chromosome 8. The canonical consensus motif in chromosome 1 served as a control. Source data are provided as a Source Data file.

Supplementary Figure 8

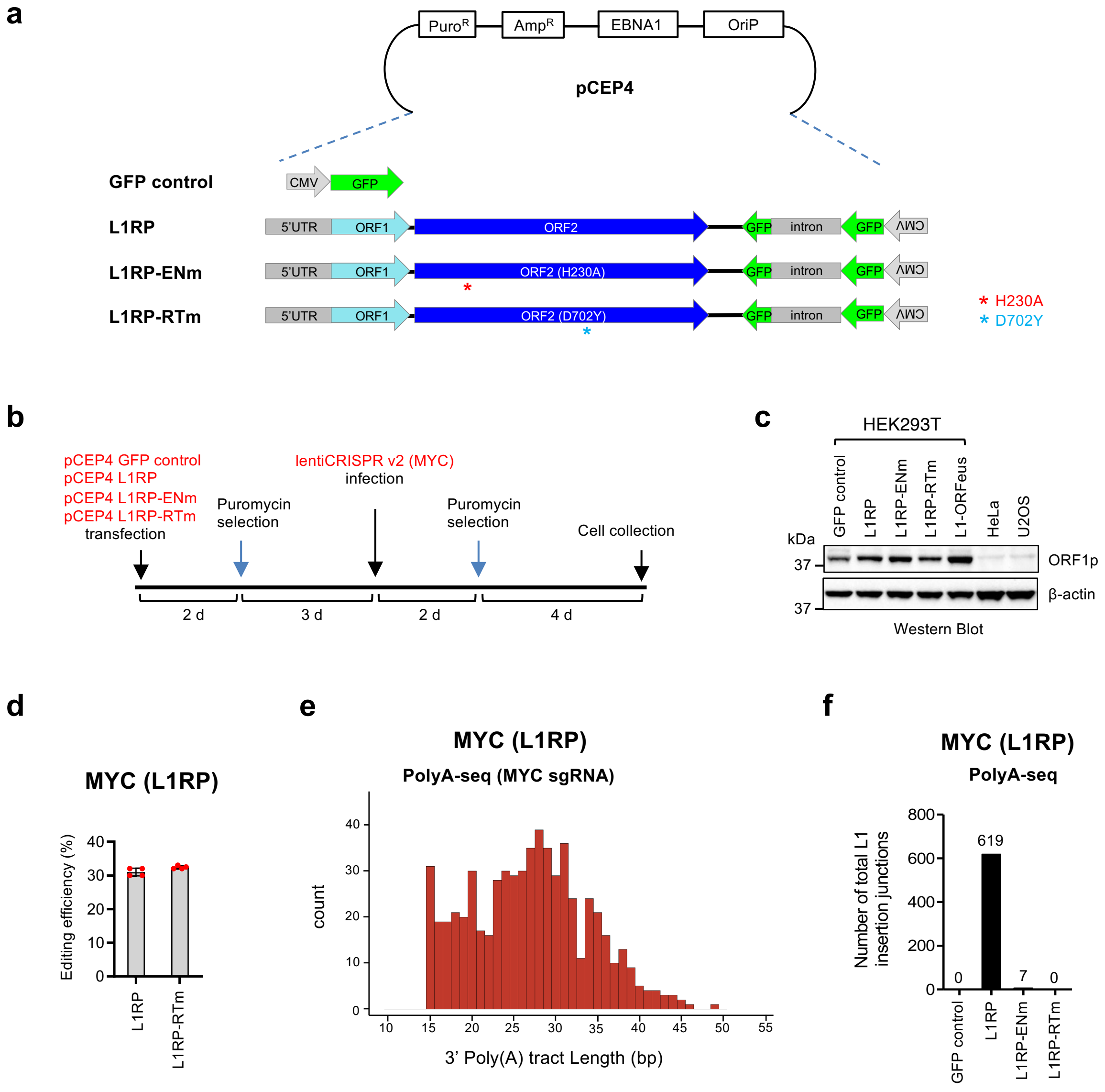

Supplementary Figure 8. RT-dependent *de novo* L1RP insertions at CRISPR/Cas9 target site in HEK293T cells. a, Schematic representation of pCEP4 GFP control, pCEP4 L1RP, pCEP4 L1RP-ENm (H230A) (L1RP-ENm), and pCEP4 L1RP-RTm (D702Y) (L1RP-RTm) plasmids. **b**, Schematic of the experimental strategy employed to examine *de novo* L1RP retrotransposition events at the *MYC* CRISPR/Cas9 target site in HEK293T cells. PolyA-seq is performed on the bulk population of cells without GFP sorting. **c**, HEK293T cells were transfected with 1 μg L1RP reporters or L1-ORFeus reporter, and Western Blot was performed to detect L1 ORF1p protein; β-actin expression served as loading control. **d**, Editing efficiency of CRISPR/Cas9 at the *MYC* target site in HEK293T cells expressing L1RP or L1RP-RTm. Data are Mean±SD, n=4 independent experiments. **e**, Distribution of 3' Poly(A) tract lengths of L1RP insertions obtained by PolyA-seq in HEK293T cells expressing L1RP and targeted by *MYC* CRISPR/Cas9. **f**, Numbers of total L1RP insertions mapped to the human genome obtained by PolyA-seq in HEK293T cells targeted by *MYC* CRISPR/Cas9 and expressing L1RP, L1RP-ENm, L1RP-RTm or GFP control. Source data are provided as a Source Data file.

Supplementary Figure 9

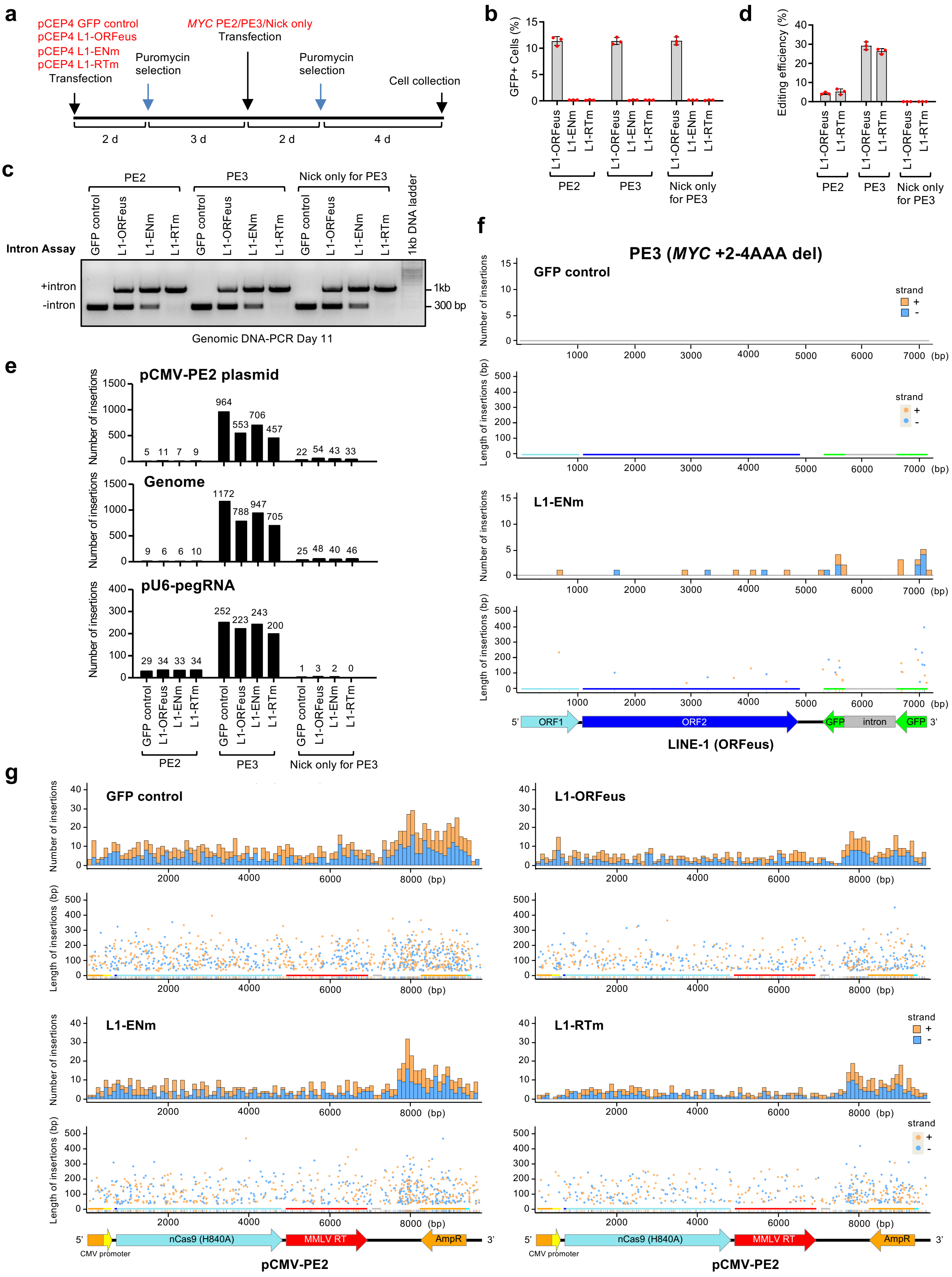

Supplementary Figure 9 to be continued

Supplementary Figure 9 Continued

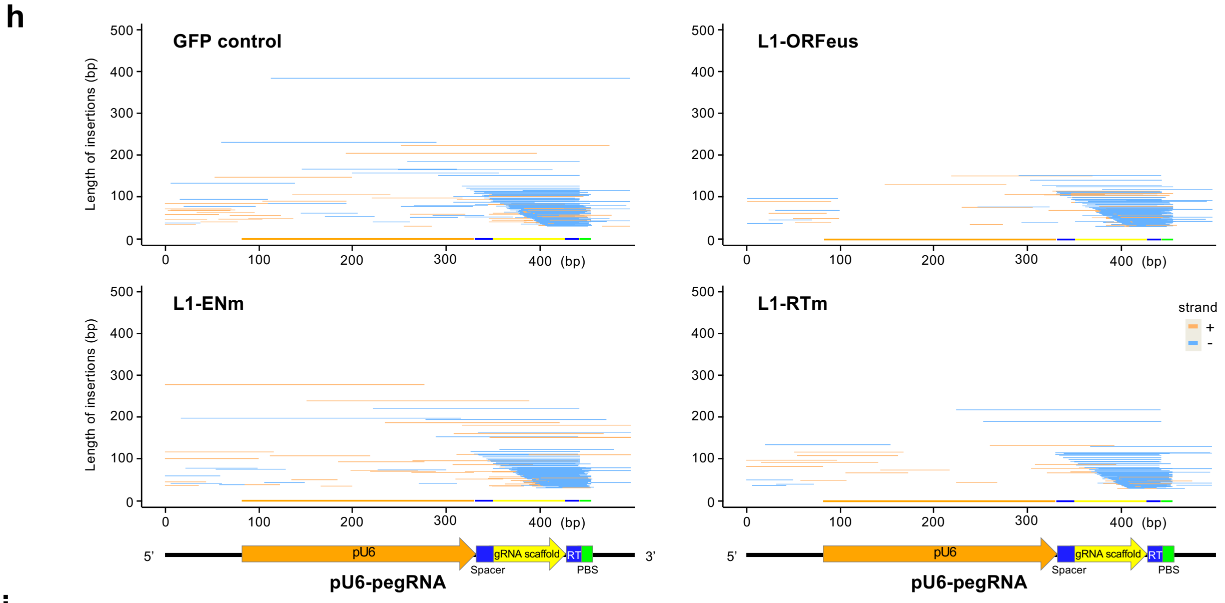

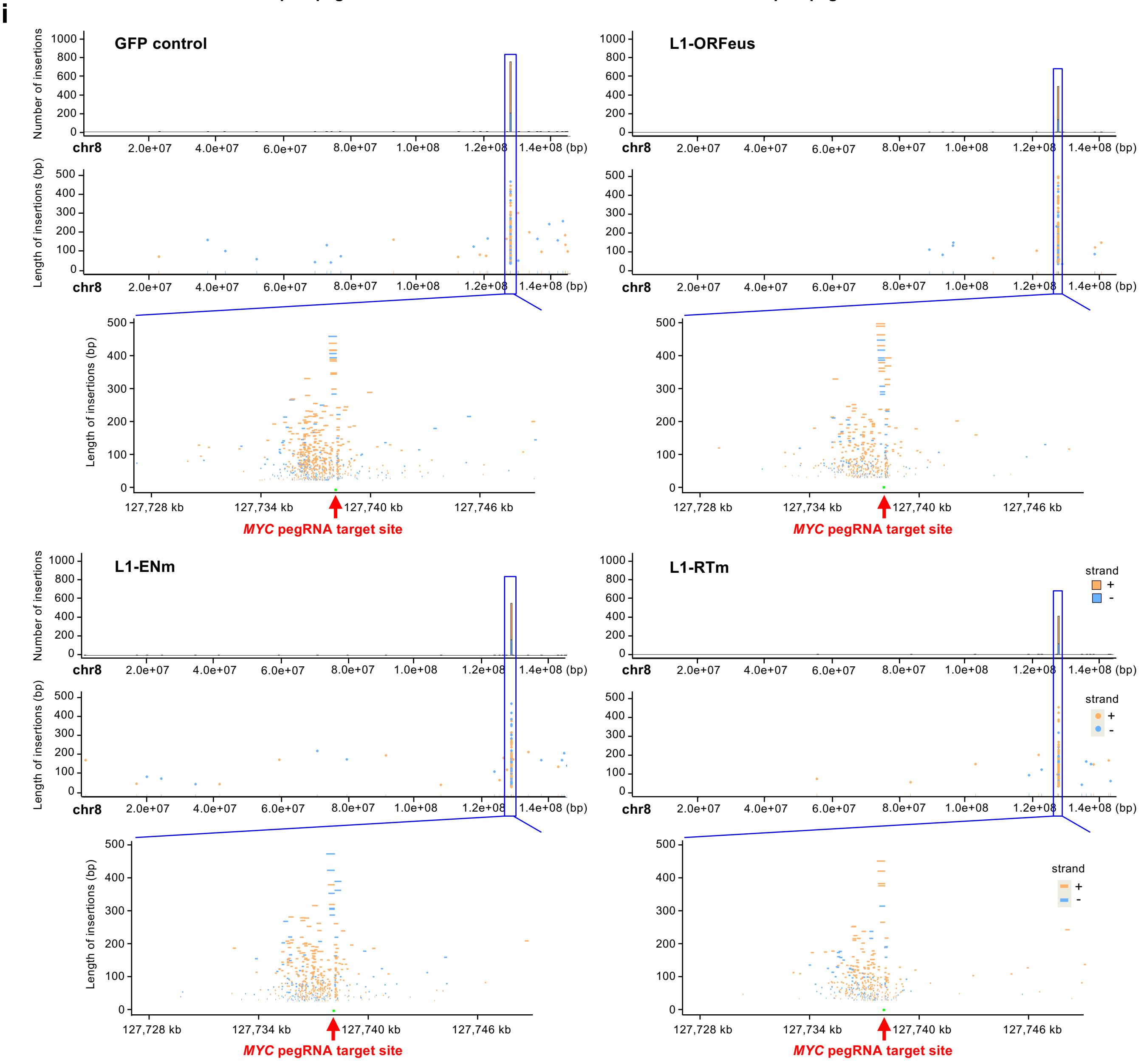

Supplementary Figure 9. Safety evaluation of prime editing at the *MYC* (+2-4AAA del) site in HEK293T cells. a, Similar to **Fig. 1b**, schematic of the experimental strategy employed to examine *de novo* L1-ORFeus retrotransposition events at the *MYC* prime editing site in HEK293T cells. **b**,**c**, Percentage of GFP-positive HEK293T cells at day 11 after L1-ORFeus transfection and introducing PE2 (*MYC* +2-4AAA del), PE3 (*MYC* +2-4AAA del) or nick only for PE3 (**b**) (Data are Mean±SD, n=3 independent experiments), Intron assay was then performed to probe the L1-ORFeus retrotransposition events by genomic DNA-PCR (**c**). **d**, Prime editing efficiency of PE2 (*MYC* +2-4AAA del), PE3 (*MYC* +2-4AAA del) or nick only for PE3 at the *MYC* locus in HEK293T cells expressing L1-ORFeus or L1-RTm. Data are Mean±SD, n=3 independent experiments. **e**, Numbers of insertions mapped to pCMV-PE2 plasmid (**e**, top), the human genome (**e**, middle) or pU6-pegRNA (**e**, bottom) obtained by amplicon sequencing at the *MYC* locus in HEK293T cells targeted by PE2 (*MYC* +2-4AAA del), PE3 (*MYC* +2-4AAA del) or nick only for PE3 expressing L1-ORFeus, L1-ENm, L1-RTm or GFP control. **f-i**, Numbers and fragment lengths of L1-ORFeus insertions (**f**), pCMV-PE2 plasmid insertions (**g**), pU6-pegRNA insertions (**h**) or chromosome 8 insertions (**i**) obtained by amplicon sequencing at the *MYC* locus in HEK293T cells targeted by PE3 (*MYC* +2-4AAA del) expressing L1-ORFeus, L1-ENm, L1-RTm or GFP control. The orientation of insertions is shown in orange when the fragment is oriented 5' to 3' (+) or blue when 3' to 5' (-). Source data are provided as a Source Data file.

Supplementary Figure 10

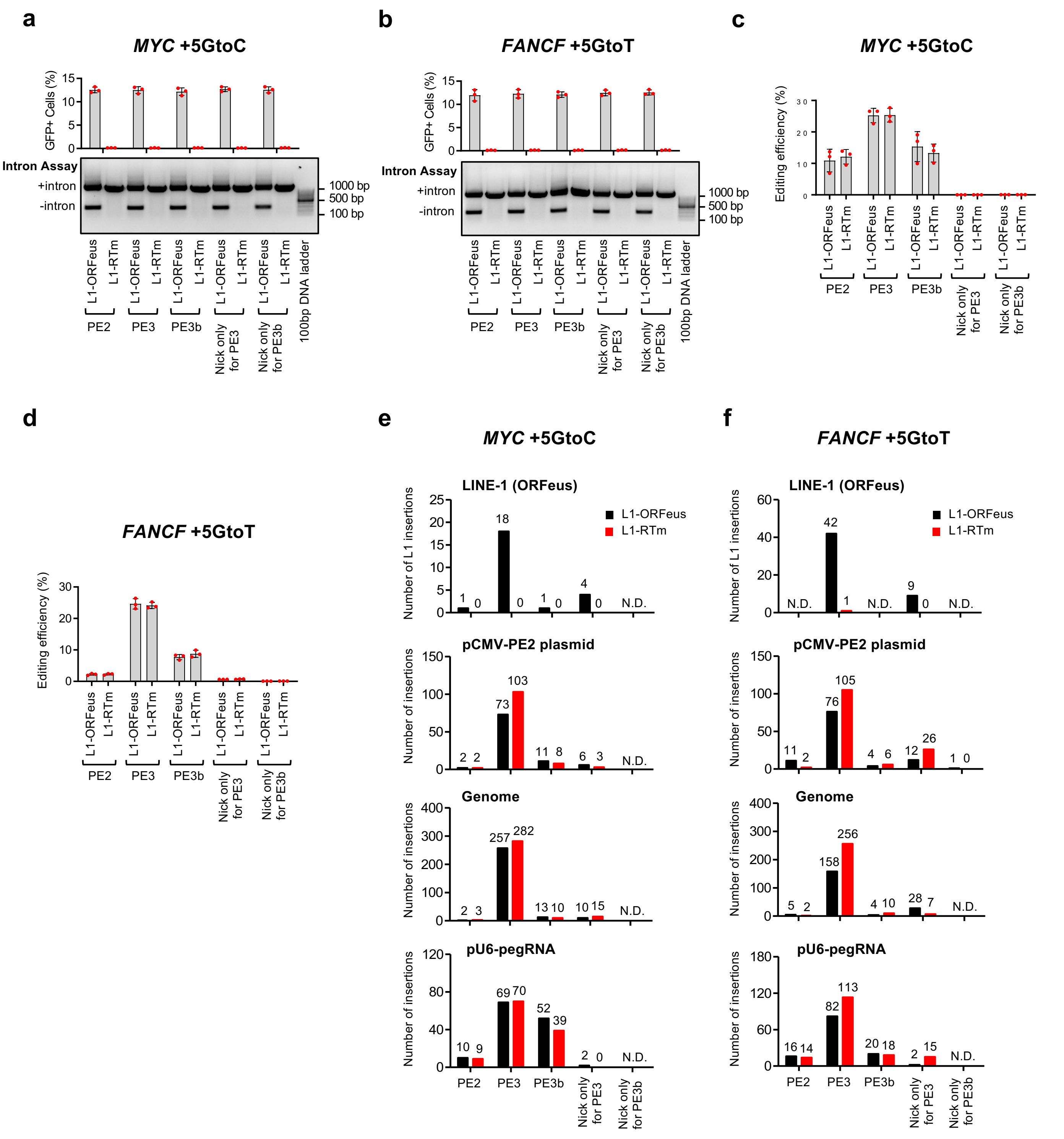

Supplementary Figure 10. Safety evaluation of prime editing at the *MYC* (+5GtoC) or *FANCF* (+5GtoT) site in HEK293T cells. a, Percentage of GFP-positive HEK293T cells at day 11 after L1-ORFeus transfection and introducing PE2 (*MYC* +5GtoC), PE3 (*MYC* +5GtoC), PE3b (*MYC* +5GtoC) or nick only for PE3/PE3b (**a**, top). Data are Mean±SD, n=3 independent experiments. Intron assay was performed to probe the L1-ORFeus retrotransposition events by genomic DNA-PCR at day 11 (**a**, bottom). **b**, Percentage of GFP-positive HEK293T cells at day 11 after L1-ORFeus transfection and introducing PE2 (*FANCF* +5GtoT), PE3 (*FANCF* +5GtoT), PE3b (*FANCF* +5GtoT) or nick only for PE3/PE3b (**b**, top). Data are Mean±SD, n=3 independent experiments. Intron assay was then performed (**b**, bottom). **c**,**d**, Precise prime editing efficiency of the indicated prime editors at the *MYC* +5GtoC (**c**) or *FANCF* +5GtoT (**d**) site in HEK293T cells expressing L1-ORFeus or L1-RTm. Data are Mean±SD, n=3 independent experiments. **e**,**f**, Numbers of insertions mapped to LINE-1 (ORFeus), pCMV-PE2 plasmid, the human genome or pU6-pegRNA in HEK293T cells expressing L1-ORFeus or L1-RTm. Amplicon sequencing was analyzed at the *MYC* locus targeted by PE2 (*MYC* +5GtoC), PE3 (*MYC* +5GtoC), PE3b (*MYC* +5GtoC) or nick only for PE3/PE3b (**e**) or at the *FANCF* locus targeted by PE2 (*FANCF* +5GtoT), PE3 (*FANCF* +5GtoT), PE3b (*FANCF* +5GtoT) or nick only for PE3/PE3b (**f**). Source data are provided as a Source Data file.

Supplementary Figure 11

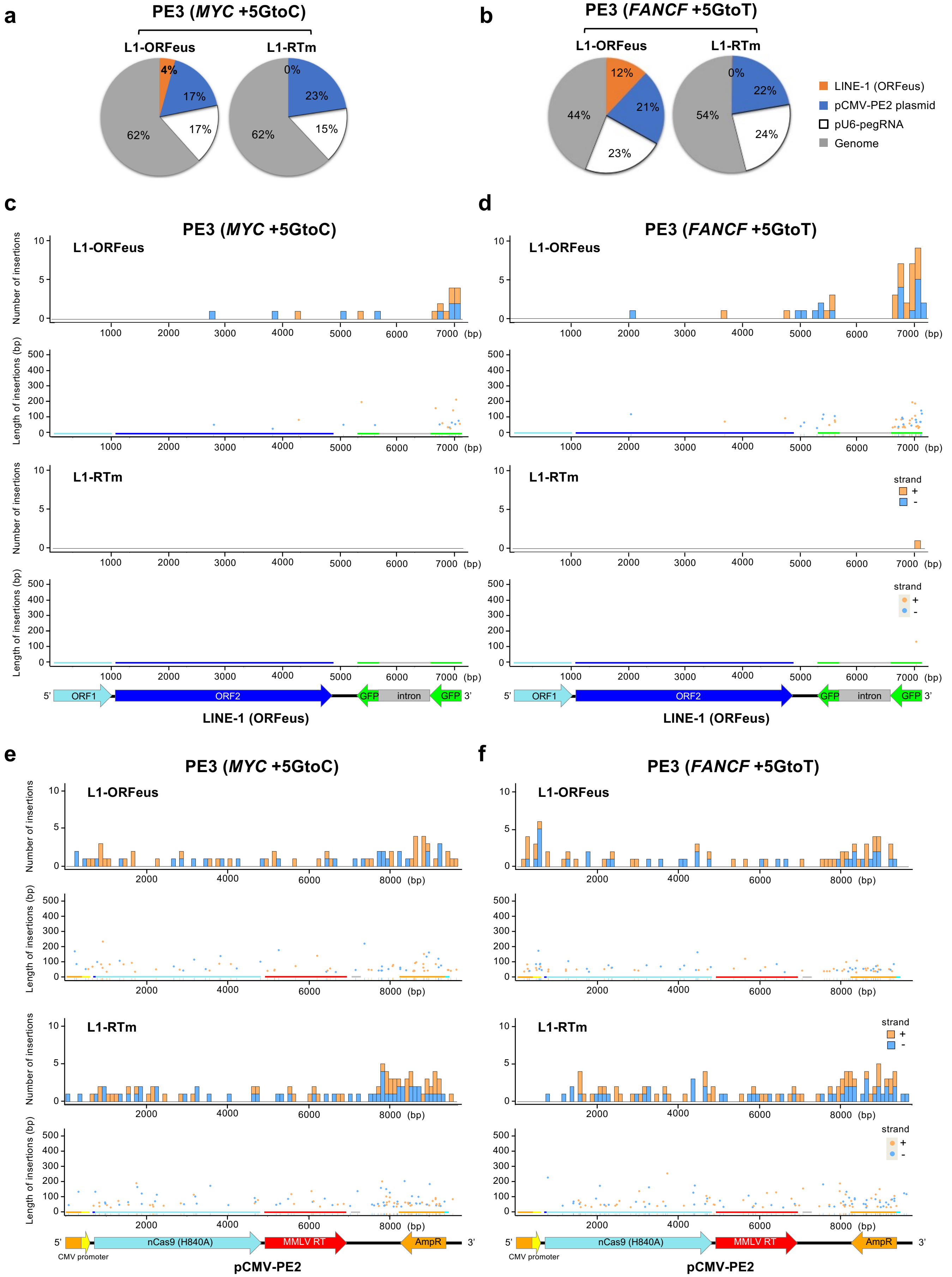

Supplementary Figure 11 to be continued

Supplementary Figure 11 Continued

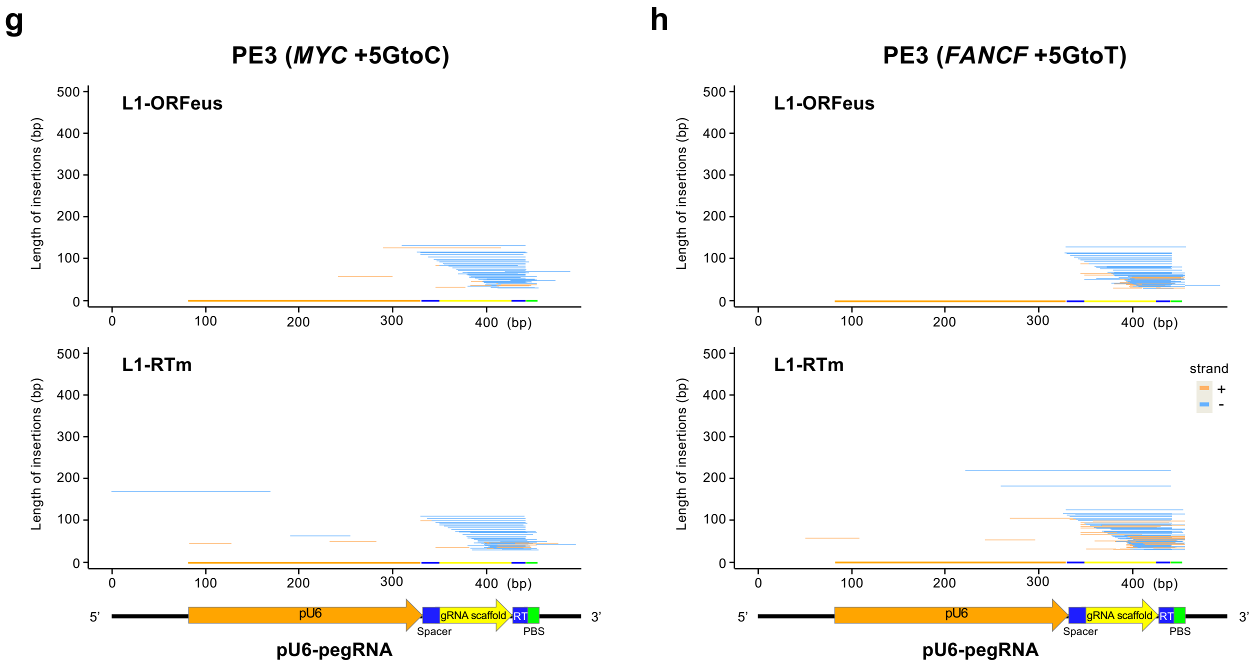

Supplementary Figure 11. Characterization of PE3 (*MYC* +5GtoC) and PE3 (*FANCF* +5GtoT) editing in HEK293T cells. a,**b**, Pie charts show the relative abundance of four main types of insertions (i.e., L1-ORFeus, pCMV-PE2 plasmid, pU6-pegRNA or genomic fragments) obtained by amplicon sequencing at the *MYC* locus targeted by PE3 (*MYC* +5GtoC) (**a**) or at the *FANCF* locus targeted by PE3 (*FANCF* +5GtoT) (**b**) in HEK293T cells expressing L1-ORFeus or L1-RTm. **c-h**, Numbers and fragment lengths of L1-ORFeus insertions (**c**,**d**), pCMV-PE2 plasmid insertions (**e**,**f**) or pU6-pegRNA insertions (**g**,**h**) obtained by amplicon sequencing at the *MYC* locus targeted by PE3 (*MYC* +5GtoC) (**c**,**e**,**g**) or PE3 (*FANCF* +5GtoT) (**d**,**f**,**h**) in HEK293T cells expressing L1-ORFeus or L1-RTm. The orientation of insertions is shown in orange when the fragment is oriented 5' to 3' (+) or blue when 3' to 5' (-).

Supplementary Figure 12

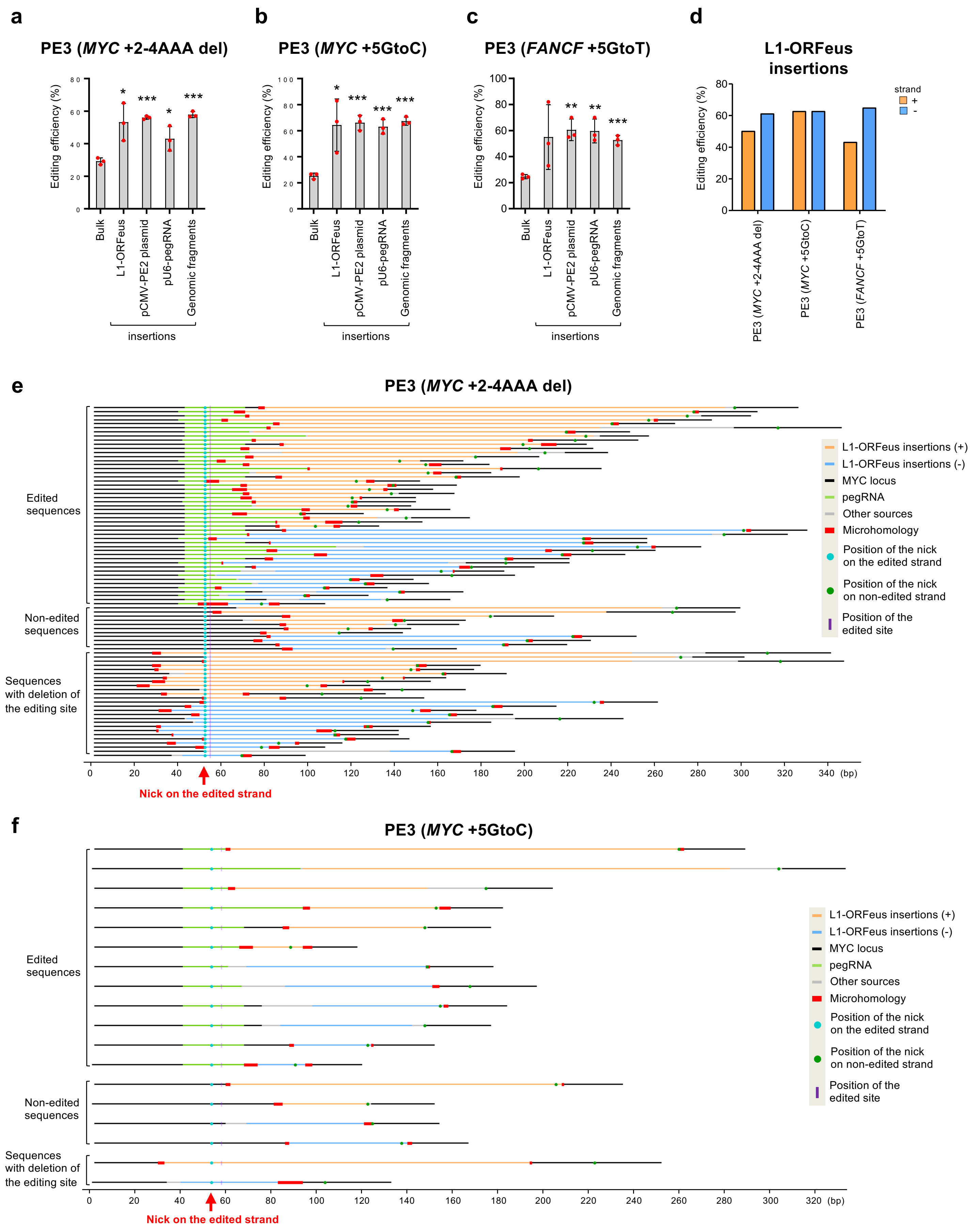

Supplementary Figure 12 to be continued

Supplementary Figure 12 Continued

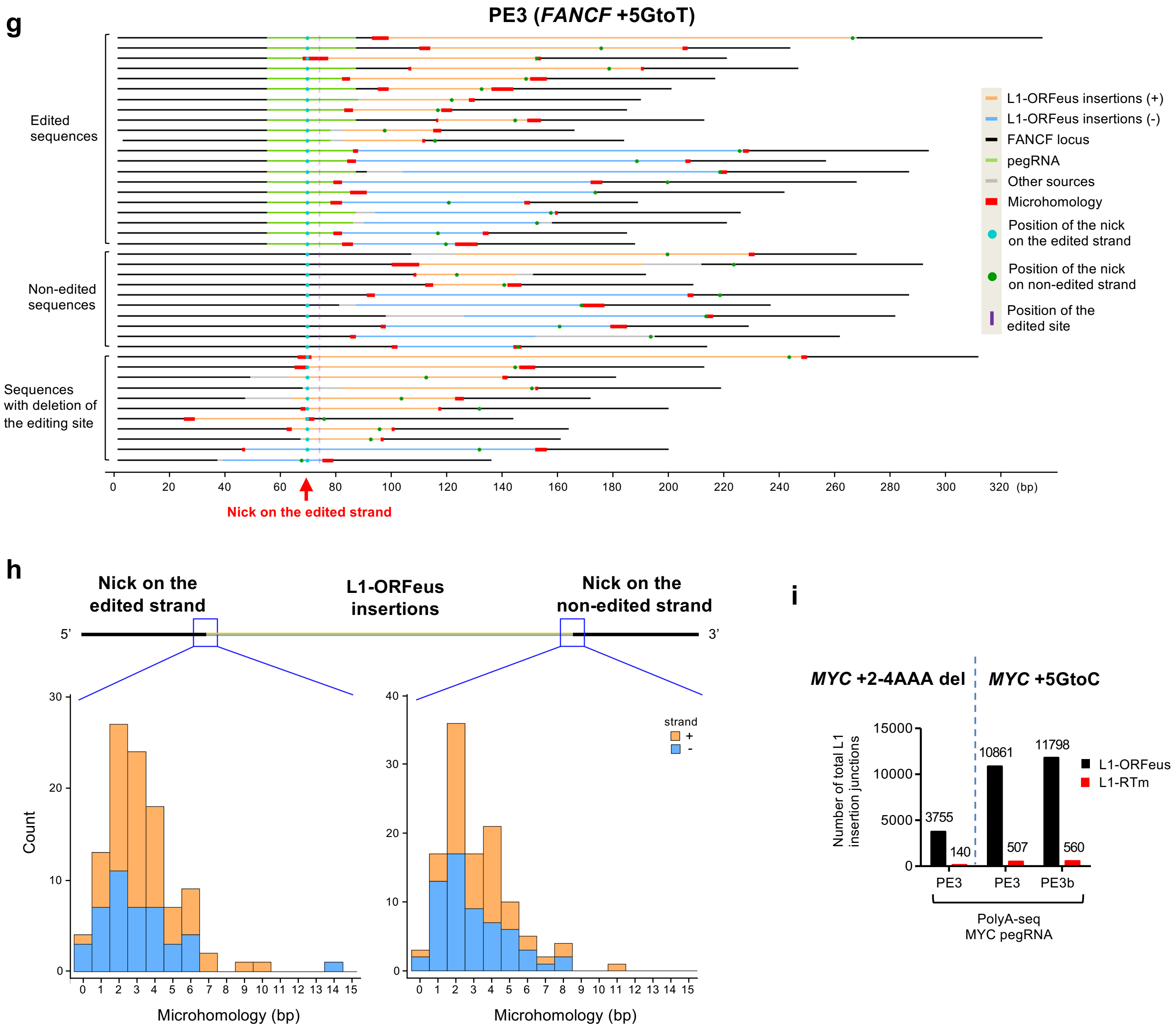

Supplementary Figure 12. Characterization of *de novo* L1-ORFeus insertions in PE3 edited HEK293T cells. a-c, Prime editing efficiency in sequences with four indicated types of insertions compared to the bulk sequences. Amplicon sequencing was analyzed in HEK293T cells expressing L1-ORFeus at the *MYC* locus targeted by PE3 (*MYC* +2-4AAA del) (**a**) or PE3 (*MYC* +5GtoC) (**b**), or at the *FANCF* locus targeted by PE3 (*FANCF* +5GtoT) (**c**). Data are Mean±SD, n=3 independent experiments. * p <0.05, ** p <0.005, *** p <0.0005. **d**, Prime editing efficiency was independent of the L1-ORFeus insertion orientation. **e-g**, L1-ORFeus junction analysis obtained by amplicon sequencing at the *MYC* locus targeted by PE3 (*MYC* +2-4AAA del) (**e**) or PE3 (*MYC* +5GtoC) (**f**), or at the *FANCF* locus targeted by PE3 (*FANCF* +5GtoT) (**g**) in HEK293T cells expressing L1-ORFeus. The sequence alignment was centered on the pegRNA-mediated nick site. Other sources include genomic fragments or plasmid insertions. Edited sequences: sequences containing the pegRNA-edited site; non-edited sequences: sequences containing wild type MYC; sequences with deletion of the editing site: sequences in which the editing site was deleted during L1-ORFeus insertion. **h**, Histogram plots of microhomology lengths in junctions (pooled from **e-g**) joining L1-ORFeus to pegRNA-mediated nicking site (on the edited strand) or sgRNA-mediated nicking site (on the non-edited strand) in HEK293T cells expressing L1-ORFeus. **i**, Numbers of total L1-ORFeus insertions mapped to the human genome obtained by PolyA-seq in cells targeted by PE3 (*MYC* +2-4AAA del), PE3 (*MYC* +5GtoC) or PE3b (*MYC* +5GtoC) expressing L1-ORFeus or L1-RTm. Source data are provided as a Source Data file.

Supplementary Figure 13

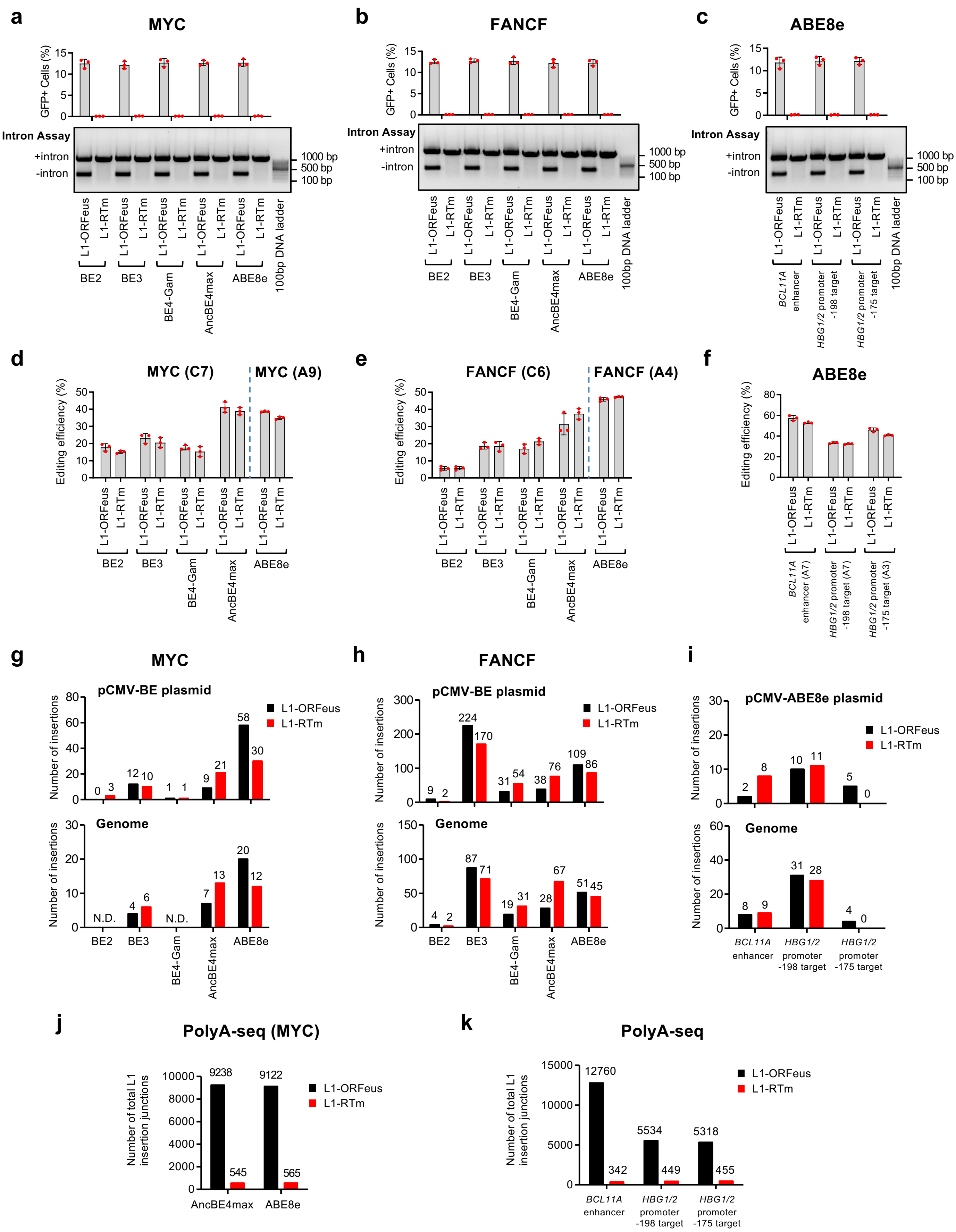

Supplementary Figure 13 to be continued

Supplementary Figure 13 Continued

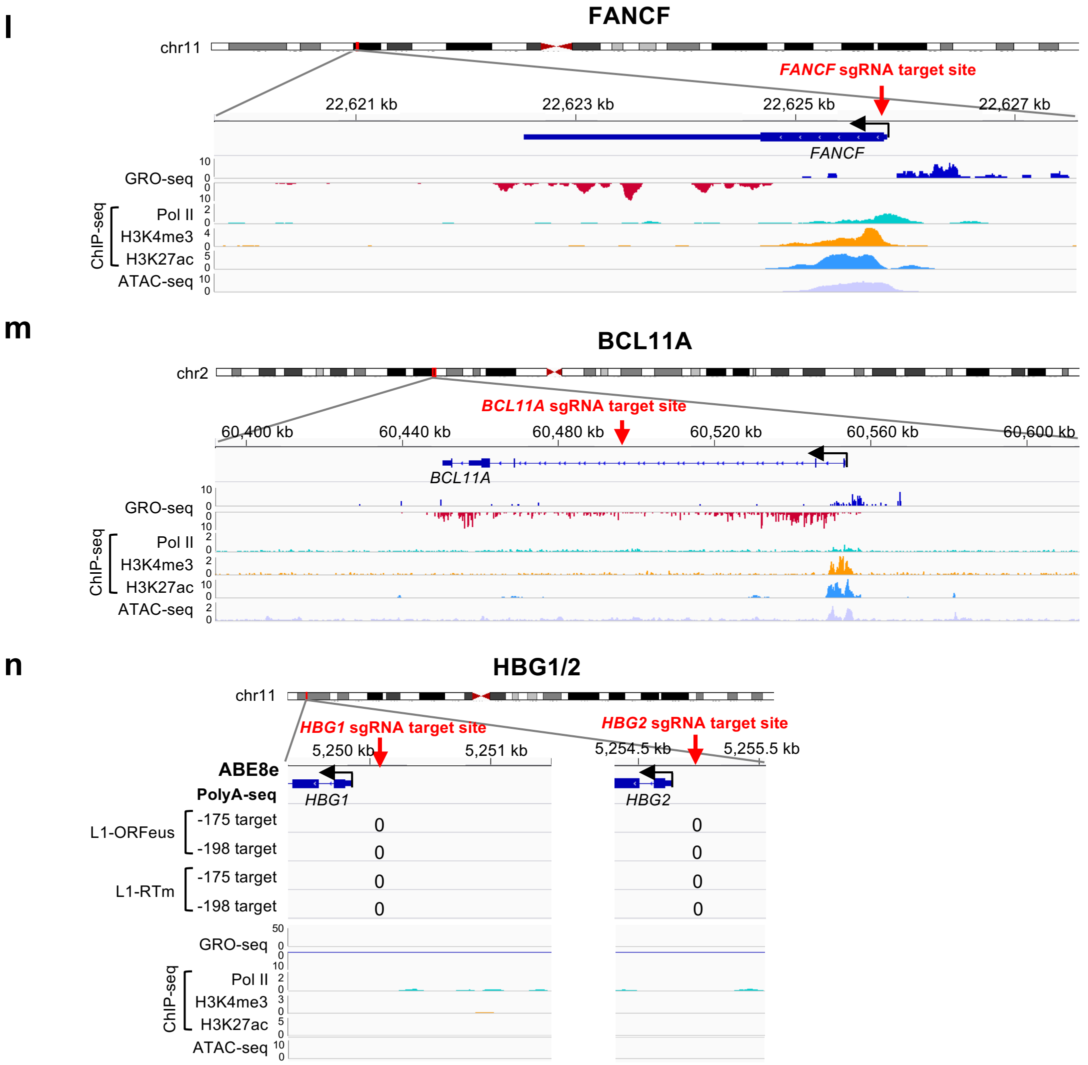

Supplementary Figure 13. *De novo* L1-ORFeus insertions are rare in base editing in HEK293T cells. **a**,**b**, Percentage of GFP-positive HEK293T cells at day 11 after L1-ORFeus transfection and introducing BE2, BE3, BE4-Gam, AncBE4max or ABE8e editing at the *MYC* (**a**, top) or *FANCF* locus (**b**, top). Data are Mean±SD, n=3 independent experiments. Intron assay was then performed (**a**,**b**, bottom). **c**, GFP-positive HEK293T cells were analyzed after introducing ABE8e editing at the *BCL11A* enhancer or *HBG1/2* promoter (-198bp/-175bp target) sites (**c**, top), Intron assay was then performed (**c**, bottom). Data are Mean±SD, n=3 independent experiments. **d**,**e**, Base editing efficiency at the *MYC* (**d**) or *FANCF* locus (**e**) in HEK293T cells expressing L1-ORFeus or L1-RTm. Data are Mean±SD, n=3 independent experiments. **f**, ABE8e editing efficiency at the *BCL11A* enhancer or *HBG1/2* promoter sites in HEK293T cells expressing L1-ORFeus or L1-RTm. Data are Mean±SD, n=3 independent experiments. **g**,**h**, Numbers of insertions mapped to indicated pCMV-BE plasmid or the human genome obtained by amplicon sequencing at the *MYC* (**g**) or *FANCF* (**h**) base editing site in HEK293T cells targeted by BE2, BE3, BE4-Gam, AncBE4max or ABE8e expressing L1-ORFeus or L1-RTm. **i**, Numbers of insertions mapped to pCMV-ABE8e plasmid or the human genome obtained by amplicon sequencing at the *BCL11A* enhancer or *HBG1/2* promoter editing sites in HEK293T cells targeted by ABE8e expressing L1-ORFeus or L1-RTm. **j**, Numbers of total L1-ORFeus insertions obtained by PolyA-seq in HEK293T cells targeted by AncBE4max or ABE8e at the *MYC* base editing site expressing L1-ORFeus or L1-RTm. **k**, Numbers of total L1-ORFeus insertions obtained by PolyA-seq in HEK293T cells targeted by ABE8e at the *BCL11A* enhancer or *HBG1/2* promoter sites expressing L1-ORFeus or L1-RTm. **l**,**m**, GRO-seq, ChIP-seq and ATAC-seq profiles are shown at the *FANCF* (**l**) or *BCL11A* enhancer (**m**) base editing site. **n**, Detailed view of the distribution of L1-ORFeus insertions at the *HBG1/2* promoter base editing sites obtained by PolyA-seq in HEK293T cells targeted by ABE8e expressing L1-ORFeus or L1-RTm (**n**, top). GRO-seq, ChIP-seq and ATAC-seq profiles are shown (**n**, bottom). Source data are provided as a Source Data file.

Supplementary Figure 14

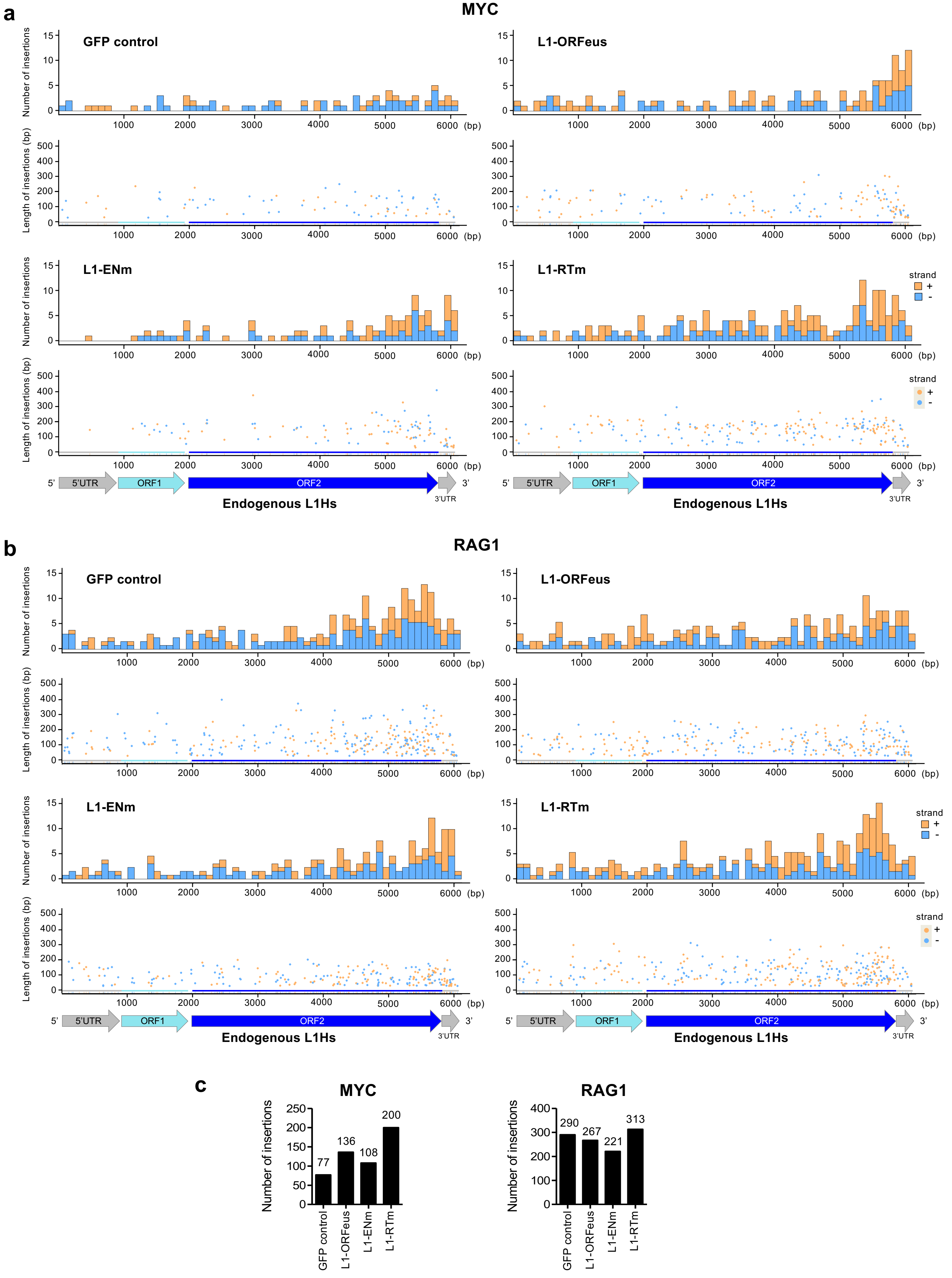

Supplementary Figure 14. Endogenous L1 sequences found at CRISPR/Cas9 target sites in HEK293T cells. **a**,**b**, Numbers of sequences mapped to the endogenous L1Hs sequences obtained by amplicon sequencing at the *MYC* locus targeted by *MYC* CRISPR/Cas9 (**a**) or at the *RAG1* locus targeted by *RAG1* CRISPR/Cas9 (**b**) in HEK293T cells. Two example L1 sequences are shown in **Supplementary Sequence 12**. **c**, Histogram showing numbers of L1Hs sequences found at *MYC* CRISPR/Cas9 target site (**c**, left) or *RAG1* CRISPR/Cas9 target site (**c**, right).

<u>Supplementary Tables</u>

Supplementary Table 1. Sequences of sgRNAs, pegRNAs and nicking sgRNAs (for PE3 or PE3b) used for mammalian cell experiments. All sequences are shown in 5' to 3' orientation.

Supplementary Table 2. Sequences of CRISPR off-targets (OT).

Supplementary Table 3. Primers for RT-PCR and Intron assay.

Supplementary Table 4. Primers for amplicon sequencing.

Supplementary Table 5. Primers for PolyA-seq.

Supplementary Table 1. Sequences of sgRNAs, pegRNAs, nicking sgRNAs (for PE3 or PE3b) and primers for amplifying target locus. All sequences are shown in 5' to 3' orientation.

1.1 sgRNAs in lentiCRISPR v2 vector (for canonical CRISPR/Cas9 genome editing)

Locus	Spacer sequence (PAM)	Chromosome (strand)	Start	End
MYC	GACTGTCCAAAGGGGGTGAAAGG	chr8 (+)	127,738,062	127,738,081
RAG1	GCCTCTTTCCCACCCACCTTGGG	chr11 (+)	36,573,311	36,573,330
CCR5	CAGAATTGATACTGACTGTATGG	chr3 (-)	46,373,452	46,373,471

1.2 sgRNAs in pU6-sgRNA vector (for Base Editors)

Locus	Spacer sequence (PAM)	Chromosome (strand)	Start	End
MYC	GACTGTCCAAAGGGGGTGAAAGG	chr8 (+)	127,738,062	127,738,081
FANCF	GGAATCCCTTCTGCAGCACCTGG	chr11 (-)	22,625,789	22,625,808
BCL11A enhancer	TTTATCACAGGCTCCAGGAAGGG	chr2 (-)	60,495,252	60,495,271
HBG1/2 promoter -198 target	GTGGGGAAGGGGCCCCCAAGAGG	chr11 (+)	5,250,049*<br>5,254,973#	5,250,068*<br>5,254,992#
HBG1/2 promoter -175 target	ATATTTGCATTGAGATAGTGTGG	chr11 (+)	5,250,030*<br>5,254,954#	5,250,049*<br>5,254,973#

**HBG1* promoter #*HBG2* promoter

1.3 pegRNAs in pU6-pegRNA vector (for Prime Editors)

pegRNA	Spacer sequence (PAM)	3' extension	PBS length (nt)	RT template length (nt)
MYC +2-4AAA del	GACTGTCCAAAGGGGGTGAAAGG	ATAAAGGGAGCACCCCACCCCCTTTGGAC	13	16
MYC +5GtoC	GACTGTCCAAAGGGGGTGAAAGG	AGGGAGCACCGTTTCACCCCCTTTGGAC	13	15
FANCF +5GtoT	GGAATCCCTTCTGCAGCACCTGG	GGAAAAGCGATCAAGGTGCTGCAGAAGGGATT	15	17

Mutated bases are listed in red

1.4 Nicking sgRNAs in pU6-pegRNA vector (for Prime Editors)

Nicking sgRNA	Purpose	Spacer sequence (PAM)
MYC +42	For PE3 (both *MYC* +2-4AAA del and *MYC* +5GtoC)	GAGCTATCCCCTAAAGCGGCTGG
MYC -5	For PE3b (*MYC* +5GtoC)	GAGCACCGTTTCACCCCCTTTGG
FANCF +48	For PE3	GGGGTCCCAGGTGCTGACGTAGG
FANCF +7	For PE3b	GAAGCTCGGAAAAGCGATCAAGG

Mutated bases are listed in red

1.5 Primers for amplifying target locus (for surveyor assay and/or sanger sequencing)

Locus	Forward primer	Reverse primer	PCR product length (bp)	Annealing temperature (℃)
MYC	ACTTTGTGCCTTGGATTTTGG	GCAGCAGCTCGAATTTCTTCCA	568	58
RAG1	GTAACCATAAACACTGTCAGAAGAGG	AGGACTGCTGGAGATTGCTC	407	57
CCR5	GGCTCTATTTTATAGGCTTCTTCTCT	TGGTCCAACCTGTTAGAGCTACTG	514	58
FANCF	GTAGGATGCCCTACATCTGCT	GTTCGCTAATCCCGGAACTGG	488	58
BCL11A enhancer	CTATGCCCCAGGTGTGCATAAG	CCATCACCAAGAGAGCCTTCCGA	301	59
HBG1/2 promoter	TGATAACCTCAGACGTTCCAG	CTGACAAAAGAAGTCCTGGTATC	378* / 374#	56

**HBG1* promoter #*HBG2* promoter

Supplementary Table 2. Sequences of CRISPR off-targets (OT).

CRISPR target	CRISPR off-targets	Associated Gene	Spacer sequence (PAM)	Mismatch /Gap	Chromosome (strand)	Start	End
MYC	On-target	*MYC*	GACTGTCCAAAGGGGGTGAAAGG	0	chr8 (+)	127,738,062	127,738,081
	MYC OT1		GAACTGTCCAAAGGGGGTGA-AGG	2	chrX (-)	153,624,558	153,624,577
	MYC OT2	*MYO15A*	CCCTGCCCAAGGGGGGTGAACGG	4	chr17 (+)	18,122,096	18,122,115

CRISPR target	CRISPR off-targets	Associated Gene	Spacer sequence (PAM)	Mismatch /Gap	Chromosome (strand)	Start	End
RAG1	On-target	*RAG1*	GCCTCTTTCCCACCCACCTTGGG	0	chr11 (+)	36,573,311	36,573,330
	RAG1 OT1	*DAZAP1*	ACCCCTTCCCCACCTACCTTGGG	4	chr19 (-)	1,417,538	1,417,557
	RAG1 OT2		TCCTCCTCCCCACCCACCTTCAG	4	chr12 (-)	46,609,202	46,609,221
	RAG1 OT3	*SIN3A*	TCCTCTCTCCCACCCACCTCCGG	3	chr15 (-)	75,454,316	75,454,335

Mismatches/Gaps are listed in red

Supplementary Table 3. Primers for RT-PCR and Intron assay.

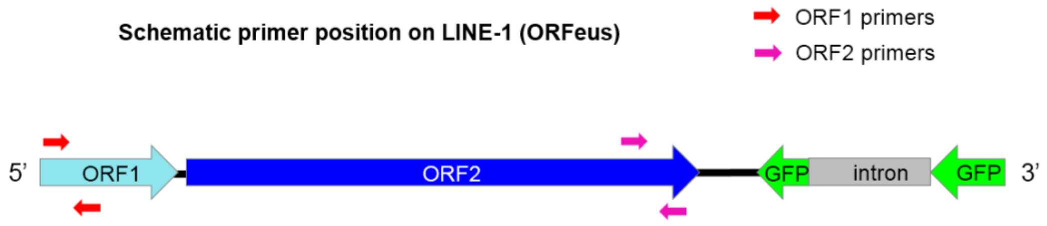

Purpose	Locus	Forward primer	Reverse primer	PCR product length (bp)	Annealing temperature (℃)
RT-PCR	*ORF1*	GAGAACGACTTCGACGAGCTG	ACTTCTCGGTGTTGGTGATG	151	57
	ORF2	CAAGAGCTGCTGCTACAAGGAC	CAGGTGCCCACGAAGCTGATG	189	57
	HPRT	CTGGCGTCGTGATTAGTGATG	GAGCACACAGAGGGCTACAATG	188	57

Purpose	Locus	Forward primer	Reverse primer	PCR product length (bp)	Annealing temperature (℃)
Intron assay	*GFP*	ACGAACTCCAGCAGGACCATG	GCATCGACTTCAAGGAGGACG	1192* / 292#	59

*with intron #without intron

Supplementary Table 4. Primers for amplicon sequencing.

4.1 Forward primers for amplicon sequencing for *MYC* locus

Primer name	sequence
MYC-F Mi0	AATGATACGGCGACCACCGAGATCTACACTCTTTCCCTACACGACGCTCTTCCGATCTGCTGGTTCACTAAGTGCGT
MYC-F Mi1	AATGATACGGCGACCACCGAGATCTACACTCTTTCCCTACACGACGCTCTTCCGATCTTAAGTAGAGGCTGGTTCACTAAGTGCGT
MYC-F Mi2	AATGATACGGCGACCACCGAGATCTACACTCTTTCCCTACACGACGCTCTTCCGATCTCAGCTGGTTCACTAAGTGCGT
MYC-F Mi3	AATGATACGGCGACCACCGAGATCTACACTCTTTCCCTACACGACGCTCTTCCGATCTATACACGATCGCTGGTTCACTAAGTGCGT
MYC-F Mi4	AATGATACGGCGACCACCGAGATCTACACTCTTTCCCTACACGACGCTCTTCCGATCTTGCTGCTGGTTCACTAAGTGCGT
MYC-F Mi5	AATGATACGGCGACCACCGAGATCTACACTCTTTCCCTACACGACGCTCTTCCGATCTGATCGCGCGGTGCTGGTTCACTAAGTGCGT
MYC-F Mi6	AATGATACGGCGACCACCGAGATCTACACTCTTTCCCTACACGACGCTCTTCCGATCTAGATACGCTGGTTCACTAAGTGCGT
MYC-F Mi7	AATGATACGGCGACCACCGAGATCTACACTCTTTCCCTACACGACGCTCTTCCGATCTCGATCATGATCGGCTGGTTCACTAAGTGCGT
MYC-F Mi8	AATGATACGGCGACCACCGAGATCTACACTCTTTCCCTACACGACGCTCTTCCGATCTTTGACTTGGCTGGTTCACTAAGTGCGT
MYC-F Mi9	AATGATACGGCGACCACCGAGATCTACACTCTTTCCCTACACGACGCTCTTCCGATCTTCGATCGTTACCAGCTGGTTCACTAAGTGCGT
MYC-F Mi10	AATGATACGGCGACCACCGAGATCTACACTCTTTCCCTACACGACGCTCTTCCGATCTTAACAATGGGCTGGTTCACTAAGTGCGT
MYC-F Mi11	AATGATACGGCGACCACCGAGATCTACACTCTTTCCCTACACGACGCTCTTCCGATCTATACTGTATCGCTGGTTCACTAAGTGCGT
MYC-F Mi12	AATGATACGGCGACCACCGAGATCTACACTCTTTCCCTACACGACGCTCTTCCGATCTACGTCCTGGCTGGTTCACTAAGTGCGT
MYC-F Mi13	AATGATACGGCGACCACCGAGATCTACACTCTTTCCCTACACGACGCTCTTCCGATCTGTCCGAGGCATGCTGGTTCACTAAGTGCGT
MYC-F Mi14	AATGATACGGCGACCACCGAGATCTACACTCTTTCCCTACACGACGCTCTTCCGATCTCCGTCGCCTGCTGGTTCACTAAGTGCGT
MYC-F Mi15	AATGATACGGCGACCACCGAGATCTACACTCTTTCCCTACACGACGCTCTTCCGATCTGTCAGTACGCGCTGGTTCACTAAGTGCGT

Illumina Miseq adaptor (Forward sequence) Barcode MYC-F

Reverse primers for amplicon sequencing for *MYC* locus

Primer name	sequence
MYC-R Mi0	CAAGCAGAAGACGGCATACGAGATCGGTCTCGGCATTCCTGCTGAACCGCTCTTCCGATCTCTCTCCCCTTGCAGAGCTATCC
MYC-R Mi1	CAAGCAGAAGACGGCATACGAGATCGGTCTCGGCATTCCTGCTGAACCGCTCTTCCGATCTGAGCTCTCCCCTTGCAGAGCTATCC
MYC-R Mi2	CAAGCAGAAGACGGCATACGAGATCGGTCTCGGCATTCCTGCTGAACCGCTCTTCCGATCTTGAGAGCTCTCCCCTTGCAGAGCTATCC
MYC-R Mi3	CAAGCAGAAGACGGCATACGAGATCGGTCTCGGCATTCCTGCTGAACCGCTCTTCCGATCTACGTGAACTCTCCCCTTGCAGAGCTATCC
MYC-R M4	CAAGCAGAAGACGGCATACGAGATCGGTCTCGGCATTCCTGCTGAACCGCTCTTCCGATCTGCTACTCTCCCCTTGCAGAGCTATCC

Illumina Miseq adaptor (Reverse sequence) Barcode MYC-R

4.2 Forward primers for amplicon sequencing for *RAG1* locus

Primer name	sequence
RAG1-F Mi0	AATGATACGGCGACCACCGAGATCTACACTCTTTCCCTACACGACGCTCTTCCGATCTTAAGATACATCAGTGGGATATTG
RAG1-F Mi1	AATGATACGGCGACCACCGAGATCTACACTCTTTCCCTACACGACGCTCTTCCGATCTACGCGCTGCGTAAGATACATCAGTGGGATATTG
RAG1-F Mi2	AATGATACGGCGACCACCGAGATCTACACTCTTTCCCTACACGACGCTCTTCCGATCTCGTATGCTTAAGATACATCAGTGGGATATTG
RAG1-F Mi3	AATGATACGGCGACCACCGAGATCTACACTCTTTCCCTACACGACGCTCTTCCGATCTGTCTAAGATACATCAGTGGGATATTG
RAG1-F Mi4	AATGATACGGCGACCACCGAGATCTACACTCTTTCCCTACACGACGCTCTTCCGATCTTGATTAAGATACATCAGTGGGATATTG
RAG1-F Mi5	AATGATACGGCGACCACCGAGATCTACACTCTTTCCCTACACGACGCTCTTCCGATCTATCACTGCGTAAGATACATCAGTGGGATATTG

Illumina Miseq adaptor (Forward sequence) Barcode RAG1-F

Reverse primers for amplicon sequencing for *RAG1* locus

Primer name	sequence
RAG1-R Mi0	CAAGCAGAAGACGGCATACGAGATCGGTCTCGGCATTCCTGCTGAACCGCTCTTCCGATCTAGGATCTCACCCGGAACAGC
RAG1-R Mi1	CAAGCAGAAGACGGCATACGAGATCGGTCTCGGCATTCCTGCTGAACCGCTCTTCCGATCTTCAGAGGATCTCACCCGGAACAGC
RAG1-R Mi2	CAAGCAGAAGACGGCATACGAGATCGGTCTCGGCATTCCTGCTGAACCGCTCTTCCGATCTGATCGACAGGATCTCACCCGGAACAGC

Illumina Miseq adaptor (Reverse sequence) Barcode RAG1-R

4.3 Forward primers for amplicon sequencing for *CCR5* locus

Primer name	sequence
CCR5-F Mi0	AATGATACGGCGACCACCGAGATCTACACTCTTTCCCTACACGACGCTCTTCCGATCTCCAGGAATCATCTTTACCAGATCT
CCR5-F Mi1	AATGATACGGCGACCACCGAGATCTACACTCTTTCCCTACACGACGCTCTTCCGATCTATGATGTACCAGGAATCATCTTTACCAGATCT
CCR5-F Mi2	AATGATACGGCGACCACCGAGATCTACACTCTTTCCCTACACGACGCTCTTCCGATCTTGCTATCCAGGAATCATCTTTACCAGATCT
CCR5-F Mi3	AATGATACGGCGACCACCGAGATCTACACTCTTTCCCTACACGACGCTCTTCCGATCTGATCCAGGAATCATCTTTACCAGATCT

Illumina Miseq adaptor (Forward sequence) Barcode CCR5-F

Reverse primers for amplicon sequencing for *CCR5* locus

Primer name	sequence
CCR5-R Mi0	CAAGCAGAAGACGGCATACGAGATCGGTCTCGGCATTCCTGCTGAACCGCTCTTCCGATCTCAGGACCAGCCCCAAGATGACTA
CCR5-R Mi1	CAAGCAGAAGACGGCATACGAGATCGGTCTCGGCATTCCTGCTGAACCGCTCTTCCGATCTTGCTCAGGACCAGCCCCAAGATGACTA

Illumina Miseq adaptor (Reverse sequence) Barcode CCR5-R

4.4 Forward primers for amplicon sequencing for *MYC* off-targets #1 (MYC OT1) locus

Primer name	sequence
MYC OT1-F Mi0	AATGATACGGCGACCACCGAGATCTACACTCTTTCCCTACACGACGCTCTTCCGATCTCCTTACTTGAAGCCAGAGGTC
MYC OT1-F Mi1	AATGATACGGCGACCACCGAGATCTACACTCTTTCCCTACACGACGCTCTTCCGATCTGTAGCTCCTTACTTGAAGCCAGAGGTC
MYC OT1-F Mi2	AATGATACGGCGACCACCGAGATCTACACTCTTTCCCTACACGACGCTCTTCCGATCTTAGCTAGACCCTTACTTGAAGCCAGAGGTC
MYC OT1-F Mi3	AATGATACGGCGACCACCGAGATCTACACTCTTTCCCTACACGACGCTCTTCCGATCTAGCAGCCTTACTTGAAGCCAGAGGTC

Illumina Miseq adaptor (Forward sequence) Barcode MYC OT1-F

Reverse primers for amplicon sequencing for *MYC* off-targets #1 (MYC OT1) locus

Primer name	sequence
MYC OT1-R Mi0	CAAGCAGAAGACGGCATACGAGATCGGTCTCGGCATTCCTGCTGAACCGCTCTTCCGATCTCACAGGACCGTGCCTCCTAGC
MYC OT1-R Mi1	CAAGCAGAAGACGGCATACGAGATCGGTCTCGGCATTCCTGCTGAACCGCTCTTCCGATCTTGACATGCACAGGACCGTGCCTCCTAGC
MYC OT1-R Mi2	CAAGCAGAAGACGGCATACGAGATCGGTCTCGGCATTCCTGCTGAACCGCTCTTCCGATCTGCTGCACAGGACCGTGCCTCCTAGC

Illumina Miseq adaptor (Reverse sequence) Barcode MYC OT1-R

4.5 Forward primers for amplicon sequencing for *FANCF* locus

Primer name	sequence
FANCF-F Mi0	AATGATACGGCGACCACCGAGATCTACACTCTTTCCCTACACGACGCTCTTCCGATCTAGAGTCGCCGTCTCCAAGGTG
FANCF-F Mi1	AATGATACGGCGACCACCGAGATCTACACTCTTTCCCTACACGACGCTCTTCCGATCTACGTCACTTAGAGTCGCCGTCTCCAAGGTG
FANCF-F Mi2	AATGATACGGCGACCACCGAGATCTACACTCTTTCCCTACACGACGCTCTTCCGATCTCTCACTAAGAGTCGCCGTCTCCAAGGTG
FANCF-F Mi3	AATGATACGGCGACCACCGAGATCTACACTCTTTCCCTACACGACGCTCTTCCGATCTGCAGAGTCGCCGTCTCCAAGGTG
FANCF-F Mi4	AATGATACGGCGACCACCGAGATCTACACTCTTTCCCTACACGACGCTCTTCCGATCTTGTCAAGAGTCGCCGTCTCCAAGGTG
FANCF-F Mi5	AATGATACGGCGACCACCGAGATCTACACTCTTTCCCTACACGACGCTCTTCCGATCTCACAGCAGAGTCGCCGTCTCCAAGGTG

Illumina Miseq adaptor (Forward sequence) Barcode FANCF-F

Reverse primers for amplicon sequencing for *FANCF* locus

Primer name	sequence
FANCF-R Mi0	CAAGCAGAAGACGGCATACGAGATCGGTCTCGGCATTCCTGCTGAACCGCTCTTCCGATCTGATGGATGTGGCGCAGGTAG
FANCF-R Mi1	CAAGCAGAAGACGGCATACGAGATCGGTCTCGGCATTCCTGCTGAACCGCTCTTCCGATCTACGCACGCCGATGGATGTGGCGCAGGTAG
FANCF-R Mi2	CAAGCAGAAGACGGCATACGAGATCGGTCTCGGCATTCCTGCTGAACCGCTCTTCCGATCTCGCATGCAGATGGATGTGGCGCAGGTAG
FANCF-R Mi3	CAAGCAGAAGACGGCATACGAGATCGGTCTCGGCATTCCTGCTGAACCGCTCTTCCGATCTGTCGATGGATGTGGCGCAGGTAG
FANCF-R Mi4	CAAGCAGAAGACGGCATACGAGATCGGTCTCGGCATTCCTGCTGAACCGCTCTTCCGATCTTGATGATGGATGTGGCGCAGGTAG
FANCF-R Mi5	CAAGCAGAAGACGGCATACGAGATCGGTCTCGGCATTCCTGCTGAACCGCTCTTCCGATCTCATACGATGGATGTGGCGCAGGTAG

Illumina Miseq adaptor (Reverse sequence) Barcode FANCF-R

4.6 Forward primers for amplicon sequencing for *BCL11A* enhancer locus

Primer name	sequence
BCL11A-F Mi0	AATGATACGGCGACCACCGAGATCTACACTCTTTCCCTACACGACGCTCTTCCGATCTTCTTAGACATAACACACCAGG
BCL11A-F Mi1	AATGATACGGCGACCACCGAGATCTACACTCTTTCCCTACACGACGCTCTTCCGATCTCAGCTCTTAGACATAACACACCAGG

Illumina Miseq adaptor (Forward sequence) Barcode BCL11A-F

Reverse primers for amplicon sequencing for *BCL11A* enhancer locus

Primer name	sequence
BCL11A-R Mi0	CAAGCAGAAGACGGCATACGAGATCGGTCTCGGCATTCCTGCTGAACCGCTCTTCCGATCTAGAGGTCTGCCAGTCCTCTTC
BCL11A-R Mi1	CAAGCAGAAGACGGCATACGAGATCGGTCTCGGCATTCCTGCTGAACCGCTCTTCCGATCTTACTCAGAGGTCTGCCAGTCCTCTTC
BCL11A-R Mi2	CAAGCAGAAGACGGCATACGAGATCGGTCTCGGCATTCCTGCTGAACCGCTCTTCCGATCTCTGCAGAGGTCTGCCAGTCCTCTTC

Illumina Miseq adaptor (Reverse sequence) Barcode BCL11A-R

4.7 Forward primers for amplicon sequencing for *HBG1/2* promoter locus

Primer name	sequence
HBG1/2-F Mi0	AATGATACGGCGACCACCGAGATCTACACTCTTTCCCTACACGACGCTCTTCCGATCTTGCCTTGTCAAGGCTATTGGTC
HBG1/2-F Mi1	AATGATACGGCGACCACCGAGATCTACACTCTTTCCCTACACGACGCTCTTCCGATCTATGACGTGCCTTGTCAAGGCTATTGGTC

Illumina Miseq adaptor (Forward sequence) Barcode HBG1/2-F

Reverse primers for amplicon sequencing for *HBG1/2* promoter locus

Primer name	sequence
HBG1/2-R Mi0	CAAGCAGAAGACGGCATACGAGATCGGTCTCGGCATTCCTGCTGAACCGCTCTTCCGATCTTGGAATGACTGAATCGGAAC
HBG1/2-R Mi1	CAAGCAGAAGACGGCATACGAGATCGGTCTCGGCATTCCTGCTGAACCGCTCTTCCGATCTACTGTCTGGAATGACTGAATCGGAAC
HBG1/2-R Mi2	CAAGCAGAAGACGGCATACGAGATCGGTCTCGGCATTCCTGCTGAACCGCTCTTCCGATCTGACTGGAATGACTGAATCGGAAC

Illumina Miseq adaptor (Reverse sequence) Barcode HBG1/2-R

Supplementary Table 5. Primers for PolyA-seq.

5.1 PolyA-seq primers

Primer name	Sequences
Biotin-LEAP	/5biosg/GTTCGAAATCGATAAGCTTGGATCC (1st round primer for PolyA-seq)
SV40-polyA-F	GCAATAAACAAGTTAACAACAAAAAAAAA (2nd round primer for PolyA-seq)
SV40-polyA-F Mi0	AATGATACGGCGACCACCGAGATCTACACTCTTTCCCTACACGACGCTCTTCCGATCTGCAATAAACAAGTTAACAACAAAAAAAAA
SV40-polyA-F Mi1	AATGATACGGCGACCACCGAGATCTACACTCTTTCCCTACACGACGCTCTTCCGATCTATGCAATAAACAAGTTAACAACAAAAAAAAA
SV40-polyA-F Mi2	AATGATACGGCGACCACCGAGATCTACACTCTTTCCCTACACGACGCTCTTCCGATCTTGCGCAATAAACAAGTTAACAACAAAAAAAAA
SV40-polyA-F Mi3	AATGATACGGCGACCACCGAGATCTACACTCTTTCCCTACACGACGCTCTTCCGATCTCAGTGCAATAAACAAGTTAACAACAAAAAAAAA
SV40-polyA-F Mi4	AATGATACGGCGACCACCGAGATCTACACTCTTTCCCTACACGACGCTCTTCCGATCTACTACGCAATAAACAAGTTAACAACAAAAAAAAA

Illumina Miseq adaptor (Forward sequence) Barcode SV40-polyA-F

5.2 Linker sequence

Name	Sequence	Purpose
HaeIII upper linker	GTAATACGACTCACTATAGGGCACGCGTGGTCGACGGCCCGGGCTGGTTAT	The upper strand of ligated linker
HaeIII lower linker	TAACCAGCCC (5'P, 3'inverted dT)	The lower strand of ligated linker
AP1	GTAATACGACTCACTATAGGGC	1st round linker primer
AP2	ACTATAGGGCACGCGTGGTC	2nd round linker primer

5.3 Reverse barcoding primers for Illumina Miseq sequencing

Primer name	Sequence
AP2-PE7 Mi0	CAAGCAGAAGACGGCATACGAGATCGGTCTCGGCATTCCTGCTGAACCGCTCTTCCGATCTACTATAGGGCACGCGTGGT
AP2-PE7 Mi1	CAAGCAGAAGACGGCATACGAGATCGGTCTCGGCATTCCTGCTGAACCGCTCTTCCGATCTTGCGATACTATAGGGCACGCGTGGT
AP2-PE7 Mi2	CAAGCAGAAGACGGCATACGAGATCGGTCTCGGCATTCCTGCTGAACCGCTCTTCCGATCTGAGTCGCTAGACTATAGGGCACGCGTGGT
AP2-PE7 Mi3	CAAGCAGAAGACGGCATACGAGATCGGTCTCGGCATTCCTGCTGAACCGCTCTTCCGATCTCTACGCTACTATAGGGCACGCGTGGT
AP2-PE7 Mi4	CAAGCAGAAGACGGCATACGAGATCGGTCTCGGCATTCCTGCTGAACCGCTCTTCCGATCTATGCACTATAGGGCACGCGTGGT
AP2-PE7 Mi5	CAAGCAGAAGACGGCATACGAGATCGGTCTCGGCATTCCTGCTGAACCGCTCTTCCGATCTGCACTATAGGGCACGCGTGGT
AP2-PE7 Mi6	CAAGCAGAAGACGGCATACGAGATCGGTCTCGGCATTCCTGCTGAACCGCTCTTCCGATCTTGCTGACTATAGGGCACGCGTGGT
AP2-PE7 Mi7	CAAGCAGAAGACGGCATACGAGATCGGTCTCGGCATTCCTGCTGAACCGCTCTTCCGATCTCATACTATAGGGCACGCGTGGT

Illumina Miseq adaptor (Reverse sequence) Barcode AP2 sequence

Supplementary Sequences

Supplementary Sequence 1. DNA sequences of pCEP4 GFP control, pCEP4 L1-ORFeus, pCEP4 L1-ORFeus-ENm (H230A) (L1-ENm), and pCEP4 L1-ORFeus-RTm (D702Y) (L1-RTm) plasmids used in this study.

Supplementary Sequence 2. DNA sequences of endogenous human L1 (L1Hs and L1RP)

Supplementary Sequence 3. DNA sequences of pU6-pegRNAs, lentiCRISPR v2 plasmid, pCMV-PE2 plasmid, pCMV-BEs (BE2/BE3/BE4-Gam/AncBE4max/ABE8e) plasmids used for insertion sources analysis.

Supplementary Sequence 4. Example L1-ORFeus insertional sequences obtained by amplicon sequencing at the PE3 (MYC +2-4AAA del) editing site in HEK293T cells expressing L1-ORFeus.

Supplementary Sequence 5. L1-ORFeus insertions bridging the intron of GFP obtained by amplicon sequencing at the PE3 (MYC +2-4AAA del) editing site in HEK293T cells expressing L1-ORFeus.

Supplementary Sequence 6. L1-ORFeus insertions bridging the intron of GFP obtained by amplicon sequencing at the MYC CRISPR/Cas9 target site in HEK293T cells expressing L1-ORFeus.

Supplementary Sequence 7. L1-ORFeus insertions bridging the intron of GFP obtained by amplicon sequencing at the RAG1 CRISPR/Cas9 target site in HEK293T cells expressing L1-ORFeus.

Supplementary Sequence 8. L1-ORFeus insertion bridging the intron of GFP obtained by amplicon sequencing at the CCR5 CRISPR/Cas9 target site in HEK293T cells expressing L1-ORFeus.

Supplementary Sequence 9. L1-ORFeus insertions bridging the intron of GFP obtained by amplicon sequencing at the MYC CRISPR/Cas9 target site in HeLa cells expressing L1-ORFeus.

Supplementary Sequence 10. L1-ORFeus insertional sequence obtained by amplicon sequencing at the CCR5 CRISPR/Cas9 target site in HEK293T cells expressing L1-RTm.

Supplementary Sequence 11. Example sequences of PolyA-seq from MYC CRISPR editing in HEK293T cells expressing L1-ORFeus.

Supplementary Sequence 12. Example L1Hs sequences obtained by amplicon sequencing at the MYC/RAG1 CRISPR/Cas9 target site in HEK293T cells.

Supplementary Sequence 13. DNA sequences of pCEP4 L1RP, pCEP4 L1RP-ENm (H230A) (L1RP-ENm), and pCEP4 L1RP-RTm (D702Y) (L1RP-RTm) plasmids used in this study.

Supplementary Sequence 1. DNA sequences of pCEP4 GFP control, pCEP4 L1-ORFeus, pCEP4 L1-ORFeus-ENm (H230A) (L1-ENm), and pCEP4 L1-ORFeus-RTm (D702Y) (L1-RTm) plasmids used in this study.

1.1 pCEP4 GFP control vector (1-10646bp)

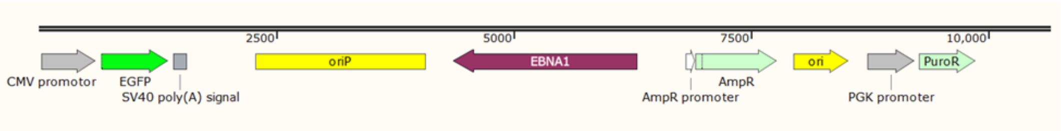

Features	Regions (bp)
CMV promoter	30-605
**EGFP**	**652-1368**
SV40 poly(A) signal	1415-1549
EBV-oriP	2278-4067
EBNA1	4369-6294
AmpR	6920-7780
pUC origin	7951-8539
PuroR	9273-9872

GTTGACATTGATTATTGACTAGTTATTAATAGTAATCAATTACGGGGTCATTAGTTCATAGCCCATATATGGAGTTCCGCGTTACATAACTTACGGT
AAATGGCCCGCCTGGCTGACCGCCCAACGACCCCCGCCCATTGACGTCAATAATGACGTATGTTCCCATAGTAACGCCAATAGGGACTTTCC
ATTGACGTCAATGGGTGGAGTATTTACGGTAAACTGCCCACTTGGCAGTACATCAAGTGTATCATATGCCAAGTCCGCCCCCTATTGACGTCAA
TGACGGTAAATGGCCCGCCTGGCATTATGCCCAGTACATGACCTTACGGGACTTTCCTACTTGGCAGTACATCTACGTATTAGTCATCGCTATT
ACCATGGTGATGCGGTTTTGGCAGTACACCAATGGGCGTGGATAGCGGTTTGACTCACGGGGATTTCCAAGTCTCCACCCCATTGACGTCAA
TGGGAGTTTGTTTTGGCACCAAAATCAACGGGACTTTCCAAAATGTCGTAATAACCCCGCCCCGTTGACGCAAATGGGCGGTAGGCGTGTAC
GGTGGGAGGTCTATATAAGCAGAGCTCGTTTAGTGAACCGTCAGATCTCTAGAAGCTGGGTACCAGCTGCTAGCGCTACCGGTCGCCACC**AT**
GGTGAGCAAGGGCGAGGAGCTGTTCACCGGGGTGGTGCCCATCCTGGTCGAGCTGGACGGCGACGTAAACGGCCACAAGTTCAGCGTG
TCCGGCGAGGGCGAGGGCGATGCCACCTACGGCAAGCTGACCCTGAAGTTCATCTGCACCACCGGCAAGCTGCCCGTGCCCTGGCCCA
CCCTCGTGACCACCCTGACCTACGGCGTGCAGTGCTTCAGCCGCTACCCCGACCACATGAAGCAGCACGACTTCTTCAAGTCCGCCATG
CCCGAAGGCTACGTCCAGGAGCGCACCATCTTCTTCAAGGACGACGGCAACTACAAGACCCGCGCCGAGGTGAAGTTCGAGGGCGACA
CCCTGGTGAACCGCATCGAGCTGAAGGGCATCGACTTCAAGGAGGACGGCAACATCCTGGGGCACAAGCTGGAGTACAACTACAACAG
CCACAACGTCTATATCATGGCCGACAAGCAGAAGAACGGCATCAAGGTGAACTTCAAGATCCGCCACAACATCGAGGACGGCAGCGTGC
AGCTCGCCGACCACTACCAGCAGAACACCCCCATCGGCGACGGCCCCGTGCTGCTGCCCGACAACCACTACCTGAGCACCCAGTCCGC
CCTGAGCAAAGACCCCAACGAGAAGCGCGATCACATGGTCCTGCTGGAGTTCGTGACCGCCGCCGGGATCACTCTCGGCATGGACGAG
CTGTACAAGTCCGGACTCAGATCTCGAGCTCAAGCTTCGAATAGCGGCCGCTAAGGATCCAGACATGATAAGATACATTGATGAGTTTGGACA
AACCACAACTAGAATGCAGTGAAAAAAATGCTTTATTTGTGAAATTTGTGATGCTATTGCTTTATTTGTAACCATTATAAGCTGCAATAAACAAGTT
AACAACAACAATTGCATTCATTTTATGTTTCAGGTTCAGGGGGAGGTGTGGGAGGTTTTTTAAAGCAAGTAAAACCTCTACAAATGTGGTATGG
CTGATTATGATCCGGCTGCCTCGCGCGTTTCGGTGATGACGGTGAAAACCTCTGACACATGCAGCTCCCGGAGACGGTCACAGCTTGTCTG
TAAGCGGATGCCGGGAGCAGACAAGCCCGTCAGGGCGCGTCAGCGGGTGTTGGCGGGTGTCGGGGCGCAGCCATGAGGTCGATCGACTC
TAGAGGATCGATCCCCGCCCCGGACGAACTAAACCTGACTACGACATCTCTGCCCCTTCTTCGCGGGGCAGTGCATGTAATCCCTTCAGTTG
GTTGGTACAACTTGCCAACTGGGCCCTGTTCCACATGTGACACGGGGGGGGACCAAACACAAAGGGGTTCTCTGACTGTAGTTGACATCCT
TATAAATGGATGTGCACATTTGCCAACACTGAGTGGCTTTCATCCTGGAGCAGACTTTGCAGTCTGTGGACTGCAACACAACATTGCCTTTATG
TGTAACTCTTGGCTGAAGCTCTTACACCAATGCTGGGGGACATGTACCTCCCAGGGGCCCAGGAAGACTACGGGAGGCTACACCAACGTCA
ATCAGAGGGGCCTGTGTAGCTACCGATAAGCGGACCCTCAAGAGGGCATTAGCAATAGTGTTTATAAGGCCCCCTTGTTAACCCTAAACGGGT
AGCATATGCTTCCCGGGTAGTAGTATATACTATCCAGACTAACCCTAATTCAATAGCATATGTTACCCAACGGGAAGCATATGCTATCGAATTAGG
GTTAGTAAAAGGGTCCTAAGGAACAGCGATATCTCCCACCCCATGAGCTGTCACGGTTTTATTTACATGGGGTCAGGATTCCACGAGGGTAGT
GAACCATTTTAGTCACAAGGGCAGTGGCTGAAGATCAAGGAGCGGGCAGTGAACTCTCCTGAATCTTCGCCTGCTTCTTCATTCTCCTTCGT
TTAGCTAATAGAATAACTGCTGAGTTGTGAACAGTAAGGTGTATGTGAGGTGCTCGAAAACAAGGTTTCAGGTGACGCCCCCAGAATAAAATTT
GGACGGGGGGTTCAGTGGTGGCATTGTGCTATGACACCAATATAACCCTCACAAACCCCTTGGGCAATAAATACTAGTGTAGGAATGAAACAT
TCTGAATATCTTTAACAATAGAAATCCATGGGGTGGGGACAAGCCGTAAAGACTGGATGTCCATCTCACACGAATTTATGGCTATGGGCAACAC
ATAATCCTAGTGCAATATGATACTGGGGTTATTAAGATGTGTCCCAGGCAGGGACCAAGACAGGTGAACCATGTTGTTACACTCTATTTGTAACA
AGGGGAAAGAGAGTGGACGCCGACAGCAGCGGACTCCACTGGTTGTCTCTAACACCCCCGAAAATTAAACGGGGCTCCACGCCAATGGGG
CCCATAAACAAAGACAAGTGGCCACTCTTTTTTTTGAAATTGTGGAGTGGGGGCACGCGTCAGCCCCCACACGCCGCCCTGCGGTTTTGGA
CTGTAAAATAAGGGTGTAATAACTTGGCTGATTGTAACCCCGCTAACCACTGCGGTCAAACCACTTGCCCACAAAACCACTAATGGCACCCCG
GGGAATACCTGCATAAGTAGGTGGGCGGGCCAAGATAGGGGCGCGATTGCTGCGATCTGGAGGACAAATTACACACACTTGCGCCTGAGCG
CCAAGCACAGGGTTGTTGGTCCTCATATTCACGAGGTCGCTGAGAGCACGGTGGGCTAATGTTGCCATGGGTAGCATATACTACCCAAATATC
TGGATAGCATATGCTATCCTAATCTATATCTGGGTAGCATAGGCTATCCTAATCTATATCTGGGTAGCATATGCTATCCTAATCTATATCTGGGTAGT
ATATGCTATCCTAATTTATATCTGGGTAGCATAGGCTATCCTAATCTATATCTGGGTAGCATATGCTATCCTAATCTATATCTGGGTAGTATATGCTAT
CCTAATCTGTATCCGGGTAGCATATGCTATCCTAATAGAGATTAGGGTAGTATATGCTATCCTAATTTATATCTGGGTAGCATATACTACCCAAATAT
CTGGATAGCATATGCTATCCTAATCTATATCTGGGTAGCATATGCTATCCTAATCTATATCTGGGTAGCATAGGCTATCCTAATCTATATCTGGGTAG
CATATGCTATCCTAATCTATATCTGGGTAGTATATGCTATCCTAATTTATATCTGGGTAGCATAGGCTATCCTAATCTATATCTGGGTAGCATATGCTA
TCCTAATCTATATCTGGGTAGTATATGCTATCCTAATCTGTATCCGGGTAGCATATGCTATCCTCATGCATATACAGTCAGCATATGATACCCAGTA
GTAGAGTGGGAGTGCTATCCTTTGCATATGCCGCCACCTCCCAAGGGGGCGTGAATTTTCGCTGCTTGTCCTTTTCCTGCTGGTTGCTCCCA
TTCTTAGGTGAATTTAAGGAGGCCAGGCTAAAGCCGTCGCATGTCTGATTGCTCACCAGGTAAATGTCGCTAATGTTTTCCAACGCGAGAAGG
TGTTGAGCGCGGAGCTGAGTGACGTGACAACATGGGTATGCCCAATTGCCCCATGTTGGGAGGACGAAAATGGTGACAAGACAGATGGCCA

GAAATACACCAACAGCACGCATGATGTCTACTGGGGATTTATTCTTTAGTGCGGGGGAATACACGGCTTTTAATACGATTGAGGGCGTCTCCTA
ACAAGTTACATCACTCCTGCCCTTCCTCACCCTCATCTCCATCACCTCCTTCATCTCCGTCATCTCCGTCATCACCCTCCGCGGCAGCCCCTT
CCACCATAGGTGGAAACCAGGGAGGCAAATCTACTCCATCGTCAAAGCTGCACACAGTCACCCTGATATTGCAGGTAGGAGCGGGCTTTGTC
ATAACAAGGTCCTTAATCGCATCCTTCAAAACCTCAGCAAATATATGAGTTTGTAAAAAGACCATGAAATAACAGACAATGGACTCCCTTAGCGG
GCCAGGTTGTGGGCCGGGTCCAGGGGCCATTCCAAAGGGGAGACGACTCAATGGTGTAAGACGACATTGTGGAATAGCAAGGGCAGTTCC
TCGCCTTAGGTTGTAAAGGGAGGTCTTACTACCTCCATATACGAACACACCGGCGACCCAAGTTCCTTCGTCGGTAGTCCTTTCTACGTGACT
CCTAGCCAGGAGAGCTCTTAAACCTTCTGCAATGTTCTCAAATTTCGGGTTGGAACCTCCTTGACCACGATGCTTTCCAAACCACCCTCCTTT
TTTGCGCCTGCCTCCATCACCCTGACCCCGGGGTCCAGTGCTTGGGCCTTCTCCTGGGTCATCTGCGGGGCCCTGCTCTATCGCTCCCGG
GGGCACGTCAGGCTCACCATCTGGGCCACCTTCTTGGTGGTATTCAAAATAATCGGCTTCCCCTACAGGGTGGAAAAATGGCCTTCTACCTG
GAGGGGGCCTGCGCGGTGGAGACCCGGATGATGATGACTGACTACTGGGACTCCTGGGCCTCTTTTCTCCACGTCCACGACCTCTCCCCC
TGGCTCTTTCACGACTTCCCCCCCTGGCTCTTTCACGTCCTCTACCCCGGCGGCCTCCACTACCTCCTCGACCCCGGCCTCCACTACCTCCT
CGACCCCGGCCTCCACTGCCTCCTCGACCCCGGCCTCCACCTCCTGCTCCTGCCCCTCCTGCTCCTGCCCCTCCTCCTGCTCCTGCCCCT
CCTGCCCCTCCTGCTCCTGCCCCTCCTGCCCCTCCTGCTCCTGCCCCTCCTGCCCCTCCTGCTCCTGCCCCTCCTGCCCCTCCTCCTGCTC
CTGCCCCTCCTGCCCCTCCTCCTGCTCCTGCCCCTCCTGCCCCTCCTGCTCCTGCCCCTCCTGCCCCTCCTGCTCCTGCCCCTCCTGCCCC
TCCTGCTCCTGCCCCTCCTGCTCCTGCCCCTCCTGCTCCTGCCCCTCCTGCTCCTGCCCCTCCTGCCCCTCCTGCCCCTCCTCCTGCTCCT
GCCCCTCCTGCTCCTGCCCCTCCTGCCCCTCCTGCCCCTCCTGCTCCTGCCCCTCCTCCTGCTCCTGCCCCTCCTGCCCCTCCTGCCCCTC
CTCCTGCTCCTGCCCCTCCTGCCCCTCCTCCTGCTCCTGCCCCTCCTCCTGCTCCTGCCCCTCCTGCCCCTCCTGCCCCTCCTCCTGCTCC
TGCCCCTCCTGCCCCTCCTCCTGCTCCTGCCCCTCCTCCTGCTCCTGCCCCTCCTGCCCCTCCTGCCCCTCCTCCTGCTCCTGCCCCTCCT
CCTGCTCCTGCCCCTCCTGCCCCTCCTGCCCCTCCTGCCCCTCCTCCTGCTCCTGCCCCTCCTCCTGCTCCTGCCCCTCCTGCTCCTGCCC
CTCCCGCTCCTGCTCCTGCTCCTGTTCCACCGTGGGTCCCTTTGCAGCCAATGCAACTTGGACGTTTTTGGGGTCTCCGGACACCATCTCTA
TGTCTTGGCCCTGATCCTGAGCCGCCCGGGGCTCCTGGTCTTCCGCCTCCTCGTCCTCGTCCTCTTCCCCGTCCTCGTCCATGGTTATCAC
CCCCTCTTCTTTGAGGTCCACTGCCGCCGGAGCCTTCTGGTCCAGATGTGTCTCCCTTCTCTCCTAGGCCATTTCCAGGTCCTGTACCTGGC
CCCTCGTCAGACATGATTCACACTAAAAGAGATCAATAGACATCTTTATTAGACGACGCTCAGTGAATACAGGGAGTGCAGACTCCTGCCCCC
TCCAACAGCCCCCCCACCCTCATCCCCTTCATGGTCGCTGTCAGACAGATCCAGGTCTGAAAATTCCCCATCCTCCGAACCATCCTCGTCCT
CATCACCAATTACTCGCAGCCCGGAAAACTCCCGCTGAACATCCTCAAGATTTGCGTCCTGAGCCTCAAGCCAGGCCTCAAATTCCTCGTCC
CCCTTTTTGCTGGACGGTAGGGATGGGGATTCTCGGGACCCCTCCTCTTCCTCTTCAAGGTCACCAGACAGAGATGCTACTGGGGCAACGG
AAGAAAAGCTGGGTGCGGCCTGTGAGGATCAGCTTATCGATGATAAGCTGTCAAACATGAGAATTCTTGAAGACGAAAGGGCCTCGTGATAC
GCCTATTTTTATAGGTTAATGTCATGATAATAATGGTTTCTTAGACGTCAGGTGGCACTTTTCGGGGAAATGTGCGCGGAACCCCTATTTGTTTA
TTTTTCTAAATACATTCAAATATGTATCCGCTCATGAGACAATAACCCTGATAAATGCTTCAATAATATTGAAAAAGGAAGAGTATGAGTATTCAAC
ATTTCCGTGTCGCCCTTATTCCCTTTTTTGCGGCATTTTGCCTTCCTGTTTTTGCTCACCCAGAAACGCTGGTGAAAGTAAAAGATGCTGAAGA
TCAGTTGGGTGCACGAGTGGGTTACATCGAACTGGATCTCAACAGCGGTAAGATCCTTGAGAGTTTTCGCCCCGAAGAACGTTTTCCAATGA
TGAGCACTTTTAAAGTTCTGCTATGTGGCGCGGTATTATCCCGTGTTGACGCCGGGCAAGAGCAACTCGGTCGCCGCATACACTATTCTCAGA
ATGACTTGGTTGAGTACTCACCAGTCACAGAAAAGCATCTTACGGATGGCATGACAGTAAGAGAATTATGCAGTGCTGCCATAACCATGAGTG
ATAACACTGCGGCCAACTTACTTCTGACAACGATCGGAGGACCGAAGGAGCTAACCGCTTTTTTGCACAACATGGGGGATCATGTAACTCGC
CTTGATCGTTGGGAACCGGAGCTGAATGAAGCCATACCAAACGACGAGCGTGACACCACGATGCCTGCAGCAATGGCAACAACGTTGCGCA
AACTATTAACTGGCGAACTACTTACTCTAGCTTCCCGGCAACAATTAATAGACTGGATGGAGGCGGATAAAGTTGCAGGACCACTTCTGCGCT
CGGCCCTTCCGGCTGGCTGGTTTATTGCTGATAAATCTGGAGCCGGTGAGCGTGGGTCTCGCGGTATCATTGCAGCACTGGGGCCAGATGG
TAAGCCCTCCCGTATCGTAGTTATCTACACGACGGGGAGTCAGGCAACTATGGATGAACGAAATAGACAGATCGCTGAGATAGGTGCCTCACT
GATTAAGCATTGGTAACTGTCAGACCAAGTTTACTCATATATACTTTAGATTGATTTAAAACTTCATTTTTAATTTAAAAGGATCTAGGTGAAGATC
CTTTTTGATAATCTCATGACCAAAATCCCTTAACGTGAGTTTTCGTTCCACTGAGCGTCAGACCCCGTAGAAAAGATCAAAGGATCTTCTTGAG
ATCCTTTTTTTCTGCGCGTAATCTGCTGCTTGCAAACAAAAAAACCACCGCTACCAGCGGTGGTTTGTTTGCCGGATCAAGAGCTACCAACTC
TTTTTCCGAAGGTAACTGGCTTCAGCAGAGCGCAGATACCAAATACTGTCCTTCTAGTGTAGCCGTAGTTAGGCCACCACTTCAAGAACTCTG
TAGCACCGCCTACATACCTCGCTCTGCTAATCCTGTTACCAGTGGCTGCTGCCAGTGGCGATAAGTCGTGTCTTACCGGGTTGGACTCAAGA
CGATAGTTACCGGATAAGGCGCAGCGGTCGGGCTGAACGGGGGGTTCGTGCACACAGCCCAGCTTGGAGCGAACGACCTACACCGAACTG
AGATACCTACAGCGTGAGCTATGAGAAAGCGCCACGCTTCCCGAAGGGAGAAAGGCGGACAGGTATCCGGTAAGCGGCAGGGTCGGAACA
GGAGAGCGCACGAGGGAGCTTCCAGGGGGAAACGCCTGGTATCTTTATAGTCCTGTCGGGTTTCGCCACCTCTGACTTGAGCGTCGATTTT
TGTGATGCTCGTCAGGGGGGCGGAGCCTATGGAAAAACGCCAGCAACGCGGCCTTTTTACGGTTCCTGGCCTTTTGCTGGCCTTGAAGCTG
TCCCTGATGGTCGTCATCTACCTGCCTGGACAGCATGGCCTGCAACGCGGGCATCCCGATGCCGCCGGAAGCGAGAAGAATCATAATGGGG
AAGGCCATCCAGCCTCGCGTCGAATTCGTCGACCTCGAAATTCTACCGGGTAGGGGAGGCGCTTTTCCCAAGGCAGTCTGGAGCATGCGCT
TTAGCAGCCCCGCTGGGCACTTGGCGCTACACAAGTGGCCTCTGGCCTCGCACACATTCCACATCCACCGGTAGGCGCCAACCGGCTCCG
TTCTTTGGTGGCCCCTTCGCGCCACCTTCTACTCCTCCCCTAGTCAGGAAGTTCCCCCCCGCCCCGCAGCTCGCGTCGTGCAGGACGTGAC
AAATGGAAGTAGCACGTCTCACTAGTCTCGTGCAGATGGACAGCACCGCTGAGCAATGGAAGCGGGTAGGCCTTTGGGGCAGCGGCCAATA
GCAGCTTTGCTCCTTCGCTTTCTGGGCTCAGAGGCTGGGAAGGGGTGGGTCCGGGGGCGGGCTCAGGGGCGGGCTCAGGGGCGGGGCG
GGCGCCCGAAGGTCCTCCGGAGGCCCGGCATTCTGCACGCTTCAAAAGCGCACGTCTGCCGCGCTGTTCTCCTCTTCCTCATCTCCGGGC
CTTTCGACCTGCATCCATCTAGATCTCGAGCAGCTGAAGCTTACCATGACCGAGTACAAGCCCACGGTGCGCCTCGCCACCCGCGACGACG
TCCCCAGGGCCGTACGCACCCTCGCCGCCGCGTTCGCCGACTACCCCGCCACGCGCCACACCGTCGATCCGGACCGCCACATCGAGCGG
GTCACCGAGCTGCAAGAACTCTTCCTCACGCGCGTCGGGCTCGACATCGGCAAGGTGTGGGTCGCGGACGACGGCGCCGCGGTGGCGGT
CTGGACCACGCCGGAGAGCGTCGAAGCGGGGGCGGTGTTCGCCGAGATCGGCCCGCGCATGGCCGAGTTGAGCGGTTCCCGGCTGGCC
GCGCAGCAACAGATGGAAGGCCTCCTGGCGCCGCACCGGCCCAAGGAGCCCGCGTGGTTCCTGGCCACCGTCGGCGTCTCGCCCGACC
ACCAGGGCAAGGGTCTGGGCAGCGCCGTCGTGCTCCCCGGAGTGGAGGCGGCCGAGCGCGCCGGGGTGCCCGCCTTCCTGGAGACCTC
CGCGCCCCGCAACCTCCCCTTCTACGAGCGGCTCGGCTTCACCGTCACCGCCGACGTCGAGGTGCCCGAAGGACCGCGCACCTGGTGCA
TGACCCGCAAGCCCGGTGCCTGACGCCCGCCCCACGACCCGCAGCGCCCGACCGAAAGGAGCGCACGACCCCATGCATCGATGATATCAG
ATCCCCGGGATGCAGAAATTGATGATCTATTAAACAATAAAGATGTCCACTAAAATGGAAGTTTTTCCTGTCATACTTTGTTAAGAAGGGTGAGA
ACAGAGTACCTACATTTTGAATGGAAGGATTGGAGCTACGGGGGTGGGGGTGGGGTGGGATTAGATAAATGCCTGCTCTTTACTGAAGGCTC
TTTACTATTGCTTTATGATAATGTTTCATAGTTGGATATCATAATTTAAACAAGCAAAACCAAATTAAGGGCCAGCTCATTCCTCCCACTCATGATC
TATAGATCTATAGATCTCTCGTGGGATCATTGTTTTTCTCTTGATTCCCACTTTGTGGTTCTAAGTACTGTGGTTTCCAAATGTGTCAGTTTCATA
GCCTGAAGAACGAGATCAGCAGCCTCTGTTCCACATACACTTCATTCTCAGTATTGTTTTGCCAAGTTCTAATTCCATCAGAAGCTGGTCGACC
AATTCTCATGTTTGACAGCTTATCATCGCAGATCCGGGCAACGTTGTTGCCATTGCTGCAGGCGCAGAACTGGTAGGTATGGAAGATCTATAC
ATTGAATCAATATTGGCAATTAGCCATATTAGTCATTGGTTATATAGCATAAATCAATATTGGCTATTGGCCATTGCATACGTTGTATCTATATCATAA

TATGTACATTTATATTGGCTCATGTCCAATATGACCGCCAT

1.2 pCEP4 L1-ORFeus (1-17793bp)

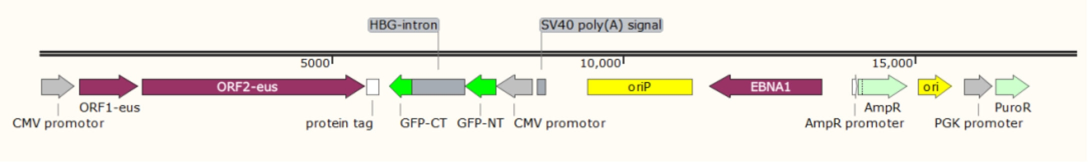

Features	Regions (bp)
CMV promoter	30-605
ORF1 (L1-ORFeus)	676-1692
ORF2 (L1-ORFeus)	1756-5580
**GFP-CT**	**5998-6382**
HBG-intron	6383-7282
**GFP-NT**	**7283-7826**
CMV promoter	7827-8446
SV40 poly(A) signal	8526-8659
EBV-oriP	9388-11177
EBNA1	11479-13404
AmpR	14030-14890
pUC origin	15061-15649
PuroR	16383-16982

GTTGACATTGATTATTGACTAGTTATTAATAGTAATCAATTACGGGGTCATTAGTTCATAGCCCATATATGGAGTTCCGCGTTACGTAACTTACGGT
AAATGGCCCGCCTGGCTGACCGCCCAACGACCCCCGCCCATTGACGTCAATAATGACGTATGTTCCCATAGTAACGCCAATAGGGACTTTCC
ATTGACGTCAATGGGTGGAGTATTTACGGTAAACTGCCCACTTGGCAGTACATCAAGTGTATCATATGCCAAGTCCGCCCCCTATTGACGTCAA
TGACGGTAAATGGCCCGCCTGGCATTATGCCCAGTACATGACCTTACGGGACTTTCCTACTTGGCAGTACATCTACGTATTAGTCATCGCTATT
ACCATGGTGATGCGGTTTTGACAGTACACCAATGGGCGTGGATAGCGGTTTGACTCACGGGGATTTCCAAGTCTCCACCCCATTGACGTCAA
TGGGAGTTTGTTTTGGCACCAAAATCAACGGGACTTTCCAAAATGTCGTAATAACCCCGCCCCGTTGACGCAAATGGGCGGTAGGCGTGTAC
GGTGGGAGGTCTATATAAGCAGAGCTCGTTTAGTGAACCGTCAGATCTCTAGAAGCTGGGTACCAGCTGCTAGCAAGCTTGCTAGCGGCCGC
GTTTAAACTTAATTAAGCCACCATGGGCAAGAAGCAGAACCGCAAGACCGGCAACAGCAAGACCCAGAGCGCCAGCCCCCCCCCAAGGA
GCGCAGCAGCAGCCCCGCCACCGAGCAGAGCTGGATGGAGAACGACTTCGACGAGCTGCGCGAGGAGGGCTTCCGCCGCAGCAACTACA
GCGAGCTGCGCGAGGACATCCAGACCAAGGGCAAGGAGGTGGAGAACTTCGAGAAGAACCTGGAGGAGTGCATCACCCGCATCACCAACA
CCGAGAAGTGCCTGAAGGAGCTGATGGAGCTGAAGACCAAGGCCCGCGAGCTGCGCGAGGAGTGCCGCAGCCTGCGCAGCCGCTGCGA
CCAATTGGAGGAGCGCGTGAGCGCCATGGAGGACGAGATGAACGAGATGAAGCGCGAGGGCAAGTTCCGCGAGAAGCGCATCAAGCGCA
ACGAGCAGAGCCTGCAGGAGATCTGGGACTACGTGAAGCGCCCCAACCTGCGCCTGATCGGCGTGCCCGAGAGCGACGTGGAGAACGGC
ACCAAGCTGGAGAACACCCTGCAGGACATCATCCAGGAGAACTTCCCCAACCTGGCCCGCCAGGCCAACGTGCAGATCCAGGAGATCCAG
CGCACCCCCCAGCGCTACAGCAGCCGCCGCGCCACCCCCCGCCACATCATCGTGCGCTTCACCAAGGTGGAGATGAAGGAGAAGATGCTG
CGCGCCGCCCGCGAGAAGGGCCGCGTGACCCTGAAGGGCAAGCCCATCCGTCTCACCGCCGACCTGAGCGCCGAGACCCTGCAGGCCC
GCCGCGAGTGGGGCCCCATCTTCAACATCCTGAAGGAGAAGAACTTCCAGCCCCGCATCAGCTACCCCGCCAAGCTGAGCTTCATCAGCGA
GGGCGAGATCAAGTACTTCATCGACAAGCAGATGCTGCGCGACTTCGTGACCACCCGCCCCGCCCTGAAGGAGCTGCTGAAGGAGGCCCT
GAACATGGAGCGCAACAACCGCTACCAGCCCCTGCAGAACCACGCCAAGATGTAAAGACCATCGAGACTAGGAAGAAACTGCATCAACTAAT
GAGCAAAATCAGGCGCGCCATCATAATGACCGGCAGCAACAGCCACATCACCATCCTGACCCTGAACATCAACGGCCTGAACAGCGCCAT
CAAGCGCCACCGCCTGGCCAGCTGGATCAAGAGCCAGGACCCCAGCGTGTGCTGCATCCAGGAGACCCACCTGACCTGCCGCGACACCC
ACCGCCTGAAGATCAAGGGCTGGCGCAAGATCTACCAGGCCAACGGCAAGCAGAAGAAGGCCGGCGTGGCCATCCTGGTGAGCGACAAG
ACCGACTTCAAGCCCACCAAGATCAAGCGCGACAAGGAGGGCCACTACATCATGGTGAAGGGCAGCATCCAGCAGGAGGAGCTGACCATC
CTGAACATCTACGCCCCCAACACCGGTGCCCCCCGCTTCATCAAGCAGGTGCTGAGCGACCTGCAGCGCGACCTGGACAGCCACACCCTG
ATCATGGGCGACTTCAACACCCCCCTGAGCACCCTGGACCGCAGCACCCGCCAGAAGGTGAACAAGGACACCCAGGAGCTGAACAGCGCC
CTGCACCAGGCCGACCTGATCGACATCTACCGCACCCTGCACCCCAAGAGCACCGAGTACACCTTCTTCAGCGCCCCCCACCACACCTACA
GCAAGATCGACCACATCGTGGGCAGCAAGGCCCTGCTGAGCAAGTGCAAGCGCACCGAGATCATCACCAACTACCTGAGCGACCACAGCG
CCATCAAGCTGGAGCTGCGCATCAAGAACCTGACCCAGAGCCGCAGCACCACCTGGAAGCTGAACAACCTGCTGCTGAACGACTACTGGG
TGCACAACGAGATGAAGGCCGAGATCAAGATGTTCTTCGAAACCAACGAGAACAAGGACACCACCTACCAGAACCTGTGGGACGCCTTCAA
GGCCGTGTGCCGCGGCAAGTTCATCGCCCTGAACGCCTACAAGCGCAAGCAGGAGCGCAGCAAGATCGACACCCTGACCAGCCAGCTGAA
GGAGCTGGAGAAGCAGGAGCAGACCCACAGCAAGGCCAGCCGCCGCCAGGAGATCACCAAGATCCGCGCCGAGCTGAAGGAGATCGAGA
CCCAGAAGACCCTGCAGAAGATCAACGAGTCGCGAAGCTGGTTCTTCGAGCGCATCAACAAGATCGACCGCCCCCTGGCCCGCCTGATCAA
GAAGAAGCGCGAGAAGAACCAGATCGACACCATCAAGAACGACAAGGGCGACATCACCACCGACCCCACCGAGATCCAGACCACCATCCG
CGAGTACTACAAGCACCTGTACGCCAACAAGCTGGAGAACCTGGAGGAGATGGACACCTTCCTGGACACCTACACCCTGCCCCGCCTGAAC
CAGGAGGAGGTGGAGAGCCTGAACCGCCCCATCACCGGCAGCGAGATCGTGGCCATCATCAACAGCCTGCCCACCAAGAAGAGCCCCGG
CCCCGACGGCTTCACCGCCGAGTTCTACCAGCGCTACAAGGAGGAGCTGGTGCCCTTCCTGCTGAAGCTGTTCCAGAGCATCGAGAAGGA
GGGCATCCTGCCCAACAGCTTCTACGAGGCCAGCATCATCCTGATCCCCAAGCCCGGCCGCGACACCACCAAGAAGGAGAACTTCCGCCC
CATCAGCCTGATGAACATCGACGCCAAGATCCTGAACAAGATCCTGGCCAACCGCATCCAGCAGCACATCAAGAAGCTGATCCACCACGACC
AGGTGGGCTTCATCCCCGGGATGCAGGGCTGGTTCAACATCCGCAAGAGCATCAACGTGATCCAGCACATCAACCGCGCCAAGGACAAGAA
CCACATGATCATCAGCATCGACGCCGAGAAGGCCTTCGACAAGATCCAGCAGCCCTTCATGCTGAAGACCCTGAACAAGCTGGGCATCGAC
GGCACCTACTTCAAGATCATCCGCGCCATCTACGACAAGCCCACCGCCAACATCATCCTGAACGGCCAGAAGCTGGAGGCCTTCCCCCTGA
AGACCGGCACGCGTCAGGGCTGCCCCCTGAGCCCCCTGCTGTTCAACATCGTGCTGGAGGTGCTGGCCCGCGCCATCCGCCAGGAGAAG

GAGATCAAGGGCATCCAGCTGGGCAAGGAGGAGGTGAAGCTGAGCCTGTTCGCCGACGACATGATCGTGTACCTGGAGAACCCCATCGTG
AGCGCCCAGAACCTGCTGAAGCTGATCAGCAACTTCAGCAAGGTGAGCGGCTACAAGATCAACGTGCAGAAGAGCCAGGCCTTCCTGTACA
CCAACAACCGCCAGACCGAGAGCCAGATCATGGGCGAGCTGCCCTTCACCATCGCTAGCAAGCGCATCAAGTACCTGGGCATCCAGCTGAC
CCGCGACGTGAAGGACCTGTTCAAGGAGAACTACAAGCCCCTGCTGAAGGAGATCAAGGAGGAGACCAACAAGTGGAAGAACATCCCCTG
CAGCTGGGTGGGCCGCATCAACATCGTGAAGATGGCCATCCTGCCCAAGGTGATCTACCGCTTCAACGCCATCCCCATCAAGCTGCCCATG
ACCTTCTTCACCGAGCTGGAGAAGACCACCCTGAAGTTCATCTGGAACCAGAAGCGCGCCCGCATCGCCAAGAGCATCCTGAGCCAGAAGA
ACAAGGCCGGCGGCATCACCCTGCCCGACTTCAAGCTGTACTACAAGGCCACCGTGACCAAGACCGCCTGGTACTGGTACCAGAACCGCG
ATATCGACCAGTGGAACCGCACCGAGCCCAGCGAGATCATGCCCCACATCTACAACTACCTGATCTTCGACAAGCCCGAGAAGAACAAGCAG
TGGGGCAAGGACAGCCTGTTCAACAAGTGGTGCTGGGAGAACTGGCTGGCCATCTGCCGCAAGCTGAAGCTGGACCCCTTCCTGACCCCC
TACACCAAGATCAACAGCCGCTGGATCAAGGACCTGAACGTGAAGCCCAAGACCATCAAGACCCTGGAGGAGAACCTGGGCATCACCATCC
AGGACATCGGCGTGGGCAAGGACTTCATGAGCAAGACCCCCAAGGCCATGGCCACCAAGGACAAGATCGACAAGTGGGACCTGATCAAGC
TGAAGAGCTTCTGCACCGCCAAGGAGACCACCATCCGCGTGAACCGCCAGCCCACCACCTGGGAGAAGATCTTCGCCACCTACAGCAGCG
ACAAGGGCCTGATCAGCCGCATCTACAACGAGCTGAAGCAGATCTACAAGAAGAAGACCAACAACCCCATCAAGAAGTGGGCCAAGGACAT
GAACCGCCACTTCAGCAAGGAGGACATCTACGCCGCCAAGAAGCATATGAAGAAGTGCAGCAGCAGCCTGGCCATCCGCGAGATGCAGATC
AAGACCACCATGCGCTACCACCTGACCCCCGTGCGCATGGCCATCATCAAGAAGAGCGGCAACAACCGCTGCTGGCGCGGCTGCGGCGAG
ATCGGCACCCTGCTGCACTGCTGGTGGGACTGCAAGCTGGTGCAGCCCCTGTGGAAGAGCGTGTGGCGCTTCCTGCGCGACCTGGAGCT
GGAGATCCCCTTCGACCCCGCCATCCCCCTGCTGGGCATCTACCCCAACGAGTACAAGAGCTGCTGCTACAAGGACACCTGCACCCGCATG
TTCATCGCCGCCCTGTTCACCATCGCCAAGACCTGGAACCAGCCCAAGTGCCCCACCATGATCGATTGGATCAAGAAGATGTGGCACATCTA
CACCATGGAGTACTACGCCGCCATCAAGAACGACGAGTTCATCAGCTTCGTGGGCACCTGGATGAAGCTGGAGACCATCATCCTGAGCAAG
CTGAGCCAGGAGCAGAAGACCAAGCACCGCATCTTCAGCCTGATCGGCGGCAACGGCGGCGGCAGCGGCGGCGGCAGCCATCACCATCA
CCATCACCATCACCTGGAAGTTCTGTTCCAGGGGCCCCTGGAAGTTCTGTTCCAGGGGCCCCTTAAGGAACAGAAGCTAATCTCAGAAGAA
GACCTGGAACAGAAGCTAATCTCAGAAGAAGACCTGGACTACAAAGACCATGACGGTGATTATAAAGATCATGACATCGATTACAAGGATGAC
GATGACAAGTAGATTTAAATACAATGAGATCACATGGACACAGGAAGGGGAATATCACACTCTGGGGACTGTGGTGGGGTCGGGGGAGGGG
GGAGGGATAGCATTGGGAGATATACCTAATGCTAGATGACACATTAGTGGGTGCAGCGCACCAGCATGGCACATGTAAAACGACGGATCGATC
CGAACAAACGACCC**AACACCCGTGCGTTTTATTCTGTCTTTTTATTGCCGATCCCCTCAGAAGAACTCGTCAAGAAGGCGATAGAAGGCGA**
TGCGCTGCGAATCGGGAGCGGCGATACCGTAAAGCACGAGGAAGCGGTCAGCCCATTCGCCGCCAAGCTCTTCAGCAATATCACGGGTA
GCCAACGCTATGTCCTGATAGCGGTCGGCCGCTTTACTTGTACAGCTCGTCCATGCCGAGAGTGATCCCGGCGGCGGTCACGAACTCCAG
CAGGACCATGTGATCGCGCTTCTCGTTGGGGTCTTTGCTCAGGGCGGACTGGGTGCTCAGGTAGTGGTTGTCGGGCAGCAGCACGGGGC
CGTCGCCGATGGGGGTGTTCTGCTGGTAGTGGTCGGCCAGGTGAGTCCAGGAGATGTTTCAGCACTGTTGCCTTTAGTCTCGAGGCAACTT
AGACAACTGAGTATTGATCTGAGCACAGCAGGGTGTGAGCTGTTTGAAGATACTGGGGTTGGGGGTGAAGAAACTGCAGAGGACTAACTGG
GCTGAGACCCAGTGGCAATGTTTTAGGGCCTAAGGAATGCCTCTGAAAATCTAGATGGACAACTTTGACTTTGAGAAAAGAGAGGTGGAAAT
GAGGAAAATGACTTTTCTTTATTAGATTTCGGTAGAAAGAACTTTCATCTTTCCCCTATTTTTGTTATTCGTTTTAAAACATCTATCTGGAGGCAG
GACAAGTATGGTCATTAAAAAGATGCAGGCAGAAGGCATATATTGGCTCAGTCAAAGTGGGGAACTTTGGTGGCCAAACATACATTGCTAAGG
CTATTCCTATATCAGCTGGACACATATAAAATGCTGCTAATGCTTCATTACAAACTTATATCCTTTAATTCCAGATGGGGGCAAAGTATGTCCAGG
GGTGAGGAACAATTGAAACATTTGGGCTGGAGTAGATTTTGAAAGTCAGCTCTGTGTGTGTGTGTGTGTGTGTGTGTGTGTGTGTGCGCGCA
CGTGTGTTTGTGTGTGTGTGAGAGCGTGTGTTTCTTTTAACGTTTTCAGCCTACAGCATACAGGGTTCATGGTGGCAAGAAGATAACAAGATT
TAAATTATGGCCAGTGACTAGTGCTGCAAGAAGAACAACTACCTGCATTTAATGGGAAAGCAAAATCTCAGGCTTTGAGGGAAGTTAACATAGG
CTTGATTCTGGGTGGAAGCTGGGTGTGTAGTTATCTGGAGGCCAGGCTGGAGCTCTCAGCTCACTATGGGTTCATCTTTATTGTCTCCTTTCA
TCTCAACAG**CTGCACGCTGCCGTCCTCGATGTTGTGGCGGATCTTGAAGTTCACCTTGATGCCGTTCTTCTGCTTGTCGGCCATGATATAGA**
CGTTGTGGCTGTTGTAGTTGTACTCCAGCTTGTGCCCCAGGATGTTGCCGTCCTCCTTGAAGTCGATGCCCTTCAGCTCGATGCGGTTCAC
CAGGGTGTCGCCCTCGAACTTCACCTCGGCGCGGGTCTTGTAGTTGCCGTCGTCCTTGAAGAAGATGGTGCGCTCCTGGACGTAGCCTTC
GGGCATGGCGGACTTGAAGAAGTCGTGCTGCTTCATGTGGTCGGGGTAGCGGCTGAAGCACTGCACGCCGTAGGTCAGGGTGGTCACG
AGGGTGGGCCAGGGCACGGGCAGCTTGCCGGTGGTGCAGATGAACTTCAGGGTCAGCTTGCCGTAGGTGGCATCGCCCTCGCCCTCGC
CGGACACGCTGAACTTGTGGCCGTTTACGTCGCCGTCCAGCTCGACCAGGATGGGCACCACCCCGGTGAACAGCTCCTCGCCCTTGCTC
ACCATGGTGGCGAATTCGAAGCTTGAGCTCGAGATCTGAGTCCGGTAGCGCTAGCGGATCTGACGGTTCACTAAACCAGCTCTGCTTATATA
GACCTCCCACCGTACACGCCTACCGCCCATTTGCGTCAATGGGGCGGAGTTGTTACGACATTTTGGAAAGTCCCGTTGATTTTGGTGCCAAA
ACAAACTCCCATTGACGTCAATGGGGTGGAGACTTGGAAATCCCCGTGAGTCAAACCGCTATCCACGCCCATTGATGTACTGCCAAAACCGC
ATCACCATGGTAATAGCGATGACTAATACGTAGATGTACTGCCAAGTAGGAAAGTCCCATAAGGTCATGTACTGGGCATAATGCCAGGCGGGC
CATTTACCGTCATTGACGTCAATAGGGGGCGTACTTGGCATATGATACACTTGATGTACTGCCAAGTGGGCAGTTTACCGTAAATACTCCACCC
ATTGACGTCAATGGAAAGTCCCTATTGGCGTTACTATGGGAACATACGTCATTATTGACGTCAATGGGCGGGGGTCGTTGGGCGGTCAGCCA
GGCGGGCCATTTACCGTAAGTTATGTAACGCGGAACTCCATATATGGGCTATGAACTAATGACCCCGTAATTGATTACTATTAGCCCGGGCAAT
GTGCACATGTACCCTAAAACTTAAAGTATAATAAAGACGTCAGGGTTCGAAATCGATAAGCTTGGATCCAGACATGATAAGATACATTGATGAGT
TTGGACAAACCACAACTAGAATGCAGTGAAAAAAATGCTTTATTTGTGAAATTTGTGATGCTATTGCTTTATTTGTAACCATTATAAGCTGCAATA
AACAAGTTAACAACAACAATTGCATTCATTTTATGTTTCAGGTTCAGGGGGAGGTGTGGGAGGTTTTTTAAAGCAAGTAAAACCTCTACAAATG
TGGTATGGCTGATTATGATCCGGCTGCCTCGCGCGTTTCGGTGATGACGGTGAAAACCTCTGACACATGCAGCTCCCGGAGACGGTCACAG
CTTGTCTGTAAGCGGATGCCGGGAGCAGACAAGCCCGTCAGGGCGCGTCAGCGGGTGTTGGCGGGTGTCGGGGCGCAGCCATGAGGTCG
ATCGACTCTAGAGGATCGATCCCCGCCCCGGACGAACTAAACCTGACTACGACATCTCTGCCCCTTCTTCGCGGGGCAGTGCATGTAATCCC
TTCAGTTGGTTGGTACAACTTGCCAACTGGGCCCTGTTCCACATGTGACACGGGGGGGGACCAAACACAAAGGGGTTCTCTGACTGTAGTT
GACATCCTTATAAATGGATGTGCACATTTGCCAACACTGAGTGGCTTTCATCCTGGAGCAGACTTTGCAGTCTGTGGACTGCAACACAACATT
GCCTTTATGTGTAACTCTTGGCTGAAGCTCTTACACCAATGCTGGGGGACATGTACCTCCCAGGGGCCCAGGAAGACTACGGGAGGCTACAC
CAACGTCAATCAGAGGGGCCTGTGTAGCTACCGATAAGCGGACCCTCAAGAGGGCATTAGCAATAGTGTTTATAAGGCCCCCTTGTTAACCCT
AAACGGGTAGCATATGCTTCCCGGGTAGTAGTATATACTATCCAGACTAACCCTAATTCAATAGCATATGTTACCCAACGGGAAGCATATGCTATC
GAATTAGGGTTAGTAAAAGGGTCCTAAGGAACAGCGATATCTCCCACCCCATGAGCTGTCACGGTTTTATTTACATGGGGTCAGGATTCCACG
AGGGTAGTGAACCATTTTAGTCACAAGGGCAGTGGCTGAAGATCAAGGAGCGGGCAGTGAACTCTCCTGAATCTTCGCCTGCTTCTTCATTC
TCCTTCGTTTAGCTAATAGAATAACTGCTGAGTTGTGAACAGTAAGGTGTATGTGAGGTGCTCGAAAACAAGGTTTCAGGTGACGCCCCCAGA
ATAAAATTTGGACGGGGGGTTCAGTGGTGGCATTGTGCTATGACACCAATATAACCCTCACAAACCCCTTGGGCAATAAATACTAGTGTAGGAA
TGAAACATTCTGAATATCTTTAACAATAGAAATCCATGGGGTGGGGACAAGCCGTAAAGACTGGATGTCCATCTCACACGAATTTATGGCTATG
GGCAACACATAATCCTAGTGCAATATGATACTGGGGTTATTAAGATGTGTCCCAGGCAGGGACCAAGACAGGTGAACCATGTTGTTACACTCTA
TTTGTAACAAGGGGAAAGAGAGTGGACGCCGACAGCAGCGGACTCCACTGGTTGTCTCTAACACCCCCGAAAATTAAACGGGGCTCCACGC

CAATGGGGCCCATAAACAAAGACAAGTGGCCACTCTTTTTTTTGAAATTGTGGAGTGGGGGCACGCGTCAGCCCCCACACGCCGCCCTGCG
GTTTTGGACTGTAAAATAAGGGTGTAATAACTTGGCTGATTGTAACCCCGCTAACCACTGCGGTCAAACCACTTGCCCACAAAACCACTAATG
GCACCCCGGGGAATACCTGCATAAGTAGGTGGGCGGGCCAAGATAGGGGCGCGATTGCTGCGATCTGGAGGACAAATTACACACACTTGCG
CCTGAGCGCCAAGCACAGGGTTGTTGGTCCTCATATTCACGAGGTCGCTGAGAGCACGGTGGGCTAATGTTGCCATGGGTAGCATATACTAC
CCAAATATCTGGATAGCATATGCTATCCTAATCTATATCTGGGTAGCATAGGCTATCCTAATCTATATCTGGGTAGCATATGCTATCCTAATCTATAT
CTGGGTAGTATATGCTATCCTAATTTATATCTGGGTAGCATAGGCTATCCTAATCTATATCTGGGTAGCATATGCTATCCTAATCTATATCTGGGTAG
TATATGCTATCCTAATCTGTATCCGGGTAGCATATGCTATCCTAATAGAGATTAGGGTAGTATATGCTATCCTAATTTATATCTGGGTAGCATATACT
ACCCAAATATCTGGATAGCATATGCTATCCTAATCTATATCTGGGTAGCATATGCTATCCTAATCTATATCTGGGTAGCATAGGCTATCCTAATCTAT
ATCTGGGTAGCATATGCTATCCTAATCTATATCTGGGTAGTATATGCTATCCTAATTTATATCTGGGTAGCATAGGCTATCCTAATCTATATCTGGGT
AGCATATGCTATCCTAATCTATATCTGGGTAGTATATGCTATCCTAATCTGTATCCGGGTAGCATATGCTATCCTCATGCATATACAGTCAGCATATG
ATACCCAGTAGTAGAGTGGGAGTGCTATCCTTTGCATATGCCGCCACCTCCCAAGGGGGCGTGAATTTTCGCTGCTTGTCCTTTTCCTGCTGG
TTGCTCCCATTCTTAGGTGAATTTAAGGAGGCCAGGCTAAAGCCGTCGCATGTCTGATTGCTCACCAGGTAAATGTCGCTAATGTTTTCCAAC
GCGAGAAGGTGTTGAGCGCGGAGCTGAGTGACGTGACAACATGGGTATGCCCAATTGCCCCATGTTGGGAGGACGAAAATGGTGACAAGA
CAGATGGCCAGAAATACACCAACAGCACGCATGATGTCTACTGGGGATTTATTCTTTAGTGCGGGGGAATACACGGCTTTTAATACGATTGAG
GGCGTCTCCTAACAAGTTACATCACTCCTGCCCTTCCTCACCCTCATCTCCATCACCTCCTTCATCTCCGTCATCTCCGTCATCACCCTCCGC
GGCAGCCCCTTCCACCATAGGTGGAAACCAGGGAGGCAAATCTACTCCATCGTCAAAGCTGCACACAGTCACCCTGATATTGCAGGTAGGAG
CGGGCTTTGTCATAACAAGGTCCTTAATCGCATCCTTCAAAACCTCAGCAAATATATGAGTTTGTAAAAAGACCATGAAATAACAGACAATGGAC
TCCCTTAGCGGGCCAGGTTGTGGGCCGGGTCCAGGGGCCATTCCAAAGGGGAGACGACTCAATGGTGTAAGACGACATTGTGGAATAGCAA
GGGCAGTTCCTCGCCTTAGGTTGTAAAGGGAGGTCTTACTACCTCCATATACGAACACACCGGCGACCCAAGTTCCTTCGTCGGTAGTCCTTT
CTACGTGACTCCTAGCCAGGAGAGCTCTTAAACCTTCTGCAATGTTCTCAAATTTCGGGTTGGAACCTCCTTGACCACGATGCTTTCCAAACC
ACCCTCCTTTTTTGCGCCTGCCTCCATCACCCTGACCCCGGGGTCCAGTGCTTGGGCCTTCTCCTGGGTCATCTGCGGGGCCCTGCTCTAT
CGCTCCCGGGGGCACGTCAGGCTCACCATCTGGGCCACCTTCTTGGTGGTATTCAAAATAATCGGCTTCCCCTACAGGGTGGAAAAATGGC
CTTCTACCTGGAGGGGGCCTGCGCGGTGGAGACCCGGATGATGATGACTGACTACTGGGACTCCTGGGCCTCTTTTCTCCACGTCCACGAC
CTCTCCCCCTGGCTCTTTCACGACTTCCCCCCCTGGCTCTTTCACGTCCTCTACCCCGGCGGCCTCCACTACCTCCTCGACCCCGGCCTCC
ACTACCTCCTCGACCCCGGCCTCCACTGCCTCCTCGACCCCGGCCTCCACCTCCTGCTCCTGCCCCTCCTGCTCCTGCCCCTCCTCCTGCT
CCTGCCCCTCCTGCCCCTCCTGCTCCTGCCCCTCCTGCCCCTCCTGCTCCTGCCCCTCCTGCCCCTCCTGCTCCTGCCCCTCCTGCCCCTC
CTCCTGCTCCTGCCCCTCCTGCCCCTCCTCCTGCTCCTGCCCCTCCTGCCCCTCCTGCTCCTGCCCCTCCTGCCCCTCCTGCTCCTGCCCC
TCCTGCCCCTCCTGCTCCTGCCCCTCCTGCTCCTGCCCCTCCTGCTCCTGCCCCTCCTGCTCCTGCCCCTCCTGCCCCTCCTGCCCCTCCT
CCTGCTCCTGCCCCTCCTGCTCCTGCCCCTCCTGCCCCTCCTGCCCCTCCTGCTCCTGCCCCTCCTCCTGCTCCTGCCCCTCCTGCCCCTC
CTGCCCCTCCTCCTGCTCCTGCCCCTCCTGCCCCTCCTCCTGCTCCTGCCCCTCCTCCTGCTCCTGCCCCTCCTGCCCCTCCTGCCCCTCC
TCCTGCTCCTGCCCCTCCTGCCCCTCCTCCTGCTCCTGCCCCTCCTCCTGCTCCTGCCCCTCCTGCCCCTCCTGCCCCTCCTCCTGCTCCT
GCCCCTCCTCCTGCTCCTGCCCCTCCTGCCCCTCCTGCCCCTCCTGCCCCTCCTCCTGCTCCTGCCCCTCCTCCTGCTCCTGCCCCTCCTG
CTCCTGCCCCTCCCGCTCCTGCTCCTGCTCCTGTTCCACCGTGGGTCCCTTTGCAGCCAATGCAACTTGGACGTTTTTGGGGTCTCCGGAC
ACCATCTCTATGTCTTGGCCCTGATCCTGAGCCGCCCGGGGCTCCTGGTCTTCCGCCTCCTCGTCCTCGTCCTCTTCCCCGTCCTCGTCCAT
GGTTATCACCCCCTCTTCTTTGAGGTCCACTGCCGCCGGAGCCTTCTGGTCCAGATGTGTCTCCCTTCTCTCCTAGGCCATTTCCAGGTCCT
GTACCTGGCCCCTCGTCAGACATGATTCACACTAAAAGAGATCAATAGACATCTTTATTAGACGACGCTCAGTGAATACAGGGAGTGCAGACT
CCTGCCCCCTCCAACAGCCCCCCCACCCTCATCCCCTTCATGGTCGCTGTCAGACAGATCCAGGTCTGAAAATTCCCCATCCTCCGAACCAT
CCTCGTCCTCATCACCAATTACTCGCAGCCCGGAAAACTCCCGCTGAACATCCTCAAGATTTGCGTCCTGAGCCTCAAGCCAGGCCTCAAAT
TCCTCGTCCCCCTTTTTGCTGGACGGTAGGGATGGGGATTCTCGGGACCCCTCCTCTTCCTCTTCAAGGTCACCAGACAGAGATGCTACTGG
GGCAACGGAAGAAAAGCTGGGTGCGGCCTGTGAGGATCAGCTTATCGATGATAAGCTGTCAAACATGAGAATTCTTGAAGACGAAAGGGCCT
CGTGATACGCCTATTTTTATAGGTTAATGTCATGATAATAATGGTTTCTTAGACGTCAGGTGGCACTTTTCGGGGAAATGTGCGCGGAACCCCTA
TTTGTTTATTTTTCTAAATACATTCAAATATGTATCCGCTCATGAGACAATAACCCTGATAAATGCTTCAATAATATTGAAAAAGGAAGAGTATGAG
TATTCAACATTTCCGTGTCGCCCTTATTCCCTTTTTTGCGGCATTTTGCCTTCCTGTTTTTGCTCACCCAGAAACGCTGGTGAAAGTAAAAGAT
GCTGAAGATCAGTTGGGTGCACGAGTGGGTTACATCGAACTGGATCTCAACAGCGGTAAGATCCTTGAGAGTTTTCGCCCCGAAGAACGTTT
TCCAATGATGAGCACTTTTAAAGTTCTGCTATGTGGCGCGGTATTATCCCGTGTTGACGCCGGGCAAGAGCAACTCGGTCGCCGCATACACTA
TTCTCAGAATGACTTGGTTGAGTACTCACCAGTCACAGAAAAGCATCTTACGGATGGCATGACAGTAAGAGAATTATGCAGTGCTGCCATAAC
CATGAGTGATAACACTGCGGCCAACTTACTTCTGACAACGATCGGAGGACCGAAGGAGCTAACCGCTTTTTTGCACAACATGGGGGATCATG
TAACTCGCCTTGATCGTTGGGAACCGGAGCTGAATGAAGCCATACCAAACGACGAGCGTGACACCACGATGCCTGCAGCAATGGCAACAAC
GTTGCGCAAACTATTAACTGGCGAACTACTTACTCTAGCTTCCCGGCAACAATTAATAGACTGGATGGAGGCGGATAAAGTTGCAGGACCACT
TCTGCGCTCGGCCCTTCCGGCTGGCTGGTTTATTGCTGATAAATCTGGAGCCGGTGAGCGTGGGTCTCGCGGTATCATTGCAGCACTGGGG
CCAGATGGTAAGCCCTCCCGTATCGTAGTTATCTACACGACGGGGAGTCAGGCAACTATGGATGAACGAAATAGACAGATCGCTGAGATAGGT
GCCTCACTGATTAAGCATTGGTAACTGTCAGACCAAGTTTACTCATATATACTTTAGATTGATTTAAAACTTCATTTTTAATTTAAAAGGATCTAGG
TGAAGATCCTTTTTGATAATCTCATGACCAAAATCCCTTAACGTGAGTTTTCGTTCCACTGAGCGTCAGACCCCGTAGAAAAGATCAAAGGATC
TTCTTGAGATCCTTTTTTTCTGCGCGTAATCTGCTGCTTGCAAACAAAAAAACCACCGCTACCAGCGGTGGTTTGTTTGCCGGATCAAGAGCT
ACCAACTCTTTTTCCGAAGGTAACTGGCTTCAGCAGAGCGCAGATACCAAATACTGTCCTTCTAGTGTAGCCGTAGTTAGGCCACCACTTCAA
GAACTCTGTAGCACCGCCTACATACCTCGCTCTGCTAATCCTGTTACCAGTGGCTGCTGCCAGTGGCGATAAGTCGTGTCTTACCGGGTTGG
ACTCAAGACGATAGTTACCGGATAAGGCGCAGCGGTCGGGCTGAACGGGGGGTTCGTGCACACAGCCCAGCTTGGAGCGAACGACCTACA
CCGAACTGAGATACCTACAGCGTGAGCTATGAGAAAGCGCCACGCTTCCCGAAGGGAGAAAGGCGGACAGGTATCCGGTAAGCGGCAGGG
TCGGAACAGGAGAGCGCACGAGGGAGCTTCCAGGGGGAAACGCCTGGTATCTTTATAGTCCTGTCGGGTTTCGCCACCTCTGACTTGAGCG
TCGATTTTTGTGATGCTCGTCAGGGGGGCGGAGCCTATGGAAAAACGCCAGCAACGCGGCCTTTTTACGGTTCCTGGCCTTTTGCTGGCCTT
GAAGCTGTCCCTGATGGTCGTCATCTACCTGCCTGGACAGCATGGCCTGCAACGCGGGCATCCCGATGCCGCCGGAAGCGAGAAGAATCAT
AATGGGGAAGGCCATCCAGCCTCGCGTCGAATTCGTCGACCTCGAAATTCTACCGGGTAGGGGAGGCGCTTTTCCCAAGGCAGTCTGGAGC
ATGCGCTTTAGCAGCCCCGCTGGGCACTTGGCGCTACACAAGTGGCCTCTGGCCTCGCACACATTCCACATCCACCGGTAGGCGCCAACCG
GCTCCGTTCTTTGGTGGCCCCTTCGCGCCACCTTCTACTCCTCCCCTAGTCAGGAAGTTCCCCCCCGCCCCGCAGCTCGCGTCGTGCAGG
ACGTGACAAATGGAAGTAGCACGTCTCACTAGTCTCGTGCAGATGGACAGCACCGCTGAGCAATGGAAGCGGGTAGGCCTTTGGGGCAGC
GGCCAATAGCAGCTTTGCTCCTTCGCTTTCTGGGCTCAGAGGCTGGGAAGGGGTGGGTCCGGGGGCGGGCTCAGGGGCGGGCTCAGGG
GCGGGGCGGGCGCCCGAAGGTCCTCCGGAGGCCCGGCATTCTGCACGCTTCAAAAGCGCACGTCTGCCGCGCTGTTCTCCTCTTCCTCAT
CTCCGGGCCTTTCGACCTGCATCCATCTAGATCTCGAGCAGCTGAAGCTTACCATGACCGAGTACAAGCCCACGGTGCGCCTCGCCACCCG
CGACGACGTCCCCAGGGCCGTACGCACCCTCGCCGCCGCGTTCGCCGACTACCCCGCCACGCGCCACACCGTCGATCCGGACCGCCACA

TCGAGCGGGTCACCGAGCTGCAAGAACTCTTCCTCACGCGCGTCGGGCTCGACATCGGCAAGGTGTGGGTCGCGGACGACGGCGCCGCG
GTGGCGGTCTGGACCACGCCGGAGAGCGTCGAAGCGGGGGCGGTGTTCGCCGAGATCGGCCCGCGCATGGCCGAGTTGAGCGGTTCCC
GGCTGGCCGCGCAGCAACAGATGGAAGGCCTCCTGGCGCCGCACCGGCCCAAGGAGCCCGCGTGGTTCCTGGCCACCGTCGGCGTCTC
GCCCGACCACCAGGGCAAGGGTCTGGGCAGCGCCGTCGTGCTCCCCGGAGTGGAGGCGGCCGAGCGCGCCGGGGTGCCCGCCTTCCT
GGAGACCTCCGCGCCCCGCAACCTCCCCTTCTACGAGCGGCTCGGCTTCACCGTCACCGCCGACGTCGAGGTGCCCGAAGGACCGCGCA
CCTGGTGCATGACCCGCAAGCCCGGTGCCTGACGCCCGCCCCACGACCCGCAGCGCCCGACCGAAAGGAGCGCACGACCCCATGCATCG
ATGATATCAGATCCCCGGGATGCAGAAATTGATGATCTATTAAACAATAAAGATGTCCACTAAAATGGAAGTTTTTCCTGTCATACTTTGTTAAGA
AGGGTGAGAACAGAGTACCTACATTTTGAATGGAAGGATTGGAGCTACGGGGGTGGGGGTGGGGTGGGATTAGATAAATGCCTGCTCTTTAC
TGAAGGCTCTTTACTATTGCTTTATGATAATGTTTCATAGTTGGATATCATAATTTAAACAAGCAAAACCAAATTAAGGGCCAGCTCATTCCTCCC
ACTCATGATCTATAGATCTATAGATCTCTCGTGGGATCATTGTTTTTCTCTTGATTCCCACTTTGTGGTTCTAAGTACTGTGGTTTCCAAATGTGT
CAGTTTCATAGCCTGAAGAACGAGATCAGCAGCCTCTGTTCCACATACACTTCATTCTCAGTATTGTTTTGCCAAGTTCTAATTCCATCAGAAG
CTGGTCGACATTGGATTGGAAGTAGTATACTAACCTTCACCTATTGCAATTCTCATGTTTGACAGCTTATCATCGCAGATCCGGGCAACGTTGTT
GCCATTGCTGCAGGCGCAGAACTGGTAGGTATGGAAGATCTATACATTGAATCAATATTGGCAATTAGCCATATTAGTCATTGGTTATATAGCATA
AATCAATATTGGCTATTGGCCATTGCATACGTTGTATCTATATCATAATATGTACATTTATATTGGCTCATGTCCAATATGACCGCCAT

1.3 pCEP4 L1-ORFeus-ENm (H230A) (L1-ENm) and pCEP4 L1-ORFeus-RTm (D702Y) (L1-RTm) plasmids share the same sequences as pCEP4 L1-ORFeus except for the following minor key changes:

	**EN (2443-2445 bp)**	**RT (3859-3861bp)**
**pCEP4 L1-ORFeus**	H230 (CAC)	D702 (GAC)
**pCEP4 L1-ENm (H230A)**	A230 (GCT)	D702 (GAC)
**pCEP4 L1-RTm (D702Y)**	H230 (CAC)	Y702 (TAC)

Supplementary Sequence 2. DNA sequences of endogenous human L1 (i.e., L1Hs and L1RP)

2.1 L1Hs

The human LINE-1 element (L1 Homo sapiens, or L1Hs) is the most active autonomous retrotransposon family in the human genome (it's got an ACG, making it an L1PA2).

Features	Regions (bp)
L1 5'UTR	1-907
ORF1 (L1Hs)	908-1924
ORF2 (L1Hs)	1988-5815
L1 3'UTR	5816-6064

GGGAGGAGGAGCCAAGATGGCCGAATAGGAACAGCTCCGGTCTACAGCTCCCAGCGTGAGCGACGCAGAAGACGGTGATTTCTGCATTT
CCATCTGAGGTACCGGGTTCATCTCACTAGGGAGTGCCAGACAGTGGGCGCAGGCCAGTGTGTGTGCGCACCGTGCGCGAGCCGAAGCA
GGGCGAGGCATTGCCTCACCTGGGAAGCGCAAGGGGTCAGGGAGTTCCCTTTCCGAGTCAAAGAAAGGGGTGACGGACGCACCTGGAAA
ATCGGGTCACTCCCACCCGAATATTGCGCTTTTCAGACCGGCTTAAGAAACGGCGCACCACGAGACTATATCCCACACCTGGCTCAGAGGGT
CCTACGCCCACGGAATCTCGCTGATTGCTAGCACAGCAGTCTGAGATCAAACTGCAAGGCGGCAACGAGGCTGGGGGAGGGGCGCCCGCC
ATTGCCCAGGCTTGCTTAGGTAAACAAAGCAGCCGGGAAGCTCGAACTGGGTGGAGCCCACCACAGCTCAAGGAGGCCTGCCTGCCTCTG
TAGGCTCCACCTCTGGGGGCAGGGCACAGACAAACAAAAAGACAGCAGTAACCTCTGCAGACTTAAGTGTCCCTGTCTGACAGCTTTGAAG
AGAGCAGTGGTTCTCCCAGCACGCAGCTGGAGATCTGAGAACGGGCAGACTGCCTCCTCAAGTGGGTCCCTGACCCCTGACCCCCGAGCA
GCCTAACTGGGAGGCACCCCCCAGCAGGGGCACACTGACACCTCACACGGCAGGGTATTCCAACAGACCTGCAGCTGAGGGTCCTGTCTG
TTAGAAGGAAAACTAACAACCAGAAAGGACATCTACACCGAAAACCCATCTGTACATCACCATCATCAAAGACCAAAAGTAGATAAAACCACAA
AGATGGGGAAAAAACAGAACAGAAAAACTGGAAACTCTAAAACGCAGAGCGCCTCTCCTCCTCCAAAGGAACGCAGTTCCTCACCAGCAAC
AGAACAAAGCTGGATGGAGAATGATTTTGACGAGCTGAGAGAAGAAGGCTTCAGACGATCAAATTACTCTGAGCTACGGGAGGACATTCAAA
CCAAAGGCAAAGAAGTTGAAAACTTTGAAAAAAATTTAGAAGAATGTATAACTAGAATAACCAATACAGAGAAGTGCTTAAAGGAGCTGATGGA
GCTGAAAACCAAGGCTCGAGAACTACGTGAAGAATGCAGAAGCCTCAGGAGCCGATGCGATCAACTGGAAGAAAGGGTATCAGCAATGGAA
GATGAAATGAATGAAATGAAGCGAGAAGGGAAGTTTAGAGAAAAAAGAATAAAAAGAAATGAGCAAAGCCTCCAAGAAATATGGGACTATGTG
AAAAGACCAAATCTACGTCTGATTGGTGTACCTGAAAGTGATGTGGAGAATGGAACCAAGTTGGAAAACACTCTGCAGGATATTATCCAGGAG
AACTTCCCCAATCTAGCAAGGCAGGCCAACGTTCAGATTCAGGAAATACAGAGAACGCCACAAAGATACTCCTCGAGAAGAGCAACTCCAAG
ACACATAATTGTCAGATTCACCAAAGTTGAAATGAAGGAAAAAATGTTAAGGGCAGCCAGAGAGAAAGGTCGGGTTACCCTCAAAGGAAAGC
CCATCAGACTAACAGCGGATCTCTCGGCAGAAACCCTACAAGCCAGAAGAGAGTGGGGGCCAATATTCAACATTCTTAAAGAAAAGAATTTTC
AACCCAGAATTTCATATCCAGCCAAACTAAGCTTCATAAGTGAAGGAGAAATAAAATACTTTATAGACAAGCAAATGCTGAGAGATTTTGTCACC
ACCAGGCCTGCCCTAAAAGAGCTCCTGAAGGAAGCGCTAAACATGGAAAGGAACAACCGGTACCAGCCGCTGCAAAATCATGCCAAAATGTA
AAGACCATCGAGACTAGGAAGAAACTGCATCAACTAATGAGCAAAATCACCAGCTAACATCATAATGACAGGATCAAATTCACACATAACAATAT
TAACTTTAAATATAAATGGACTAAATTCTGCAATTAAAAGACACAGACTGGCAAGTTGGATAAAGAGTCAAGACCCATCAGTGTGCTGTATTCAG
GAAACCCATCTCACGTGCAGAGACACACATAGGCTCAAAATAAAAGGATGGAGGAAGATCTACCAAGCCAATGGAAAACAAAAAAAGGCAGG
GGTTGCAATCCTAGTCTCTGATAAAACAGACTTTAAACCAACAAAGATCAAAAGAGACAAAGAAGGCCATTACATAATGGTAAAGGGATCAATT
CAACAAGAGGAGCTAACTATCCTAAATATTTATGCACCCAATACAGGAGCACCCAGATTCATAAAGCAAGTCCTCAGTGACCTACAAAGAGACT
TAGACTCCCACACATTAATAATGGGAGACTTTAACACCCCACTGTCAACATTAGACAGATCAACGAGACAGAAAGTCAACAAGGATACCCAGG
AATTGAACTCAGCTCTGCACCAAGCAGACCTAATAGACATCTACAGAACTCTCCACCCCAAATCAACAGAATATACATTTTTTTCAGCACCACA
CCACACCTATTCCAAAATTGACCACATAGTTGGAAGTAAAGCTCTCCTCAGCAAATGTAAAAGAACAGAAATTATAACAAACTATCTCTCAGACC
ACAGTGCAATCAAACTAGAACTCAGGATTAAGAATCTCACTCAAAGCCGCTCAACTACATGGAAACTGAACAACCTGCTCCTGAATGACTACT
GGGTACATAACGAAATGAAGGCAGAAATAAAGATGTTCTTTGAAACCAACGAGAACAAAGACACCACATACCAGAATCTCTGGGACGCATTCA
AAGCAGTGTGTAGAGGGAAATTTATAGCACTAAATGCCTACAAGAGAAAGCAGGAAAGATCCAAAATTGACACCCTAACATCACAATTAAAAGA
ACTAGAAAAGCAAGAGCAAACACATTCAAAAGCTAGCAGAAGGCAAGAAATAACTAAAATCAGAGCAGAACTGAAGGAAATAGAGACACAAAA
AACCCTTCAAAAAATCAATGAATCCAGGAGCTGGTTTTTTGAAAGGATCAACAAAATTGATAGACCGCTAGCAAGACTAATAAAGAAAAAAGA
GAGAAGAATCAAATAGACACAATAAAAAATGATAAAGGGGATATCACCACCGATCCCACAGAAATACAAACTACCATCAGAGAATACTACAAACA
CCTCTACGCAAATAAACTAGAAAATCTAGAAGAAATGGATACATTCCTCGACACATACACTCTCCCAAGACTAAACCAGGAAGAAGTTGAATCT
CTGAATAGACCAATAACAGGCTCTGAAATTGTGGCAATAATCAATAGTTTACCAACCAAAAAGAGTCCAGGACCAGATGGATTCACAGCCGAAT
TCTACCAGAGGTACAAGGAGGAACTGGTACCATTCCTTCTGAAACTATTCCAATCAATAGAAAAAGAGGGAATCCTCCCTAACTCATTTTATGA
GGCCAGCATCATTCTGATACCAAAGCCGGGCAGAGACACAACCAAAAAAGAGAATTTTAGACCAATATCCTTGATGAACATTGATGCAAAAATC
CTCAATAAAATACTGGCAAACCGAATCCAGCAGCACATCAAAAAGCTTATCCACCATGATCAAGTGGGCTTCATCCCTGGGATGCAAGGCTGG
TTCAATATACGCAAATCAATAAATGTAATCCAGCATATAAACAGAGCCAAAGACAAAAACCACATGATTATCTCAATAGATGCAGAAAAAGCCTTT
GACAAAATTCAACAACCCTTCATGCTAAAAACTCTCAATAAATTAGGTATTGATGGGACGTATTTCAAAATAATAAGAGCTATCTATGACAAACCC
ACAGCCAATATCATACTGAATGGGCAAAAACTGGAAGCATTCCCTTTGAAAACTGGCACAAGACAGGGATGCCCTCTCTCACCGCTCCTATTC
AACATAGTGTTGGAAGTTCTGGCCAGGGCAATCAGGCAGGAGAAGGAAATAAAGGGTATTCAATTAGGAAAAGAGGAAGTCAAATTGTCCCT
GTTTGCAGACGACATGATTGTTTATCTAGAAAACCCCATCGTCTCAGCCCAAAATCTCCTTAAGCTGATAAGCAACTTCAGCAAAGTCTCAGGA
TACAAAATCAATGTACAAAAATCACAAGCATTCTTATACACCAACAACAGACAAACAGAGAGCCAAATCATGGGTGAACTCCCATTCACAATTG
CTTCAAAGAGAATAAAATACCTAGGAATCCAACTTACAAGGGATGTGAAGGACCTCTTCAAGGAGAACTACAAACCACTGCTCAAGGAAATAAA
AGAGGACACAAACAAATGGAAGAACATTCCATGCTCATGGGTAGGAAGAATCAATATCGTGAAAATGGCCATACTGCCCAAGGTAATTTACAGA
TTCAATGCCATCCCCATCAAGCTACCAATGACTTTCTTCACAGAATTGGAAAAAACTACTTTAAAGTTCATATGGAACCAAAAAAGAGCCCGCAT
CGCCAAGTCAATCCTAAGCCAAAAGAACAAAGCTGGAGGCATCACACTACCTGACTTCAAACTATACTACAAGGCTACAGTAACCAAAACAGC
ATGGTACTGGTACCAAAACAGAGATATAGATCAATGGAACAGAACAGAGCCCTCAGAAATAATGCCGCATATCTACAACTATCTGATCTTTGACA
AACCTGAGAAAAACAAGCAATGGGGAAAGGATTCCCTATTTAATAAATGGTGCTGGGAAAACTGGCTAGCCATATGTAGAAAGCTGAAACTGG
ATCCCTTCCTTACACCTTATACAAAAATCAATTCAAGATGGATTAAAGATTTAAACGTTAGACCTAAAACCATAAAAACCCTAGAAGAAAACCTAG

GCATTACCATTCAGGACATAGGCGTGGGCAAGGACTTCATGTCCAAAACACCAAAAGCAATGGCAACAAAAGCCAAAATTGACAAATGGGATC
TAATTAAACTAAAGAGCTTCTGCACAGCAAAAGAAACTACCATCAGAGTGAACAGGCAACCTACAACATGGGAGAAAATTTTCGCAACCTACTC
ATCTGACAAAGGGCTAATATCCAGAATCTACAATGAACTCAAACAAATTTACAAGAAAAAAACAAACAACCCCATCAAAAAGTGGGCGAAGGAC
ATGAACAGACACTTCTCAAAAGAAGACATTTATGCAGCCAAAAAACACATGAAGAAATGCTCATCATCACTGGCCATCAGAGAAATGCAAATCA
AAACCACTATGAGATATCATCTCACACCAGTTAGAATGGCAATCATTAAAAAGTCAGGAAACAACAGGTGCTGGAGAGGATGTGGAGAAATAG
GAACACTTTTACACTGTTGGTGGGACTGTAAACTAGTTCAACCATTGTGGAAGTCAGTGTGGCGATTCCTCAGGGATCTAGAACTAGAAATAC
CATTTGACCCAGCCATCCCATTACTGGGTATATACCCAAAGGACTATAAATCATGCTGCTATAAAGACACATGCACACGTATGTTTATTGCGGCA
CTATTCACAATAGCAAAGACTTGGAACCAACCCAAATGTCCAACAATGATAGACTGGATTAAGAAAATGTGGCACATATACACCATGGAATACTA
TGCAGCCATAAAAAATGATGAGTTCATATCCTTTGTAGGGACATGGATGAAATTGGAAACCATCATTCTCAGTAAACTATCGCAAGAACAAAAAA
CCAAACACCGCATATTCTCACTCATAGGTGGGAATTGAACAATGAGATCACATGGACACAGGAAGGGGAATATCACACTCTGGGGACTGTGGT
GGGGTCGGGGGAGGGGGGAGGGATAGCATTGGGAGATATACCTAATGCTAGATGAC**ACG**TTAGTGGGTGCAGCGCACCAGCATGGCACATG
TATACATATGTAACTAACCTGCACAATGTGCACATGTACCCTAAAACTTAGAGTATAATAAAAAAAAAAAAAAAAAAAAAAAAAAAAAAAAAAAATAAA
TAAAAA

2.2 L1RP

It's the prototypical active L1 in modern humans with full-length LINE-1, ORF1p and ORF2p proteins (it's 3' UTR contains an ACA, making it an L1PA1)

Features	Regions (bp)
L1 5'UTR	1-906
ORF1 (L1RP)	907-1923
ORF2 (L1RP)	1987-5814
L1 3'UTR	5815-6019

GGGGGGAGGAGCCAAGATGGCCGAATAGGAACAGCTCCGGTCTACAGCTCCCAGCGTGAGCGACGCAGAAGACGGTGATTTCTGCATTTC
CATCTGAGGTACCGGGTTCATCTCACTAGGGAGTGCCAGACAGTGGGCGCAGGCCAGTGTGTGTGCGCACCGTGCGCGAGCCGAAGCAG
GGCGAGGCATTGCCTCACCTGGGAAGCGCAAGGGGTCAGGGAGTTCCCTTTCCGAGTCAAAGAAAGGGGTGACGGACGCACCTGGAAAAT
CGGGTCACTCCCACCCGAATATTGCGCTTTTCAGACCGGCTTAAGAAACGGCGCACCACGAGACTATATCCCACACCTGGCTCGGAGGGTC
CTACGCCCACGGAATCTCGCTGATTGCTAGCACAGCAGTCTGAGATCAAACTGCAAGGCGGCAACGAGGCTGGGGGAGGGGCGCCCGCCA
TTGCCCAGGCTTGCTTAGGTAAACAAAGCAGCAGGGAAGCTCGAACTGGGTGGAGCCCACCACAGCTCAAGGAGGCCTGCCTGCCTCTGT
AGGCTCCACCTCTGGGGGCAGGGCACAGACAAACAAAAAGACAGCAGTAACCTCTGCAGACTTAAGTGTCCCTGTCTGACAGCTTTGAAGA
GAGCAGTGGTTCTCCCAGCACGCAGCTGGAGATCTGAGAACGGGCAGACTGCCTCCTCAAGTGGGTCCCTGACCCCTGACCCCCGAGCAG
CCTAACTGGGAGGCACCCCCCAGCAGGGGCACACTGACACCTCACACGGCAGGGTATTCCAACAGACCTGCAGCTGAGGGTCCTGTCTGT
TAGAAGGAAAACTAACAACCAGAAAGGACATCTACACCGAAAACCCATCTGTACATCACCATCATCAAAGACCAAAAGTAGATAAAACCACAAA
GATGGGGAAAAAACAGAACAGAAAAACTGGAAACTCTAAAACGCAGAGCGCCTCTCCTCCTCCAAAGGAACGCAGTTCCTCACCAGCAACA
GAACAAAGCTGGATGGAGAATGATTTTGATGAGCTGAGAGAAGAAGGCTTCAGACGATCAAATTACTCTGAGCTACGGGAGGACATTCAAAC
CAAAGGCAAAGAAGTTGAAAACTTTGAAAAAAATTTAGAAGAATGTATAACTAGAATAACCAATACAGAGAAGTGCTTAAAGGAGCTGATGGAG
CTGAAAACCAAGGCTCGAGAACTACGTGAAGAATGCAGAAGCCTCAGGAGCCGATGCGATCAACTGGAAGAAAGGGTATCAGCAATGGAAG
ATGAAATGAATGAAATGAAGCGAGAAGGGAAGTTTAGAGAAAAAAGAATAAAAAGAAATGAGCAAAGCCTCCAAGAAATATGGGACTATGTGA
AAAGACCAAATCTACGTCTGATTGGTGTACCTGAAAGTGATGTGGAGAATGGAACCAAGTTGGAAAACACTCTGCAGGATATTATCCAGGAGA
ACTTCCCCAATCTAGCAAGGCAGGCCAACGTTCAGATTCAGGAAATACAGAGAACGCCACAAAGATACTCCTCGAGAAGAGCAACTCCAAGA
CACATAATTGTCAGATTCACCAAAGTTGAAATGAAGGAAAAAATGTTAAGGGCAGCCAGAGAGAAAGGTCGGGTTACCCTCAAAGGAAAGCC
CATCAGACTAACAGCGGATCTCTCGGCAGAAACCCTACAAGCCAGAAGAGAGTGGGGGCCAATATTCAACATTCTTAAAGAAAAGAATTTTCA
ACCCAGAATTTCATATCCAGCCAAACTAAGCTTCATAAGTGAAGGAGAAATAAAATACTTTATAGACAAGCAAATGTTGAGAGATTTTGTCACCA
CCAGGCCTGCCCTAAAAGAGCTCCTGAAGGAAGCGCTAAACATGGAAAGGAACAACCGGTACCAGCCGCTGCAAAATCATGCCAAAATGTAA
AGACCATCAAGACTAGGAAGAAACTGCATCAACTAATGAGCAAAATCACCAGCTAACATCATAATGACAGGATCAACTTCACACATAACAATATT
AACTTTAAATATAAATGGACTAAATTCTGCAATTAAAAGACACAGACTGGCAAGTTGGATAAAGAGTCAAGACCCATCAGTGTGCTGTATTCAGG
AAACCCATCTCACGTGCAGAGACACACATAGGCTCAAAATAAAAGGATGGAGGAAGATCTACCAAGCCAATGGAAAACAAAAAAAGGCAGGG
GTTGCAATCCTAGTCTCTGATAAAACAGACTTTAAACCAACAAAGATCAAAAGAGACAAAGAAGGCCATTACATAATGGTAAAGGGATCAATTC
AACAAGAGGAGCTAACTATCCTAAATATTTATGCACCCAATACAGGAGCACCCAGATTCATAAAGCAAGTCCTCAGTGACCTACAAAGAGACTT
AGACTCCCACACATTAATAATGGGAGACTTTAACACCCCACTGTCAACATTAGACAGATCAACGAGACAGAAAGTCAACAAGGATACCCAGGA
ATTGAACTCAGCTCTGCACCAAGCAGACCTAATAGACATCTACAGAACTCTCCACCCCAAATCAACAGAATATACATTTTTTTCAGCACCACAC
CACACCTATTCCAAAATTGACCACATAGTTGGAAGTAAAGCTCTCCTCAGCAAATGTAAAAGAACAGAAATTATAACAAACTATCTCTCAGACCA
CAGTGCAATCAAACTAGAACTCAGGATTAAGAATCTCACTCAAAGCCGCTCAACTACATGGAAACTGAACAACCTGCTCCTGAATGACTACTG
GGTACATAACGAAATGAAGGCAGAAATAAAGATGTTCTTTGAAACCAACGAGAACAAAGACACCACATACCAGAATCTCTGGGACGCATTCAA
AGCAGTGTGTAGAGGGAAATTTATAGCACTAAATGCCTACAAGAGAAAGCAGGAAAGATCCAAAATTGACACCCTAACATCACAATTAAAAGAA
CTAGAAAAGCAAGAGCAAACACATTCAAAAGCTAGCAGAAGGCAAGAAATAACTAAAATCAGAGCAGAACTGAAGGAAATAGAGACACAAAAA
ACCCTTCAAAAAATCAATGAATCCAGGAGCTGGTTTTTTGAAAGGATCAACAAAATTGATAGACCGCTAGCAAGACTAATAAAGAAAAAAGAG
AGAAGAATCAAATAGACACAATAAAAAATGATAAAGGGGATATCACCACCGATCCCACAGAAATACAAACTACCATCAGAGAATACTACAAACAC
CTCTACGCAAATAAACTAGAAAATCTAGAAGAAATGGATACATTCCTCGACACATACACTCTCCCAAGACTAAACCAGGAAGAAGTTGAATCTC
TGAATCGACCAATAACAGGCTCTGAAATTGTGGCAATAATCAATAGTTTACCAACCAAAAAGAGTCCAGGACCAGATGGATTCACAGCCGAATT
CTACCAGAGGTACAAGGAGGAACTGGTACCATTCCTTCTGAAACTATTCCAATCAATAGAAAAAGAGGGAATCCTCCCTAACTCATTTTATGAG
GCCAGCATCATTCTGATACCAAAGCCGGGCAGAGACACAACCAAAAAAGAGAATTTTAGACCAATATCCTTGATGAACATTGATGCAAAAATCC
TCAATAAAATACTGGCAAACCGAATCCAGCAGCACATCAAAAAGCTTATCCACCATGATCAAGTGGGCTTCATCCCTGGGATGCAAGGCTGGT
TCAATATACGCAAATCAATAAATGTAATCCAGCATATAAACAGAGCCAAAGACAAAAACCACATGATTATCTCAATAGATGCAGAAAAAGCCTTTG

ACAAAATTCAACAACCCTTCATGCTAAAAACTCTCAATAAATTAGGTATTGATGGGACGTATTTCAAAATAATAAGAGCTATCTATGACAAACCCA
CAGCCAATATCATACTGAATGGGCAAAAACTGGAAGCATTCCCTTTGAAAACCGGCACAAGACAGGGATGCCCTCTCTCACCGCTCCTATTCA
ACATAGTGTTGGAAGTTCTGGCCAGGGCAATCAGGCAGGAGAAGGAAATAAAGGGTATTCAATTAGGAAAAGAGGAAGTCAAATTGTCCCTG
TTTGCAGACGACATGATTGTTTATCTAGAAAACCCCATCGTCTCAGCCCAAAATCTCCTTAAGCTGATAAGCAACTTCAGCAAAGTCTCAGGAT
ACAAAATCAATGTACAAAAATCACAAGCATTCTTATACACCAACAACAGACAAACAGAGAGCCAAATCATGGGTGAACTCCCATTCACAATTGC
TTCAAAGAGAATAAAATACCTAGGAATCCAACTTACAAGGGATGTGAAGGACCTCTTCAAGGAGAACTACAAACCACTGCTCAAGGAAATAAAA
GAGGAGACAAACAAATGGAAGAACATTCCATGCTCATGGGTAGGAAGAATCAATATCGTGAAAATGGCCATACTGCCCAAGGTAATTTACAGAT
TCAATGCCATCCCCATCAAGCTACCAATGACTTTCTTCACAGAATTGGAAAAAACTACTTTAAAGTTCATATGGAACCAAAAAAGAGCCCGCATT
GCCAAGTCAATCCTAAGCCAAAAGAACAAAGCTGGAGGCATCACACTACCTGACTTCAAACTATACTACAAGGCTACAGTAACCAAAACAGCA
TGGTACTGGTACCAAAACAGAGATATAGATCAATGGAACAGAACAGAGCCCTCAGAAATAATGCCGCATATCTACAACTATCTGATCTTTGACAA
ACCTGAGAAAAACAAGCAATGGGGAAAGGATTCCCTATTTAATAAATGGTGCTGGGAAAACTGGCTAGCCATATGTAGAAAGCTGAAACTGGA
TCCCTTCCTTACACCTTATACAAAAATCAATTCAAGATGGATTAAAGATTTAAACGTTAAACCTAAAACCATAAAAACCCTAGAAGAAAACCTAGG
CATTACCATTCAGGACATAGGCGTGGGCAAGGACTTCATGTCCAAAACACCAAAAGCAATGGCAACAAAAGACAAAATTGACAAATGGGATCT
AATTAAACTAAAGAGCTTCTGCACAGCAAAAGAAACTACCATCAGAGTGAACAGGCAACCTACAACATGGGAGAAAATTTTTGCAACCTACTCA
TCTGACAAAGGGCTAATATCCAGAATCTACAATGAACTCAAACAAATTTACAAGAAAAAAACAAACAACCCCATCAAAAAGTGGGCGAAGGACA
TGAACAGACACTTCTCAAAAGAAGACATTTATGCAGCCAAAAAACACATGAAGAAATGCTCATCATCACTGGCCATCAGAGAAATGCAAATCAA
AACCACTATGAGATATCATCTCACACCAGTTAGAATGGCAATCATTAAAAAGTCAGGAAACAACAGGTGCTGGAGAGGATGCGGAGAAATAGG
AACACTTTTACACTGTTGGTGGGACTGTAAACTAGTTCAACCATTGTGGAAGTCAGTGTGGCGATTCCTCAGGGATCTAGAACTAGAAATACC
ATTTGACCCAGCCATCCCATTACTGGGTATATACCCAAATGAGTATAAATCATGCTGCTATAAAGACACATGCACACGTATGTTTATTGCGGCACT
ATTCACAATAGCAAAGACTTGGAACCAACCCAAATGTCCAACAATGATAGACTGGATTAAGAAAATGTGGCACATATACACCATGGAATACTATG
CAGCCATAAAAAATGATGAGTTCATATCCTTTGTAGGGACATGGATGAAATTGGAAACCATCATTCTCAGTAAACTATCGCAAGAACAAAAAACC
AAACACCGCATATTCTCACTCATAGGTGGGAATTGAACAATGAGATCACATGGACACAGGAAGGGGAATATCACACTCTGGGGACTGTGGTG
GGGTCGGGGGAGGGGGGAGGGATAGCATTGGGAGATATACCTAATGCTAGATGAC**ACA**TTAGTGGGTGCAGCGCACCAGCATGGCACATGT
ATACATATGTAACTAACCTGCACAATGTGCACATGTACCCTAAAACTTAGAGTATAAT

Supplementary Sequence 3. DNA sequences of pU6-pegRNAs, lentiCRISPR v2 plasmid, pCMV-PE2 plasmid, pCMV-BEs (BE2/BE3/BE4-Gam/AncBE4max/ABE8e) plasmids used for insertion sources analysis.

3.1.1 pU6-pegRNA (for *MYC* +2-4AAAdel) (497 bp)

Features	Start	end
pU6	82	330
pegRNA	331	455
Spacer	331	350
RT (+2-4AAAdel)	427	442
PBS	443	455

Gacgtcgctagctgtacaaaaaagcaggctttaaaggaaccaattcagtcgactggatccggtaccaaggtcgggcaggaagagggcctatttcccatgattccttcatatttgc
atatacgatacaaggctgttagagagataattagaattaatttgactgtaaacacaaagatattagtacaaaatacgtgacgtagaaagtaataatttcttgggtagtttgcagtttta
aaattatgttttaaaatggactatcatatgcttaccgtaacttgaaagtatttcgatttcttggctttatatatcttgtggaaaggacgaaa**CACC**GACTGTCCAAAGGGGG
TGAAGTTTT**AGAG**CTAGAAATAGCAA**GTTA**AAATAAGGCTAGTCCGTTATCAACTTGAAAAAGTGGCACCGAGTCG**GTGC**A
TAAAGGGAGCACCCCACCCCCTTTGGACttttttaagcttgggccgctcgaggtacctctctacatatg

3.1.2 pU6-pegRNA (for *MYC* +5GtoC) (496 bp)

Features	Start	end
pU6	82	330
pegRNA	331	455
Spacer	331	350
RT (5GtoC)	427	441
PBS	442	454

Gacgtcgctagctgtacaaaaaagcaggctttaaaggaaccaattcagtcgactggatccggtaccaaggtcgggcaggaagagggcctatttcccatgattccttcatatttgcatatacgatacaaggctg
ttagagagataattagaattaatttgactgtaaacacaaagatattagtacaaaatacgtgacgtagaaagtaataatttcttgggtagtttgcagttttaaaattatgttttaaaatggactatcatatgcttaccgta
acttgaaagtatttcgatttcttggctttatatatcttgtggaaaggacgaaa**CACC**GACTGTCCAAAGGGGGTGAAGTTTT**AGAG**CTAGAAATAGCAA**GTTA**AAATAAGG
CTAGTCCGTTATCAACTTGAAAAAGTGGCACCGAGTCG**GTGCAGGGAGCACCGTTTCACCCCCTTTGGAC**ttttttaagcttgggccgctcgaggtacctctc
tacatatg

3.1.3 pU6-pegRNA (for *FANCF* +5GtoT) (500 bp)

Features	Start	end
pU6	82	330
pegRNA	331	455
Spacer	331	350
RT (5GtoT)	427	443
PBS	444	458

Gacgtcgctagctgtacaaaaaagcaggctttaaaggaaccaattcagtcgactggatccggtaccaaggtcgggcaggaagagggcctatttcccatgattccttcatatttgcatatacgatacaaggctg
ttagagagataattagaattaatttgactgtaaacacaaagatattagtacaaaatacgtgacgtagaaagtaataatttcttgggtagtttgcagttttaaaattatgttttaaaatggactatcatatgcttaccgta
acttgaaagtatttcgatttcttggctttatatatcttgtggaaaggacgaaa**CACC**GGAATCCCTTCTGCAGCACCGTTTT**AGAG**CTAGAAATAGCAA**GTTA**AAATAAGG
CTAGTCCGTTATCAACTTGAAAAAGTGGCACCGAGTCG**GTGCGGAAAAGCGATCAAGGTGCTGCAGAAGGGATT**ttttttaagcttgggccgctcgaggt
acctctctacatatg

3.2 lentiCRISPR v2 plasmid

can be found in https://www.addgene.org/52961/

3.3 pCMV-PE2 plasmid

can be found in https://www.addgene.org/132775/

3.4 pCMV-BEs plasmids

pCMV-BE2 (Addgene plasmid #73020), pCMV-BE3 (Addgene plasmid #73021), pCMV-BE4-Gam (Addgene plasmid #100806), pCMV-AncBE4max (Addgene plasmid #112094) and pCMV-ABE8e (Addgene plasmid #138489) can be found in https://www.addgene.org/.

Supplementary Sequence 4. Example L1-ORFeus insertional sequences obtained by amplicon sequencing at the PE3 (MYC +2-4AAA del) editing site in HEK293T cells expressing L1-ORFeus.

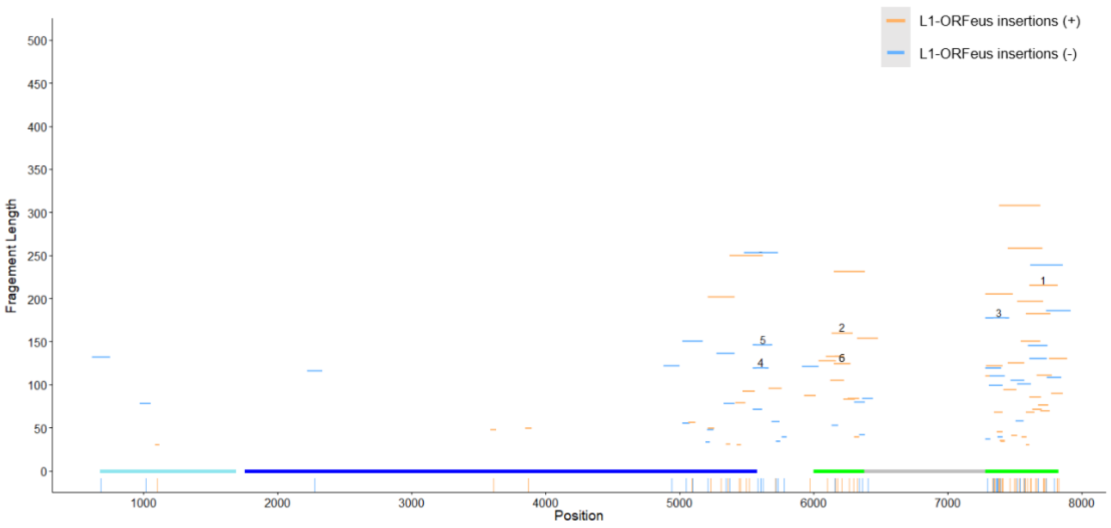

Sequence color annotation: MYC locus pegRNA editing site Microhomology L1-ORFeus insertions (+) L1-ORFeus insertions (-)

Example #1 (number as annotated above)

>M00851:457:000000000-CK7JC:1:1101:29091:14400 (pair end)

GCTGGTTCACTAAGTGCGTCTCCGAGATAGCAGGGGACTGTCCAAAGGGG**TGG**GGTGCTCCCTTTA**TGCAC**GCCGTAGGTCAGGGTGGTCACGAG GGTGGGCCAGGGCACGGGCAGCTTGCCGGTGGTGCAGATGAACTTCAGGGTCAGCTTGCCGTAGGTGGCATCGCCCTCGCCCTCGCCGGACACGC TGAACTTGTGGCCGTTTACGTCGCCGTCCAGCTCGACCAGGATGGGCACCACCCCGGTGAACAGCTCCTCGCCCTTGCTCACCATGGTG**GC**TTTAGG GGATAGCTCTGCAAGGGGAGAG

Example #2

>M00851:457:000000000-CK7JC:1:1108:19871:27977 (pair end)

GCTGGTTCACTAAGTGCGTCTCCGAGATAGCAGGGGACTGTCCAAAGGGGG**TGG**GGTGCTCCCTTTATGCACCGACTCGGT**GCC**GCCAAGCTCTTC AGCAATATCACGGGTAGCCAACGCTATGTCCTGATAGCGGTCGGCCGCTTTACTTGTACAGCTCGTCCATGCCGAGAGTGATCCCGGCGGCGGTCA CGAACTCCAGCAGGACCATGTGATCGCGCTTCTCGTTGGGGTCTTT**GCT**TTAGGGGATAGCTCTGCAAGGGGAGAG

Example #3

>M00851:457:000000000-CK7JC:1:2104:4552:9611 (pair end)

TTGACTTGGCTGGTTCACTAAGTGCGTCTCCGAGATAGCAGGGGACTGTCCAAAGGGGG**TGGGGTG**AACCGCATCGAGCTGAAGGGCATCGACTT CAAGGAGGACGGCAACATCCTGGGGCACAAGCTGGAGTACAACTACAACAGCCACAACGTCTATATCATGGCCGACAAGCAGAAGAACGGCATCA AGGTGAACTTCAAGATCCGCCACAACATCGAGGACGGCAGCGTGCA**GCT**TTAGGGGATAGCTCTGCAAGGGGAGAG

Example #4

>M00851:457:000000000-CK7JC:1:1103:13770:4340 (pair end)

GCTGGTTCACTAAGTTCGTCTCCGAGATAGCAGGGGACTGTCCAAAGGGGG**TGG**GGTGCTCCCTTTATGCACCGACTCGGTGCCACTTTTTCAAGT TGAT**AACGGA**ACTTCCAGGGGCCCCTGGAACAGAACTTCCAGGTGATGGTGATGGTGATGGTGATGGCTGCCGCCGCCGCTGCCGCCGCCGTTGC CGCCGATCAGGCTGAAGATGCGGT**GCTT**TAGGGGATAGCTCTGCAAGGGGAGAG

Example #5 (not edited)

>M00851:457:000000000-CK7JC:1:2102:28880:17158 (pair end)

GCTGGTTCACTAAGTGCGTCTCCGAGATAGCAGGGGACTGTCCAAAGGGGGTGAAAGGGTGCTCCCTTTATTCCCCCACCA**AG**CTTCTGTTCCTTA AGGGGCCCCTGGAACAGAACTTCCAGGGGCCCCTGGAACAGAACTTCCAGGTGATGGTGATGGTGATGGTGATGGCTGCCGCCGCCGCTGCCGCC GCCGTTGCCGCCGATCAGGCTGAAGATGCGGT**GCTT**TAGGGGATAGCTCTGCAAGGGGAGAG

Example #6 (editing sequence deleted)

>M00851:457:000000000-CK7JC:1:2111:14788:20778 (pair end)

TTGACTTGGCTGGTTCACTAAGTGCGTCTCCGAGAT**AGCA**ATATCACGGGTAGCCAACGCTATGTCCTGATAGCGGTCGGCCGCTTTACTTGTACAG CTCGTCCATGCCGAGAGTGATCCCGGCGGCGGTCACGAACTCCAGCAGGACCATGTGATCGC**GCTT**TAGGGGATAGCTCTGCAAGGGGAGAG

Supplementary Sequence 5. L1-ORFeus insertions bridging the intron of GFP obtained by amplicon sequencing at the PE3 (MYC +2-4AAA del) editing site in HEK293T cells expressing L1-ORFeus.

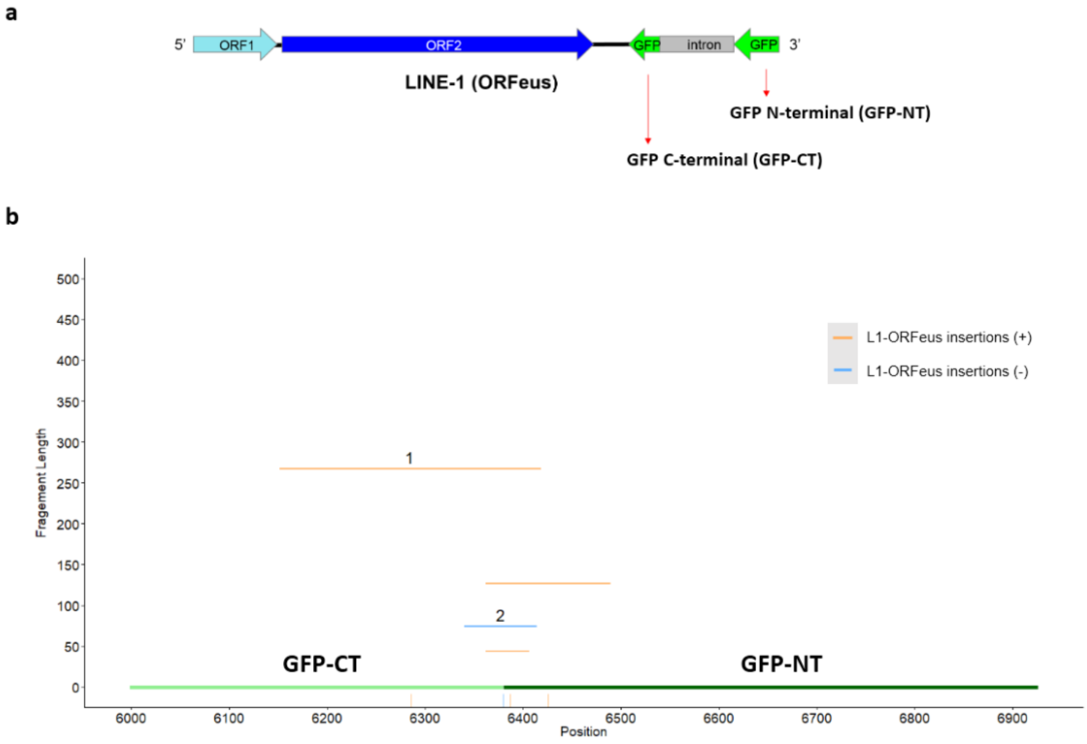

Example #1 (edits deleted)

>M00851:457:000000000-CK7JC:1:2106:17702:11039 (pair end)

GCTGGTTCACTAAGTGCGTCTCCGAGAT**AGCA**ATATCACGGGTAGCCAACGCTATGTCCTGATAGCGGTCGGCCGCTTTACTTGTACAGCTCGTCCATGC CGAGAGTGATCCCGGCGGCGGTCACGAACTCCAGCAGGACCATGTGATCGCGCTTCTCGTTGGGGTCTTTGCTCAGGGCGGACTGGGTGCTCAGGTAGT GGTTGTCGGGCAGCAGCACGGGGCCGTCGCCGATGGGGGTGTTCTGCTGGTAGTGGTCGGCCAGCTGCACGCTGCCGTCCTCGATGTTGTGGCGGAT**CT** **T**TATTCCCCCACCAAGACCACCCAGCCGCTTTAGGGGATAGCTCTGCAAGGGGAGAG

Sequence color annotation: MYC locus Microhomology L1-ORFeus insertions (+): GFP-CT GFP-NT

Example #2

>M00851:457:000000000-CK7JC:1:1101:17292:2963 (pair end)

GCTGGTTCACTAAGTGCGTCTCCGAGATAGCAGGGGACTGTCCAAAGGGGG**TGG**GGTGCTCCCTTTATTCCGCCACAACATCGAGGACGGCAGCGTGCA GCTGGCCGACCACTACCAGCAGAACACCCCCATCGGCGACG**GC**TTTAGGGGATAGCTCTGCAAGGGGAGAG

Sequence color annotation: MYC locus pegRNA editing site Microhomology L1-ORFeus insertions (-): GFP-NT GFP-CT

Supplementary Sequence 6. L1-ORFeus insertions bridging the intron of GFP obtained by amplicon sequencing at the MYC CRISPR/Cas9 target site in HEK293T cells expressing L1-ORFeus.

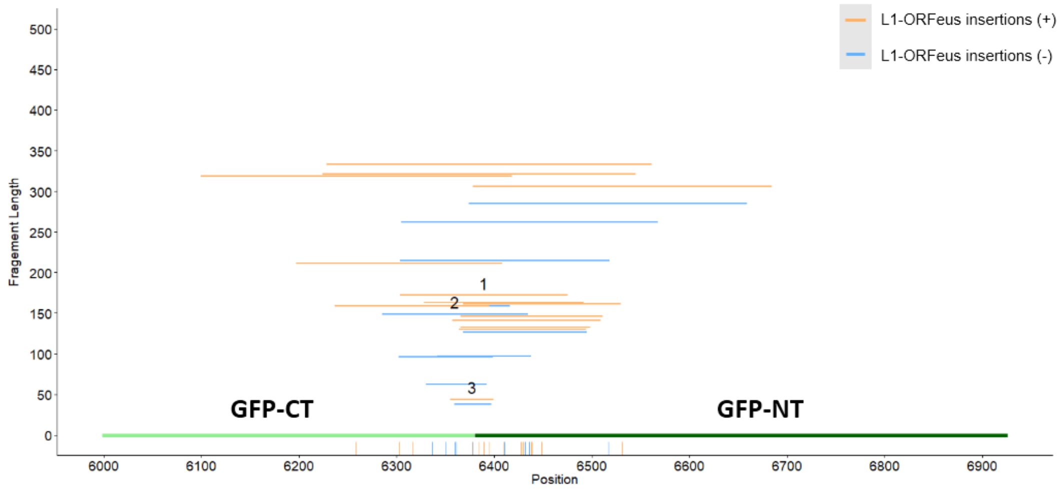

Example #1

>M00851:382:000000000-CF5PB:1:2103:18157:2519 (pair end)

GATCGCGCGGTGCTGGTTCACTAAGTGCGTCTCCGAGATAGCAGGGGACTGTCCAAAG**GGG**TGCTCAGGTAGTGGTTGTCGGGCAGCAGCACGG
GGCCGTCGCCGATGGGGGTGTTCTGCTGGTAGTGGTCGGCCAGCTGCACGCTGCCGTCCTCGATGTTGTGGCGGATCTTGAAGTTCACCTTGATGC
CGTTCTTCTGCTTGTCGGCCATGATATAGACGTTGTG**GCT**CCCTTTATTCCCCCACCAAGACCACCCAGCCGCTTTAGGGGATAGCTCTGCAAGGGG
AGAGCTCTC

Sequence color annotation: MYC locus Microhomology L1-ORFeus insertions (+): GFP-CT GFP-NT

Example #2

>M00701:747:000000000-CFM9G:1:2110:14086:2427 (pair end)

CAGCTGGTTCACTAAGTGCGTCTCCGAGATAGCAGGGGACTGTCCAAA**GG**CATCAAGGTGAACTTCAAGATCCGCCACAACATCGAGGACGGCAG
CGTGCAGCTGGCTGACCACTACCAGCAGAACACCCCCATCGGCGACGGCCCCGTGCTGCTGCCCGACAACCACTACCTGAGCACCCAGTCCGCCCT
GAGC**AAAG**GGTGCTCCCTTTATTCCCCCACCAAGACCACCCAGCCGCTTTAGGGGATAGCTCTGCAAGGGGAGAGCTC

Sequence color annotation: MYC locus Microhomology L1-ORFeus insertions (-): GFP-NT GFP-CT

Example #3

>M00701:747:000000000-CFM9G:1:1104:22072:21267 (pair end)

CAGCTGGTTCACTAAGTGCGTCTCCGAGATAGCAGGGGACTGTCCAAAG**GGGG**GTGTTCTGCTGGGAGTGGTCGGCCAGCTGCACGCTGCCGTCC
TTCCCTTTATTCCCCCACCAAGACCACCCAGCCGCTTTAGGGGATAGCTCTGCAAGGGGAGAGCTC

Sequence color annotation: MYC locus Microhomology L1-ORFeus insertions (+): GFP-CT GFP-NT

Supplementary Sequence 7. L1-ORFeus insertions bridging the intron of GFP obtained by amplicon sequencing at the RAG1 CRISPR/Cas9 target site in HEK293T cells expressing L1-ORFeus.

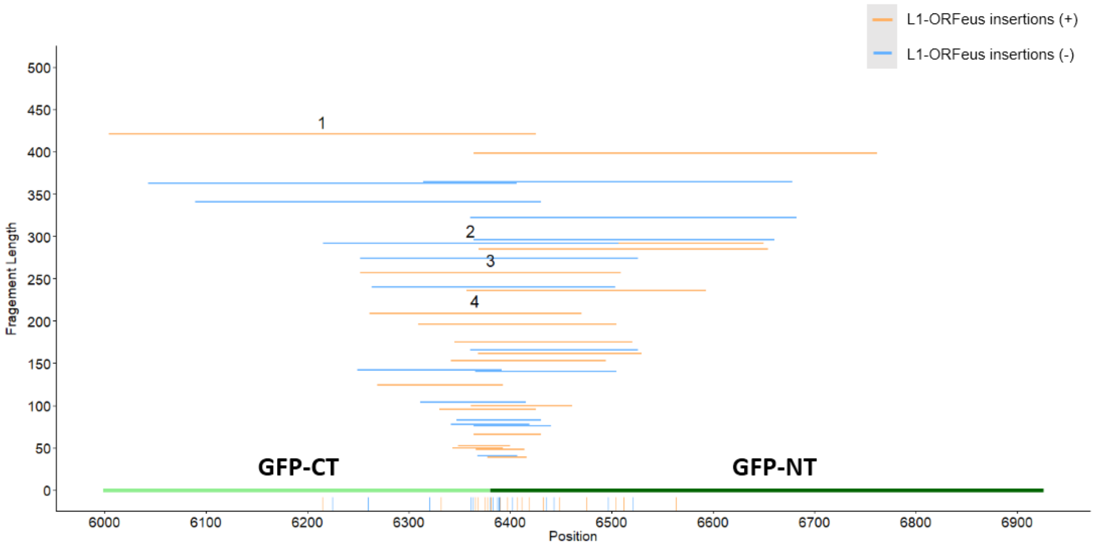

Example #1

>M00701:747:000000000-CFM9G:1:1105:8807:6910 (pair end)

ACGCGCTGCGTAAGATACATCAGTGGGATATTGATATTGGTCTTAATATGACTTGTTTTCATTGTTCTCAGGTACCTCAGCCAGCATGGCAGCCTCTT
TCCCACC**CACCT**GTGCGTTTTATTCTGTCTTTTTATTGCCGATCCCCTCAGAAGAACTCGTCAAGAAGGCGATAGAAGGCGATGCGCTGCGAATCGG
GAGCGGCGATACCGTAAAGCACGAGGAAGCGGTCAGCCCATTCGCCGCCAAGCTCTTCAGCAATATCACGGGTAGCCAACGCTATGTCCTGATAG
CGGTCGGCCGCTTTACTTGTACAGCTCGTCCATGCCGAGAGTGATCCCGGCGGCGGTCACGAACTCCAGCAGGACCATGTGATCGCGCTGCTCGTT
GAGGTCTTTGCTCAGGGCGGACTGGGTGCTCAGGTAGTGGTTGTCGGGCAGCAGCACGGGGCCGTCGCCGATGGGGGTGTTCTGCTGGTAGTGG
TCGGCCAGCTGCACGCTGCCGTCCTCGATGTTGTGGCGGATCTTGAAGTTC**AC**TCAGTTCTGCCCCAGATGAAATTCAGCACCCACATATTAAATTTT
CAGAATGGAAATTTAAGCTGTTCCGGGTGAGATCCT

Sequence color annotation: RAG1 locus Microhomology L1-ORFeus insertions (+): GFP-CT GFP-NT

Example #2

>M00701:747:000000000-CFM9G:1:1113:12595:14133 (pair end)

ACGCGCTGCGTAAGATACATCAGTGGGATATTGATATTGGTCTTAATATGACTTGTTTTCATTGTTCTCAGGTACCTCAGCCAGCATGGCAGCCTCTT
TCCCACC**CATCCTGGG**GCACAAGCTGGAGTACAACTACAACAGCCACAACGTCTATATCATGGCCGACAAGCAGAAGAACGGCATCAAGGTGAAC
TTCAAGATCCGCCACAACATCGAGGACGGCAGCGTGCAGCTGGCCGACCACTACCAGCAGAACACCCCCATCGGCGACGGCCCCGTGCTGCTGCC
CGACAACCACTACCTGAGCACCCAGTCCGCCCTGAGCAAAGACCCCAACGAGAAGCGCGATCACATGGTCCTGCTGGAGTTCGTGACCGCCGCCG
GGATCACTCTCGGCAT**GGAC**TCAGTTCTGCCCCAGATGAAATTCAGCACCCACATATTAAATTTTCAGAATGGAAATTTAAGCTGTTCCGGGTGAGA
TCCTCTG

Sequence color annotation: RAG1 locus Microhomology L1-ORFeus insertions (-): GFP-NT GFP-CT

Example #3

>M00701:748:000000000-CFM62:1:2101:12456:25523 (pair end)

ACGCGCTGCGTAAGATACATCAGTGGGATATTGATATTGGTCTTAATATGACTTGTTTTCATTGTTCTCAGGTACCTCAGCCAGCATGGCAGCCTCTT
TCCCAC**CCA**GCAGGACCATGTGATCGCGCTTCTCGTTGGGGTCTTTGCTCAGGGCGGACTGGGTGCTCAGGTAGTGGTTGTCGGGCAGCAGCACG
GGGCCGTCGCCGATGGGGGTGTTCTGCTGGTAGTGGTCGGCCAGCTGCACGCTGCCGTCCTCGATGTTGTGGCGGATCTTGAAGTTCACCTTGATG
CCGTTCTTCTGCTTGTCGGCCATGATATAGACGTTGTGGCTGTTGTAGTTGTACTCCAGCTTGTGCCCCAGGATG**TTG**GGACTCAGTTCTGCCCCAG
ATGAAATTCAGCACCCACATATTAAATTTTCAGAATGGAAATTTAAGCTGTTCCGGGTGAGATCCTCTGA

Example #4

>M01559:236:000000000-CHDLT:1:2101:16442:20380 (pair end)

ACGCGCTGCGTAAGATACATCAGTGGGATATTGATATTGGTCTTAATATGACTTGTTTTCATTGTTCTCAGGTACCTCAGCCAGCATGGCAGCCTCTT
TCCCAC**CCA**TGTGATCGCGCTTCTCGTTGGGGTCTTTGCTCAGGGCGGACTGGGTGCTCAGGTAGTGGTTGTCGGGCAGCAGCACGGGGCCGTCG
CCGATGGGGGTGTTCTGCTGGTAGTGGTCGGCCAGCTGCACGCTGCCGTCCTCGATGTTGTGGCGGATCTTGAAGTTCACCTTGATGCCGTTCTTCT
GCTTGTCGGCCATGATATAGACGTTG**TGG**GACTCAGTTCTGCCCCAGATGAAATTCAGCACCCACATATTAAATTTTCAGAATGGAAATTTAAGCTG
TTCCGGGTGAGATCCT

Supplementary Sequence 8. L1-ORFeus insertion bridging the intron of GFP obtained by amplicon sequencing at the CCR5 CRISPR/Cas9 target site in HEK293T cells expressing L1-ORFeus.

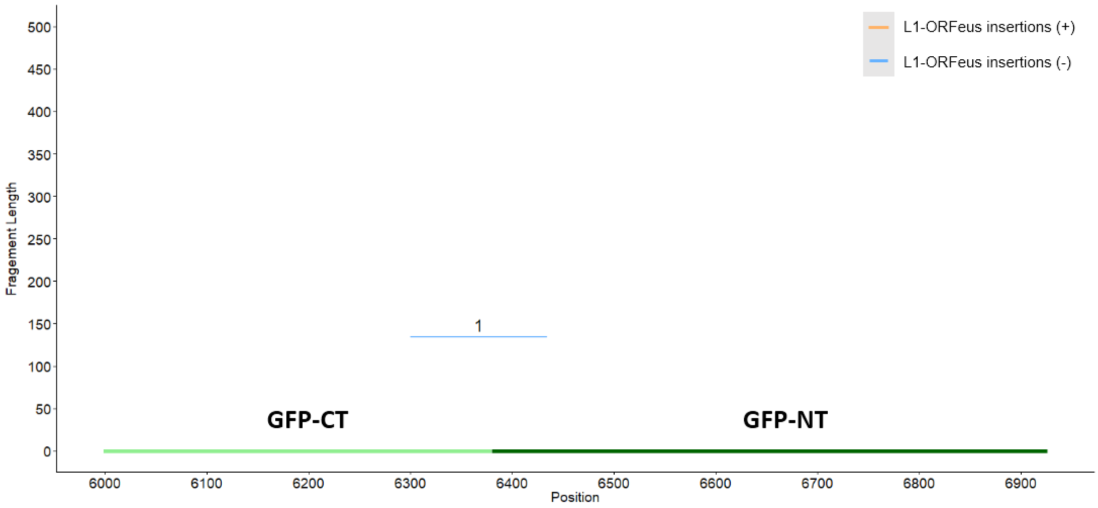

Example #1

>M01559:337:000000000-JBGB2:1:2108:10959:9836 (pair end)

ATGATGTACCAGGAATCATCTTTACCAGATCTCAAAAAGAAGGTCTTCATTACACCTGCAGCTCTCATTTTCCATA**CA**TCAAGGTGAACTTCAAG
ATCCGCCACAACATCGAGGACGGCAGCGTGCAGCTGGCCGACCACTACCAGCAGAACACCCCCATCGGCGACGGCCCCGTGCTGCTGCCCGAC
AACCACTACCTGAGCACC**CAGT**ATCAATTCTGGAAGAATTTCCAGACATTAAAGATAGTCATCTTGGGGCTGGTCCTG

Sequence color annotation: CCR5 locus Microhomology L1-ORFeus insertions (-): GFP-NT GFP-CT

Supplementary Sequence 9. L1-ORFeus insertions bridging the intron of GFP obtained by amplicon sequencing at the MYC CRISPR/Cas9 target site in HeLa cells expressing L1-ORFeus.

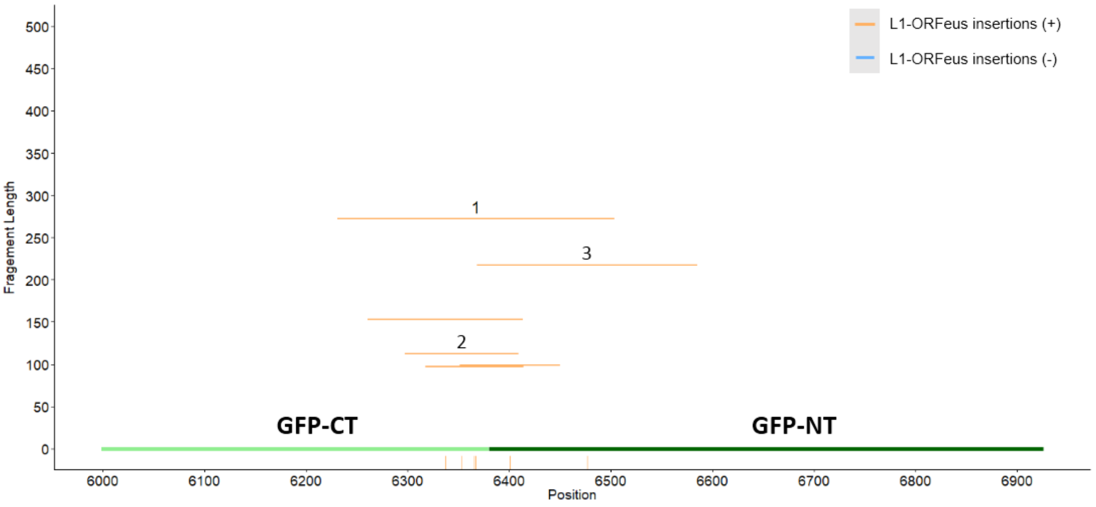

Sequence color annotation: MYC locus Microhomology L1-ORFeus insertions (+): GFP-CT GFP-NT

Example #1

>M00701:936:000000000-JT8MN:1:2109:15161:26467 (pair end)
TGCTGCTGGTTCACTAAGTGCGTCTCCGAGATAGCAGGGGACTGTCCAAAGGGGGTCGGCGGCGGTCACGAACTCCAGCAGGACCATGTGAT
CGCGCTTCTCGTTGGGGTCTTTGCTCAGGGCGGACTGGGTGCTCAGGTAGTGGTTGTCGGGCAGCAGCACGGGGCCGTCGCCGATGGGGGT
GTTCTGCTGGTAGTGGTCGGCCAGCTGCACGCTGCCGTCCTCGATGTTGTGGCGGATCTTGAAGTTCACCTTGATGCAGTTCTTCTGCTTGTCG
GCCATGATATAGACGTTGTGGCTGTTGTAGTTGTACTCCAGCTTGTGCCCCTGAAAGGGTGCTCCCTTTATTCCCCCACCAAGACCACCCAGCC
GCTTTAGGGGATAGCTCTGCAAGGGGAGAGCTC

Example #2

>M00701:936:000000000-JT8MN:1:1105:6364:9728 (pair end)
TGCTGCTGGTTCACTAAGTGCGTCTCCGAGATAGCAGGGGACTGTCCAAAGCGGACTGGGTGCTCAGGTAGTGGTTGTCGGGCAGCAGCACG
GGGCCGTCGCCGATGGGGGTGTTCTGCTGGTAGTGGTCGGCCAGCTGCACGCTGCCGTCCTCGATGTTGTGAAAGGGTGCTCCCTTTATTCCC
CCACCAAGACCACCCAGCCGCTTTAGGGGATAGCTCTGCAAGGGGAGAGCTC

Example #3

>M00701:936:000000000-JT8MN:1:1104:11116:4812 (pair end)
GATCGCGCGGTGCTGGTTCACTAAGTGCGTCTCCGAGATAGCAGGGGACTGTCCAAAGGTAGTGGTCGGCCAGCTGCACGCTGCCGTCCTCG
ATGTTGTGGCGGATCTTGAAGTTCACCTTGATGCCGTTCTTCTGCTTGTCGGCCATGATATAGACGTTGTGGCTGTTGTAGTTGTACTCCAGCTT
GTGCCCCAGGATGTTGCCGTCCTCCTTGAAGTCGATGCCCTTCAGCTCGATGCGGTTCACCAGGGTGTCGCCCTCGAACTTCACCTCGGTTTATT
CCCCCACCAAGACCACCCAGCCGCTTTAGGGGATAGCTCTGCAAGGGGAGAGCTC

Supplementary Sequence 10. L1-ORFeus insertional sequence obtained by amplicon sequencing at the CCR5 CRISPR/Cas9 target site in HEK293T cells expressing L1-RTm.

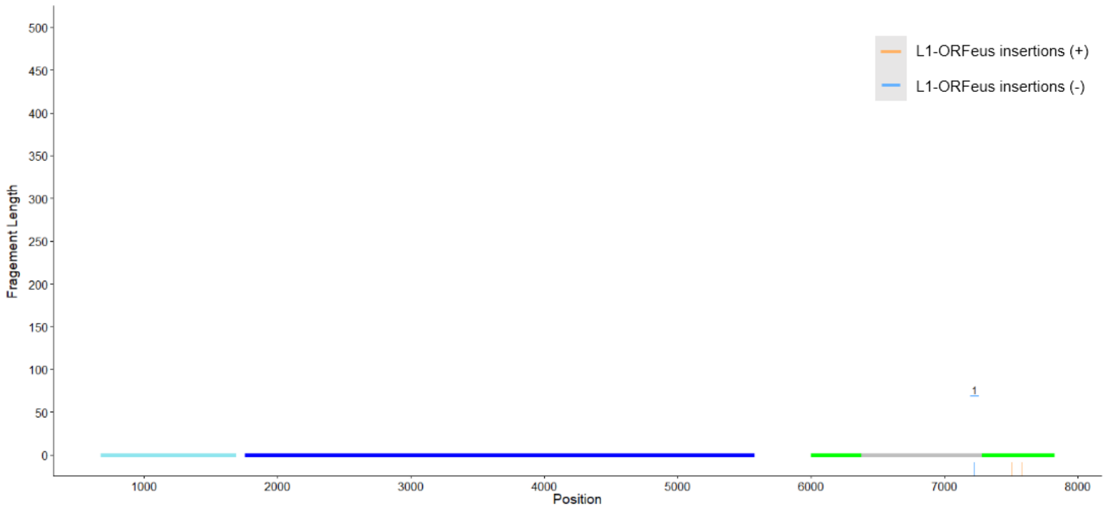

Example #1

>M01559:337:000000000-JBGB2:1:1110:23520:10454 (pair end)

GATCCAGGAATCATCTTTACCAGATCTCAAAAAGAAGGTCTTCATTACACCTGCAGCTCTCATTTTCCATATAAAGATGAACCCATAGTG
AGCTGAGAGCTCCAGCCTGGCCTCCAGATAACTACACACCCAGCTTCCATAGTCAGTATCAATTCTGGAAGAATTTCCAGACATTAAAGA
TAGTCATCTTGGGGCTGGTCCTGAGCA

Sequence color annotation: CCR5 locus Microhomology L1-ORFeus insertions (-)

The alignment of example #1 read sequence with the L1-ORFeus vector showed this insertion is in the intron region.

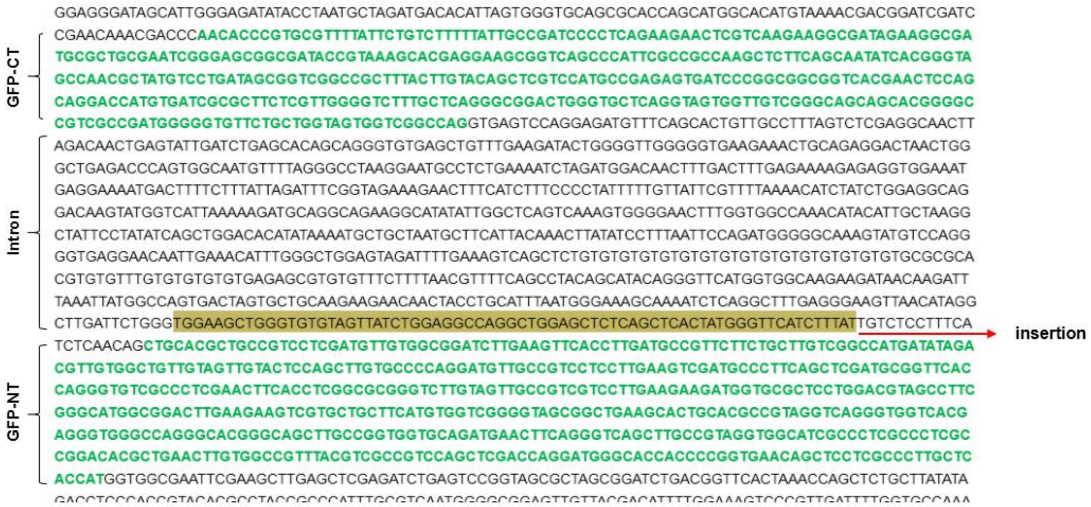

Supplementary Sequence 11. Example sequences of PolyA-seq from MYC CRISPR editing in HEK293T cells expressing L1-ORFeus.

Example #1

```
>M00701:791:000000000-CK823:1:1102:21019:22383
GCAATAAACAAGTTAACAACAAaaaaaaaaaaaaaaaaaaaaaaaaattctgaagtcacggcagtataccagtaaacgctctagga
ccttggtttttacaggtggcatggtatcgtgggacgccgggacacaggagaccttggaagtgggttgcggttaaccaccccgggcc
gttgaccacgcgtgccctatagtcttgcgacacagatcgaaagagcggtccagcaggaattccgagacagacatcgt
```

Alignment of reads sequence (top) with genomic sequence (bottom)

L1-ORFeus insertion at chr1:2817810 strand (+) Consensus motif

```
0000001 GCAATAAACAAGTTAACAACAAaaaaaaaaaaaaaaaaaaaaaaaaattc 0000050reads sequence
>>>>>>>                                           |||||||| >>>>>>>
2817768 gatcctggcctcatgatataaaggattggattttgatttcttaaaaattc 2817817Genomic sequence

0000051 tgaagtcacggcagtataccagtaaacgctctaggaccttggtttttaca 0000100
>>>>>>> |||||||||||||||||||||||||||||||||||||||||||| ||||| >>>>>>>
2817818 tgaagtcacggcagtataccagtaaacgctctaggaccttggttcttaca 2817867

0000101 ggtggcatggtatcgtgggacgccgggacacaggagaccttggaagtggg 0000150
>>>>>>> |||||||||||||||||||||||||||||||||||| ||||||||||||| >>>>>>>
2817868 ggtggcatggtatcgtgggacgccgggacacaggagtccttggaagtggg 2817917

0000151 ttgcgg 0000156
>>>>>>>  ||||| >>>>>>>
2817918 ctgcgg 2817923
```

Example #2

```
>M00701:791:000000000-CK823:1:1111:28823:11194
GCAATAAACAAGTTAACAACAAaaaaaaaaaaaaaaaaaaaacactcttcttttagtttttagttttttaaaaaaatattttcctt
tttcgttttggaagaaactaaaactttctacctaatagcattacaaacttgttaaaggggataaatgtgtattgatcttccttggt
ttttcttttcaggtttggggatatctcactctggtgtttatgggagggggagacaggtaaaggggagggagattctgc
```

L1-ORFeus insertion at chr5:151371709 strand (+)

```
000000001 GCAATAAACAAGTTAACAACAAaaaaaaaaaaaaaaaaaaaacactcttc 000000050
>>>>>>>>>                                      ||||||||||||| >>>>>>>>>
151371672 aggatgtgtaacaaggctagtgaataggattttttttaaaaacactcttc 151371721

000000051 ttttagtttttagttttttaaaaaaatattttcctttttcgttttggaag 000000100
>>>>>>>>> |||||||||||||||||||||||||||||||||||||||||||||||||| >>>>>>>>>
151371722 ttttagtttttagttttttaaaaaaatattttcctttttcgttttggaag 151371771

000000101 aaactaaaactttctacctaatagcattacaaacttgttaaaggggataa 000000150
>>>>>>>>> |||||||||||||||||||||||||||||||||| ||||||||||||||| >>>>>>>>>
151371772 aaactaaaactttctacctaatagcattacaaacatgttaaaggggataa 151371821

000000151 atgtgtattgatcttccttggtttttcttttcaggtttggggatatctca 000000200
>>>>>>>>> ||||||| |||||||||||||||||||||||||||||||||| ||||||| >>>>>>>>>
151371822 atgtgtactgatcttccttggtttttcttttcaggtttggggctatctca 151371871

000000201 ctctggtgtttatgggagggggagacaggtaaaggggagggagattctgc 000000250
>>>>>>>>> |||||||||||||||||| |||||||||| |||||||||||||||||||| >>>>>>>>>
151371872 ctctggtgtttatgggagcgggagacagggaaaggggagggagattctgc 151371921
```

Example #3

```
>M00701:791:000000000-CK823:1:1107:9554:10216
GCAATAAACAAGTTAACAACAAaaaaaaaaaaaaaaaaaaaaaagaatgtgtgaaattaaatctggtaacatagaaacatgaaata
ttaaaactgaactgcaatttttaagtacttaaaatattagatacagaataatatacacacatgtaatcaacaaatattaatatatt
taccagttagaattaataaatgatttacattggcttcaagtcttgttttatgtattaaaaaaaaaaagaagaaaaa
```

L1-ORFeus insertion at chr7:92573671 strand (-)

```
00000001 GCAATAAACAAGTTAACAACAAaaaaaaaaaaaaaaaaaaaaaagaatgt 00000050
<<<<<<<<                                  |||||||||||| |||| <<<<<<<<
92573704 acgattataaaataagtggttaccctcatatttaaaaaaaaaaagtatgt 92573655

00000051 gtgaaattaaatctggtaacatagaaacatgaaatattaaaactgaactg 00000100
<<<<<<<< |||||||||||||||||||||||||||||||||||||||||||||||||| <<<<<<<<
92573654 gtgaaattaaatctggtaacatagaaacatgaaatattaaaactgaactg 92573605

00000101 caatttttaagtacttaaaatattagatacagaataatatacacacatgt 00000150
<<<<<<<< ||||||||||||||||||||||||||||||||| |||||||||||||||| <<<<<<<<
92573604 caatttttaagtacttaaaatattagatacagagtaatatacacacatgt 92573555
```

```
00000151 aatcaacaaatattaatatatttaccagttagaattaataaatgatttac 00000200
<<<<<<<< |||||||||||||||||||||||||||||||||||||||||||||||||| <<<<<<<<
92573554 aatcaacaaatattaatatatttaccagttagaattaataaatgatttac 92573505

00000201 attggcttcaagtcttgttttatgtatt.aaaaaaaaaaagaagaaaaa 00000248
<<<<<<<< |||||||||||||||||||||||||||| |||||||||||||||||||| <<<<<<<<
92573504 attggcttcaagtcttgttttatgtattaaaaaaaaaaaaagaagaaaaa 92573456
```

Example #4

>M00701:791:000000000-CK823:1:1108:12186:18244

GCAATAAACAAGTTAACAACAAaaaaaaaaaaaaaaaaaaaaaattgttcaatagaggagctctacaatctaaaacatggttcagg
ggactagactaggctggcacagatagaagggtaggataaccagcccgggccgtcgaccacgcgtgccctatagtctagcgactcag
atcggaagagcggttcagcaggaatgccgagaccgaattcgaatgccgtcttctgcttgaaaaaaaaaaaaataaata

L1-ORFeus insertion at chr8:128450460 strand (+)

```
000000001 GCAATAAACAAGTTAACAACAAaaaaaaaaaaaaaaaaaaaaaattgttc 000000050
>>>>>>>>>                                       ||||||||||| >>>>>>>>>
128450421 tctgatgggtcgcgtataagtcaaattaagacgtatattaaaaattgttc 128450470

000000051 aatagaggagctctacaatctaaaacatggttcaggggactagactaggc 000000100
>>>>>>>>> |||||||||||||||||||||||||||||||||||||||||||||||||| >>>>>>>>>
128450471 aatagaggagctctacaatctaaaacatggttcaggggactagactaggc 128450520

000000101 tggcacagatagaagggtagg 000000121
>>>>>>>>> ||||||||||||||||||||| >>>>>>>>>
128450521 tggcacagatagaagggtagg 128450541
```

Example #5

>M00701:791:000000000-CK823:1:1104:25633:16154

GCAATAAACAAGTTAACAACAAaaaaaaaaaaaaaaaaaaagaaagataattcatgccccactgcttgttgtcctgcacgcaaat
acctaagttgagtcattaaatttttccacttctcaatggacatatgaggtacttctactccctcaccccatcaggcttcctagaag
atgctgcacgatgcctactattttaaccaaaggagcagaaaacacccagagaaacctatggtgtcagaaattttctct

L1-ORFeus insertion at chr8:134321397 strand (+)

```
000000001 GCAATAAACAAGTTAACAACAAaaaaaaaaaaaaaaaaaaagaaagata 000000050
>>>>>>>>>                                    |||||||||||||| >>>>>>>>>
134321361 gaaaagtcatataaaggccaactcagttgaaatttaaaaaagaaagata 134321410

000000051 attcatgccccactgcttgttgtcctgcacgcaaatacctaagttgagtc 000000100
>>>>>>>>> |||||||| ||||||||||||||||||||||||||||||||||||||||| >>>>>>>>>
134321411 attcatgctccactgcttgttgtcctgcacgcaaatacctaagttgagtc 134321460

000000101 attaaatttttccacttctcaatggacatatgaggtacttctactccctc 000000150
>>>>>>>>> |||||||||||||||||||||||||||||||||||||||||||||||||| >>>>>>>>>
134321461 attaaatttttccacttctcaatggacatatgaggtacttctactccctc 134321510

000000151 accccatcaggcttcctagaagatgctgcacgatgcctactattttaacc 000000200
>>>>>>>>> |||||||||||||||||||||||||||||||||||||||||||||||||| >>>>>>>>>
134321511 accccatcaggcttcctagaagatgctgcacgatgcctactattttaacc 134321560

000000201 aaaggagcagaaaacacccagagaaacctatggtgtcagaaattttctct 000000250
>>>>>>>>> |||||||||||||||||||||||||||||||||||||||||||||||||| >>>>>>>>>
134321561 aaaggagcagaaaacacccagagaaacctatggtgtcagaaattttctct 134321610
```

Example #6

>M00701:791:000000000-CK823:1:1113:11357:3801

GCAATAAACAAGTTAACAACAAaaaaaaaaaaaaaaaaaaaaattataattgtcagtgtgtgaccttagactatgaaaatgtatac
agttgtcaaagacccttttaatgcttcgaggacaaccagcccgggccgtcgaccacgcgtgccctatagtagcgtcgagatcggaa
gggcggttcagcaggaatgccgcgacagaacacgcatgccggcttttgtttgataaacaaaaaaaaatagatgtcact

L1-ORFeus insertion at chr9:27287255 strand (+)

```
00000001 GCAATAAACAAGTTAACAACAAaaaaaaaaaaaaaaaaaaaaattataat 00000050
>>>>>>>>                                     |||||||||||||| >>>>>>>>
27287218 gcaatgacaagcttccatgtctatattctctgatttaaaaaattataat 27287267

00000051 tgtcagtgtgtgaccttagactatgaaaatgtatacagttgtcaaagacc 00000100
>>>>>>>> ||||||||||||||||||||||||||||||||||||||||||||| |||  >>>>>>>>
27287268 tgtcagtgtgtgaccttagactatgaaaatgtatacagttgtcaatgact 27287317

00000101 cttttaatgcttcgagg 00000117
>>>>>>>> ||||||||||||||||| >>>>>>>>
27287318 cttttaatgcttcgagg 27287334
```

Example #7

>M00701:791:000000000-CK823:1:1108:15367:23104

GCAATAAACAAGTTAACAACAAaaaaaaaaaaaaaaaaaaaaaataaggggggggggggggagggacttaacaaaatcacaccaacg
tattttattaacaacatgcctaagtggtaagcttgctgccatttgtttcaaattcttgcagtttcttcttttgcagatattattta
ctccccacctgctgccagtcacgaccaaattgtataacactgtagactgctccaactctctgccttttaagctgaaat

L1-ORFeus insertion at chr9:32843666 strand (+)

```
00000001 GCAATAAACAAGTTAACAACAAaaaaaaaaaaaaaaaaaaaaaataaggg 00000050
>>>>>>>>                                   |||||||||||||||  >>>>>>>>
32843632 catgccttttagctctaagaaattgatttaagttaaaaaaaaaataaggt 32843681

00000051 ggggggggggagggacttaacaaaatcacaccaacgtattttattaacaa 00000100
>>>>>>>> || |||| ||||||||||||| |||||||||||||||||||||||||||| >>>>>>>>
32843682 ggtggggtggagggacttaaccaaatcacaccaacgtattttattaacaa 32843731

00000101 catgcctaagtggtaagcttgctgccatttgtttcaaattcttgcagttt 00000150
>>>>>>>> ||||||||||||||||||||||||||| ||| ||||||| | |||||||| >>>>>>>>
32843732 catgcctaagtggtaagcttgctgccacttgattcaaatgcctgcagttt 32843781

00000151 cttcttttgcagatattatttactccccacctgctgccagtcacgaccaa 00000200
>>>>>>>> ||||||||||||||||||||||||| |||||||||||||||||||  ||| >>>>>>>>
32843782 cttcttttgcagatattatttactctccacctgctgccagtcacgcacaa 32843831

00000201 attgtataacactgtagactgctccaactctctgccttttaagctgaaat 00000250
>>>>>>>> |||||||||||||||||||||||||||||||||||||||||||||||||| >>>>>>>>
32843832 attgtataacactgtagactgctccaactctctgccttttaagctgaaat 32843881
```

Example #8

>M00701:791:000000000-CK823:1:1102:5950:22540

GCAATAAACAAGTTAACAACAAaaaaaaaaaaaaaaaaaaaaaagtgaaactaaatcttttcattttttaaaattcattaattca
ttcagcaagtatttttgcatcatccaagcatatttgatcacgtgcttggcattcttttaggtcagtatttctcagcgttggataac
cagcccgggccgtcgaccacgcgtgccctatagtctagcgactcagatcggaaaggcggttcagcaggaatgccgat

L1-ORFeus insertion at chr11:8542738 strand (+)

```
0000001 GCAATAAACAAGTTAACAACAAaaaaaaaaaaaaaaaaaaaaaagtgaaa 0000050
&lt;&lt;&lt;&lt;&lt;&lt;&lt;                                    |||||||||||| || &lt;&lt;&lt;&lt;&lt;&lt;&lt;
8542773 gccactgcactccagcctgggtgacagagtgtcttaaaaaaaaagtgcaa 8542724

0000051 actaaatcttttcattttttaaaattcattaattcattcagcaagtattt 0000100
&lt;&lt;&lt;&lt;&lt;&lt;&lt; ||||||||||||||||| |||||||||||||||||||||||||||||||| &lt;&lt;&lt;&lt;&lt;&lt;&lt;
8542723 actaaatcttttcatttattaaaattcattaattcattcagcaagtattt 8542674

0000101 ttgcatcatccaagcatatttgatcacgtgcttggcattcttttaggtca 0000150
&lt;&lt;&lt;&lt;&lt;&lt;&lt; |||||||||||||||||||||||||||||||||||||||||||||||||| &lt;&lt;&lt;&lt;&lt;&lt;&lt;
8542673 ttgcatcatccaagcatatttgatcacgtgcttggcattcttttaggtca 8542624

0000151 gtatttctcagcgttgg 0000167
&lt;&lt;&lt;&lt;&lt;&lt;&lt; ||||||||||||||||| &lt;&lt;&lt;&lt;&lt;&lt;&lt;
8542623 gtatttctcagcgttgg 8542607
```

Supplementary Sequence 12. Example L1Hs sequences obtained by amplicon sequencing at the MYC/RAG1 CRISPR/Cas9 target site in HEK293T cells.

Trinucleotide in 3’UTR feature within indicated endogenous L1 family

Yong, active L1		Old L1		
L1Hs	L1RP			
ACG	ACA	AAG	GAG	CAG

Example #1 (at MYC CRISPR/Cas9 target site in HEK293T cells expressing L1-RTm)

> M00851:382:000000000-CF5PB:1:1104:23807:16329
CGATCATGATCGGCTGGTTCACTAAGTGCGTCTCCGAGATAGCAGGGGACTGTCCAAAGGGGGGTAGGAGGAGGGGGGAGGG
ATAGCATTGGGAGATATACCTAATGCTAGATGAC**ACA**TTAGTGGGTGCAGCGCACCAGCATGGCACATGTATACATATGTAACT
AACCTGCACAATGTGCACATGTACCCTAAAACTTAGAGTATAAT**AAAATAAA**GGGTGCTCCCTTTATTCCCCCACCAAGACCAC
CCAGCCGCTTTAGGGGATAGCTCTGCAAGGGAGAG

Sequence color annotation: MYC locus L1Hs sequences (+) Trinucleotide in 3’UTR PolyA tail Microhomology

Example #2 (at RAG1 CRISPR/Cas9 target site in HEK293T cells expressing L1-ORFeus)

> M00701:748:000000000-CFM62:1:2104:18741:6169
ACGCGCTGCGTAAGATACATCAGTGGGATATTGATATTGGTCTTAATATGACTTGTTTTCATTGTTCTCAGGTACCTCAGCCAGCA
TGGCAGCCTCTTTCCCACCCACTTTTTTTTTTATTATACTCTAAGTTTTAGGGTACATGTGCACATTGTGCAGGTTAGTTACATATG
TATACATGTGCCATGCTGGTGCACTGCACCCACTAA**TGT**GTCATCTAGCATTAGGTATATATGGGGCTCAGTGCTGCCCCAGATG
AAATTCAGCACCCACATATTAAATTTTCAGAATGGAAATTTAAGCTGTTCCGGGTGAGATCCT

Reverse complementary sequence:

AGGATCTCACCCGGAACAGCTTAAATTTCCATTCTGAAAATTTAATATGTGGGTGCTGAATTTCATCTGGGGCAGCACTGAGCCC
CATATATACCTAATGCTAGATGAC**ACA**TTAGTGGGTGCAGTGCACCAGCATGGCACATGTATACATATGTAACTAACCTGCACA
ATGTGCACATGTACCCTAAAACTTAGAGTATAAT**AAAAAAAAAA**GTGGGTGGGAAAGAGGCTGCCATGCTGGCTGAGGTACCT
GAGAACAATGAAAACAAGTCATATTAAGACCAATATCAATATCCCACTGATGTATCTTACGCAGCGCGT

Sequence color annotation: RAG1 locus L1Hs sequences (-) Trinucleotide in 3’UTR PolyA tail

Supplementary Sequence 13. DNA sequences of pCEP4 L1RP, pCEP4 L1RP-ENm (H230A) (L1RP-ENm), and pCEP4 L1RP-RTm (D702Y) (L1RP-RTm) plasmids used in this study.

13.1 pCEP4 L1RP (1-17897bp) can be found in https://www.addgene.org/131392/

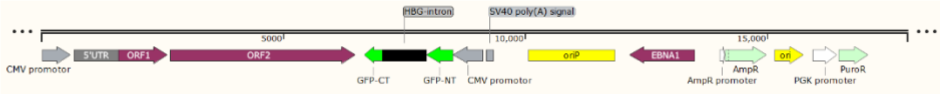

Features	Regions (bp)
CMV promoter	30-605
L1 5'UTR	689-1594
ORF1 (L1RP)	1595-2611
ORF2 (L1RP)	2675-6502
**GFP-CT**	**6681-7065**
HBG-intron	7066-7965
**GFP-NT**	**7966-8499**

GTTGACATTGATTATTGACTAGTTATTAATAGTAATCAATTACGGGGTCATTAGTTCATAGCCCATATATGGAGTTCCGCGTTACGTAACTTACGGT
AAATGGCCCGCCTGGCTGACCGCCCAACGACCCCCGCCCATTGACGTCAATAATGACGTATGTTCCCATAGTAACGCCAATAGGGACTTTCC
ATTGACGTCAATGGGTGGAGTATTTACGGTAAACTGCCCACTTGGCAGTACATCAAGTGTATCATATGCCAAGTCCGCCCCCTATTGACGTCAA
TGACGGTAAATGGCCCGCCTGGCATTATGCCCAGTACATGACCTTACGGGACTTTCCTACTTGGCAGTACATCTACGTATTAGTCATCGCTATT
ACCATGGTGATGCGGTTTTGACAGTACACCAATGGGCGTGGATAGCGGTTTGACTCACGGGGATTTCCAAGTCTCCACCCCATTGACGTCAA
TGGGAGTTTGTTTTGGCACCAAAATCAACGGGACTTTCCAAAATGTCGTAATAACCCCGCCCCGTTGACGCAAATGGGCGGTAGGCGTGTAC
GGTGGGAGGTCTATATAAGCAGAGCTCGTTTAGTGAACCGTCAGATCTCTAGAAGCTGGGTACCAGCTGCTAGCAAGCTTGCTAGCGGCCGC
TCTAGCCCTGGAATGTGTATTAAGACTGTAAGGTGGGGGGGAGGAGCCAAGATGGCCGAATAGGAACAGCTCCGGTCTACAGCTCCCAGCG
TGAGCGACGCAGAAGACGGTGATTTCTGCATTTCCATCTGAGGTACCGGGTTCATCTCACTAGGGAGTGCCAGACAGTGGGCGCAGGCCAG
TGTGTGTGCGCACCGTGCGCGAGCCGAAGCAGGGCGAGGCATTGCCTCACCTGGGAAGCGCAAGGGGTCAGGGAGTTCCCTTTCCGAGT
CAAAGAAAGGGGTGACGGACGCACCTGGAAAATCGGGTCACTCCCACCCGAATATTGCGCTTTTCAGACCGGCTTAAGAAACGGCGCACCA
CGAGACTATATCCCGCACCTGGCTCGGAGGGTCCTACGCCCACGGAATCTCGCTGATTGCTAGCACAGCAGTCTGAGATCAAACTGCAAGG
CGGCAACGAGGCTGGGGGAGGGGCGCCCGCCATTGCCCAGGCTTGCTTAGGTAAACAAAGCAGCAGGGAAGCTCGAACTGGGTGGAGCC
CACCACAGCTCAAGGAGGCCTGCCTGCCTCTGTAGGCTCCACCTCTGGGGGCAGGGCACAGACAAACAAAAAGACAGCAGTAACCTCTGC
AGACTTAAGTGTCCCTGTCTGACAGCTTTGAAGAGAGCAGTGGTTCTCCCAGCACGCAGCTGGAGATCTGAGAACGGGCAGACTGCCTCCT
CAAGTGGGTCCCTGACCCCTGACCCCCGAGCAGCCTAACTGGGAGGCACCCCCCAGCAGGGGCACACTGACACCTCACACGGCAGGGTA
TTCCAACAGACCTGCAGCTGAGGGTCCTGTCTGTTAGAAGGAAAACTAACAACCAGAAAGGACATCTACACCGAAAACCCATCTGTACATCAC
CATCATCAAAGACCAAAAGTAGATAAAACCACAAAGATGGGGAAAAAACAGAACAGAAAAACTGGAAACTCTAAAACGCAGAGCGCCTCTCCT
CCTCCAAAGGAACGCAGTTCCTCACCAGCAACAGAACAAAGCTGGATGGAGAATGATTTTGATGAGCTGAGAGAAGAAGGCTTCAGACGATC
AAATTACTCTGAGCTACGGGAGGACATTCAAACCAAAGGCAAAGAAGTTGAAAACTTTGAAAAAAATTTAGAAGAATGTATAACTAGAATAACCA
ATACAGAGAAGTGCTTAAAGGAGCTGATGGAGCTGAAAACCAAGGCTCGAGAACTACGTGAAGAATGCAGAAGCCTCAGGAGCCGATGCGA
TCAACTGGAAGAAAGGGTATCAGCAATGGAAGATGAAATGAATGAAATGAAGCGAGAAGGGAAGTTTAGAGAAAAAAGAATAAAAAGAAATGA
GCAAAGCCTCCAAGAAATATGGGACTATGTGAAAAGACCAAATCTACGTCTGATTGGTGTACCTGAAAGTGATGTGGAGAATGGAACCAAGTT
GGAAAACACTCTGCAGGATATTATCCAGGAGAACTTCCCCAATCTAGCAAGGCAGGCCAACGTTCAGATTCAGGAAATACAGAGAACGCCAC
AAAGATACTCCTCGAGAAGAGCAACTCCAAGACACATAATTGTCAGATTCACCAAAGTTGAAATGAAGGAAAAAATGTTAAGGGCAGCCAGAG
AGAAAGGTCGGGTTACCCTCAAAGGAAAGCCCATCAGACTAACAGCGGATCTCTCGGCAGAAACCCTACAAGCCAGAAGAGAGTGGGGGC
CAATATTCAACATTCTTAAAGAAAAGAATTTTCAACCCAGAATTTCATATCCAGCCAAACTAAGCTTCATAAGTGAAGGAGAAATAAAATACTTTAT
AGACAAGCAAATGTTGAGAGATTTTGTCACCACCAGGCCTGCCCTAAAAGAGCTCCTGAAGGAAGCGCTAAACATGGAAAGGAACAACCGGT
ACCAGCCGCTGCAAAATCATGCCAAAATGTAAAGACCATCAAGACTAGGAAGAAACTGCACCAACTAATGAGCAAAATCACCAGCTAACATCA
TAATGACAGGATCAACTTCACACATAACAATATTAACTTTAAATATAAATGGACTAAATTCTGCAATTAAAAGACACAGACTGGCAAGTTGGATAA
AGAGTCAAGACCCATCAGTGTGCTGTATTCAGGAAACCCATCTCACGTGCAGAGACACACATAGGCTCAAAATAAAAGGATGGAGGAAGATCT
ACCAAGCCAATGGAAAACAAAAAAAGGCAGGGGTTGCAATCCTAGTCTCTGATAAAACAGACTTTAAACCAACAAAGATCAAAAGAGACAAAG
AAGGCCATTACATAATGGTAAAGGGATCAATTCAACAAGAGGAGCTAACTATCCTAAATATTTATGCACCCAATACAGGAGCACCCAGATTCATA
AAGCAAGTCCTCAGTGACCTACAAAGAGACTTAGACTCCCACACATTAATAATGGGAGACTTTAACACCCCACTGTCAACATTAGACAGATCAA
CGAGACAGAAAGTCAACAAGGATACCCAGGAATTGAACTCAGCTCTGCACCAAGCAGACCTAATAGACATCTACAGAACTCTCCACCCCAAAT
CAACAGAATATACGTTTTTTTCAGCACCACACCACACCTATTCCAAAATTGACCACATAGTTGGAAGCAAAGCTCTCCTCAGCAAATGTAAAAG
AACAGAAATTATAACAAACTATCTCTCAGACCACAGTGCAATCAAACTAGAGCTCAGGATTAAGAATCTCACTCAAAGCCGCTCAACTACATGG
AAACTGAACAACCTGCTCCTGAATGACTACTGGGTACATAACGAAATGAAGGCAGAAATAAAGATGTTCTTTGAAACCAACGAGAACAAAGAC
ACCACATACCAGAATCTCTGGGACGCATTCAAAGCAGTATGTAGAGGGAAATTTATAGCACTAAATGCCTACAAGAGAAAGCAGGAAAGATCC
AAAATTGACACCCTAACATCACAATTAAAAGAACTAGAAAAGCAAGAGCAAACACATTCAAAAGCTAGCAGAAGGCAAGAAATAACTAAAATCA
GAGCAGAACTGAAGGAAATAGAGACACAAAAAACCCTTCAAAAAATCAATGAATCCAGGAGCTGGTTTTTTGAAAGGATCAACAAAATTGATA
GACCGCTAGCAAGACTAATAAAGAAAAAAAGAGAGAAGAATCAAATAGACACAATAAAAAATGATAAAGGGGATATCACCACCGATCCCACAGA
AATACAAACTACCATCAGAGAATACTACAAACACCTCTACGCAAATAAACTAGAAAATCTAGAAGAAATGGATACATTCCTCGACACATACACTCT
CCCAAGACTAAACCAGGAAGAAGTTGAATCTCTGAATCGACCAATAACAGGCTCTGAAATTGTGGCAATAATCAATAGTTTACCAACCAAAAAG
AGTCCAGGACCAGATGGATTCACAGCCGAATTCTACCAGAGGTACAAGGAGGAACTGGTACCATTCCTTCTGAAACTATTCCAATCAATAGAA
AAAGAGGGAATCCTCCCTAACTCATTTTATGAGGCCAGCATCATTCTGATACCAAAGCCGGGCAGAGACACAACCAAAAAAGAGAATTTTAGA
CCAATATCCTTGATGAACATTGATGCAAAAATCCTCAATAAAATACTGGCAAACCGAATCCAGCAGCACATCAAAAAGCTTATCCACCATGATCA
AGTGGGCTTCATCCCTGGGATGCAAGGCTGGTTCAATATACGCAAATCAATAAATGTAATCCAGCATATAAACAGAGCCAAAGACAAAAACCAC

ATGATTATCTCAATAGATGCAGAAAAAGCCTTTGACAAAATTCAACAACCCTTCATGCTAAAAACTCTCAATAAATTAGGTATTGATGGGACGTAT
TTCAAAATAATAAGAGCTATCTATGACAAACCCACAGCCAATATCATACTGAATGGGCAAAAACTGGAAGCATTCCCTTTGAAAACCGGCACAA
GACAGGGATGCCCTCTCTCACCGCTCCTATTCAACATAGTGTTGGAAGTTCTGGCCAGGGCAATCAGGCAGGAGAAGGAAATAAAGGGTATT
CAATTAGGAAAAGAGGAAGTCAAATTGTCCCTGTTTGCAGACGACATGATTGTTTATCTAGAAAACCCCATCGTCTCAGCCCAAAATCTCCTTA
AGCTGATAAGCAACTTCAGCAAAGTCTCAGGATACAAAATCAATGTACAAAAATCACAAGCATTCTTATACACCAACAACAGACAAACAGAGAG
CCAAATCATGGGTGAACTCCCATTCACAATTGCTTCAAAGAGAATAAAATACCTAGGAATCCAACTTACAAGGGATGTGAAGGACCTCTTCAAG
GAGAACTACAAACCACTGCTCAAGGAAATAAAAGAGGAGACAAACAAATGGAAGAACATTCCATGCTCATGGGTAGGAAGAATCAATATCGTG
AAAATGGCCATACTGCCCAAGGTAATTTACAGATTCAATGCCATCCCCATCAAGCTACCAATGACTTTCTTCACAGAATTGGAAAAAACTACTTT
AAAGTTCATATGGAACCAAAAAAGAGCCCGCATTGCCAAGTCAATCCTAAGCCAAAAGAACAAAGCTGGAGGCATCACACTACCTGACTTCAA
ACTATACTACAAGGCTACAGTAACCAAAACAGCATGGTACTGGTACCAAAACAGAGATATAGATCAATGGAACAGAACAGAGCCCTCAGAAATA
ATGCCGCATATCTACAACTATCTGATCTTTGACAAACCTGAGAAAAACAAGCAATGGGGAAAGGATTCCCTATTTAATAAATGGTGCTGGGAAA
ACTGGCTAGCCATATGTAGAAAGCTGAAACTGGATCCCTTCCTTACACCTTATACAAAAATCAATTCAAGATGGATTAAAGATTTAAACGTTAAAC
CTAAAACCATAAAAACCCTAGAAGAAAACCTAGGCATTACCATTCAGGACATAGGCGTGGGCAAGGACTTCATGTCCAAAACACCAAAAGCAA
TGGCAACAAAAGACAAAATTGACAAATGGGATCTAATTAAACTAAAGAGCTTCTGCACAGCAAAAGAAACTACCATCAGAGTGAACAGGCAAC
CTACAACATGGGAGAAAATTTTTGCAACCTACTCATCTGACAAAGGGCTAATATCCAGAATCTACAATGAACTCAAACAAATTTACAAGAAAAAA
ACAAACAACCCCATCAAAAAGTGGGCGAAGGACATGAACAGACACTTCTCAAAAGAAGACATTTATGCAGCCAAAAAACACATGAAGAAATGC
TCATCATCACTGGCCATCAGAGAAATGCAAATCAAAACCACTATGAGATATCATCTCACACCAGTTAGAATGGCAATCATTAAAAAGTCAGGAAA
CAACAGGTGCTGGAGAGGATGCGGAGAAATAGGAACACTTTTACACTGTTGGTGGGACTGTAAACTAGTTCAACCATTGTGGAAGTCAGTGT
GGCGATTCCTCAGGGATCTAGAACTAGAAATACCATTTGACCCAGCCATCCCATTACTGGGTATATACCCAAATGAGTATAAATCATGCTGCTAT
AAAGACACATGCACACGTATGTTTATTGCGGCACTATTCACAATAGCAAAGACTTGGAACCAACCCAAATGTCCAACAATGATAGACTGGATTA
AGAAAATGTGGCACATATACACCATGGAATACTATGCAGCCATAAAAAATGATGAGTTCATATCCTTTGTAGGGACATGGATGAAATTGGAAACC
ATCATTCTCAGTAAACTATCGCAAGAACAAAAAACCAAACACCGCATATTCTCACTCATAGGTGGGAATTGAACAATGAGATCACATGGACACA
GGAAGGGGAATATCACACTCTGGGGACTGTGGTGGGGTCGGGGGAGGGGGGAGGGATAGCATTGGGAGATATACCTAATGCTAGATGACAC
ATTAGTGGGTGCAGCGCACCAGCATGGCACATGTAAAACGACGGATCGATCCGAACAAACGACCC**AACACCCGTGCGTTTTATTCTGTCTTT**
TTATTGCCGATCCCCTCAGAAGAACTCGTCAAGAAGGCGATAGAAGGCGATGCGCTGCGAATCGGGAGCGGCGATACCGTAAAGCACGA
GGAAGCGGTCAGCCCATTCGCCGCCAAGCTCTTCAGCAATATCACGGGTAGCCAACGCTATGTCCTGATAGCGGTCGGCCGCTTTACTTG
TACAGCTCGTCCATGCCGAGAGTGATCCCGGCGGCGGTCACGAACTCCAGCAGGACCATGTGATCGCGCTTCTCGTTGGGGTCTTTGCTC
AGGGCGGACTGGGTGCTCAGGTAGTGGTTGTCGGGCAGCAGCACGGGGCCGTCGCCGATGGGGGTGTTCTGCTGGTAGTGGTCGGCCA
GGTGAGTCCAGGAGATGTTTCAGCACTGTTGCCTTTAGTCTCGAGGCAACTTAGACAACTGAGTATTGATCTGAGCACAGCAGGGTGTGAGC
TGTTTGAAGATACTGGGGTTGGGGGTGAAGAAACTGCAGAGGACTAACTGGGCTGAGACCCAGTGGCAATGTTTTAGGGCCTAAGGAATGC
CTCTGAAAATCTAGATGGACAACTTTGACTTTGAGAAAAGAGAGGTGGAAATGAGGAAAATGACTTTTCTTTATTAGATTTCGGTAGAAAGAAC
TTTCATCTTTCCCCTATTTTTGTTATTCGTTTTAAAACATCTATCTGGAGGCAGGACAAGTATGGTCATTAAAAAGATGCAGGCAGAAGGCATATA
TTGGCTCAGTCAAAGTGGGGAACTTTGGTGGCCAAACATACATTGCTAAGGCTATTCCTATATCAGCTGGACACATATAAAATGCTGCTAATGC
TTCATTACAAACTTATATCCTTTAATTCCAGATGGGGGCAAAGTATGTCCAGGGGTGAGGAACAATTGAAACATTTGGGCTGGAGTAGATTTTG
AAAGTCAGCTCTGTGTGTGTGTGTGTGTGTGTGTGTGTGTGTGTGTGCGCGCACGTGTGTTTGTGTGTGTGTGAGAGCGTGTGTTTCTTTTAAC
GTTTTCAGCCTACAGCATACAGGGTTCATGGTGGCAAGAAGATAACAAGATTTAAATTATGGCCAGTGACTAGTGCTGCAAGAAGAACAACTAC
CTGCATTTAATGGGAAAGCAAAATCTCAGGCTTTGAGGGAAGTTAACATAGGCTTGATTCTGGGTGGAAGCTGGGTGTGTAGTTATCTGGAGG
CCAGGCTGGAGCTCTCAGCTCACTATGGGTTCATCTTTATTGTCTCCTTTCATCTCAACAG**CTGCACGCTGCCGTCCTCGATGTTGTGGCGG**
ATCTTGAAGTTCACCTTGATGCCGTTCTTCTGCTTGTCGGCCATGATATAGACGTTGTGGCTGTTGTAGTTGTACTCCAGCTTGTGCCCCAG
GATGTTGCCGTCCTCCTTGAAGTCGATGCCCTTCAGCTCGATGCGGTTCACCAGGGTGTCGCCCTCGAACTTCACCTCGGCGCGGGTCTT
GTAGTTGCCGTCGTCCTTGAAGAAGATGGTGCGCTCCTGGACGTAGCCTTCGGGCATGGCGGACTTGAAGAAGTCGTGCTGCTTCATGTG
GTCGGGGTAGCGGCTGAAGCACTGCACGCCGTAGGTCAGGGTGGTCACGAGGGTGGGCCAGGGCACGGGCAGCTTGCCGGTGGTGCA
GATGAACTTCAGGGTCAGCTTGCCGTAGGTGGCATCGCCCTCGCCCTCGCCGGACACGCTGAACTTGTGGCCGTTTACGTCGCCGTCCA
GCTCGACCAGGATGGGCACCACCCCGGTGAACAGCTCCTCGCCCTTGCTCACCATGGTGGCGAATTCGAAGCTTGAGCTCGAGATCTGAG
TCCGGTAGCGCTAGCGGATCTGACGGTTCACTAAACCAGCTCTGCTTATATAGACCTCCCACCGTACACGCCTACCGCCCATTTGCGTCAATG
GGGCGGAGTTGTTACGACATTTTGGAAAGTCCCGTTGATTTTGGTGCCAAAACAAACTCCCATTGACGTCAATGGGGTGGAGACTTGGAAAT
CCCCGTGAGTCAAACCGCTATCCACGCCCATTGATGTACTGCCAAAACCGCATCACCATGGTAATAGCGATGACTAATACGTAGATGTACTGC
CAAGTAGGAAAGTCCCATAAGGTCATGTACTGGGCATAATGCCAGGCGGGCCATTTACCGTCATTGACGTCAATAGGGGGCGTACTTGGCATA
TGATACACTTGATGTACTGCCAAGTGGGCAGTTTACCGTAAATACTCCACCCATTGACGTCAATGGAAAGTCCCTATTGGCGTTACTATGGGAA
CATACGTCATTATTGACGTCAATGGGCGGGGGTCGTTGGGCGGTCAGCCAGGCGGGCCATTTACCGTAAGTTATGTAACGCGGAACTCCATA
TATGGGCTATGAACTAATGACCCCGTAATTGATTACTATTAGCCCGGGCAATGTGCACATGTACCCTAAAACTTAAAGTATAATAAAGACGTCAG
GGTTCGAAATCGATAAGCTTGGATCCAGACATGATAAGATACATTGATGAGTTTGGACAAACCACAACTAGAATGCAGTGAAAAAAATGCTTTAT
TTGTGAAATTTGTGATGCTATTGCTTTATTTGTAACCATTATAAGCTGCAATAAACAAGTTAACAACAACAATTGCATTCATTTTATGTTTCAGGTT
CAGGGGGAGGTGTGGGAGGTTTTTTAAAGCAAGTAAAACCTCTACAAATGTGGTATGGCTGATTATGATCCGGCTGCCTCGCGCGTTTCGGT
GATGACGGTGAAAACCTCTGACACATGCAGCTCCCGGAGACGGTCACAGCTTGTCTGTAAGCGGATGCCGGGAGCAGACAAGCCCGTCAG
GGCGCGTCAGCGGGTGTTGGCGGGTGTCGGGGCGCAGCCATGAGGTCGATCGACTCTAGAGGATCGATCCCCGCCCCGGACGAACTAAA
CCTGACTACGACATCTCTGCCCCTTCTTCGCGGGGCAGTGCATGTAATCCCTTCAGTTGGTTGGTACAACTTGCCAACTGGGCCCTGTTCCA
CATGTGACACGGGGGGGGACCAAACACAAAGGGGTTCTCTGACTGTAGTTGACATCCTTATAAATGGATGTGCACATTTGCCAACACTGAGT
GGCTTTCATCCTGGAGCAGACTTTGCAGTCTGTGGACTGCAACACAACATTGCCTTTATGTGTAACTCTTGGCTGAAGCTCTTACACCAATGC
TGGGGGACATGTACCTCCCAGGGGCCCAGGAAGACTACGGGAGGCTACACCAACGTCAATCAGAGGGGCCTGTGTAGCTACCGATAAGCG
GACCCTCAAGAGGGCATTAGCAATAGTGTTTATAAGGCCCCCTTGTTAACCCTAAACGGGTAGCATATGCTTCCCGGGTAGTAGTATATACTATC
CAGACTAACCCTAATTCAATAGCATATGTTACCCAACGGGAAGCATATGCTATCGAATTAGGGTTAGTAAAAGGGTCCTAAGGAACAGCGATATC
TCCCACCCCATGAGCTGTCACGGTTTTATTTACATGGGGTCAGGATTCCACGAGGGTAGTGAACCATTTTAGTCACAAGGGCAGTGGCTGAA
GATCAAGGAGCGGGCAGTGAACTCTCCTGAATCTTCGCCTGCTTCTTCATTCTCCTTCGTTTAGCTAATAGAATAACTGCTGAGTTGTGAACA
GTAAGGTGTATGTGAGGTGCTCGAAAACAAGGTTTCAGGTGACGCCCCCAGAATAAAATTTGGACGGGGGGTTCAGTGGTGGCATTGTGCTA
TGACACCAATATAACCCTCACAAACCCCTTGGGCAATAAATACTAGTGTAGGAATGAAACATTCTGAATATCTTTAACAATAGAAATCCATGGGG
TGGGGACAAGCCGTAAAGACTGGATGTCCATCTCACACGAATTTATGGCTATGGGCAACACATAATCCTAGTGCAATATGATACTGGGGTTATT
AAGATGTGTCCCAGGCAGGGACCAAGACAGGTGAACCATGTTGTTACACTCTATTTGTAACAAGGGGAAAGAGAGTGGACGCCGACAGCAG
CGGACTCCACTGGTTGTCTCTAACACCCCCGAAAATTAAACGGGGCTCCACGCCAATGGGGCCCATAAACAAAGACAAGTGGCCACTCTTTT

TTTTGAAATTGTGGAGTGGGGGCACGCGTCAGCCCCCACACGCCGCCCTGCGGTTTTGGACTGTAAAATAAGGGTGTAATAACTTGGCTGAT
TGTAACCCCGCTAACCACTGCGGTCAAACCACTTGCCCACAAAACCACTAATGGCACCCCGGGGAATACCTGCATAAGTAGGTGGGCGGGC
CAAGATAGGGGCGCGATTGCTGCGATCTGGAGGACAAATTACACACACTTGCGCCTGAGCGCCAAGCACAGGGTTGTTGGTCCTCATATTCA
CGAGGTCGCTGAGAGCACGGTGGGCTAATGTTGCCATGGGTAGCATATACTACCCAAATATCTGGATAGCATATGCTATCCTAATCTATATCTGG
GTAGCATAGGCTATCCTAATCTATATCTGGGTAGCATATGCTATCCTAATCTATATCTGGGTAGTATATGCTATCCTAATTTATATCTGGGTAGCATA
GGCTATCCTAATCTATATCTGGGTAGCATATGCTATCCTAATCTATATCTGGGTAGTATATGCTATCCTAATCTGTATCCGGGTAGCATATGCTATCC
TAATAGAGATTAGGGTAGTATATGCTATCCTAATTTATATCTGGGTAGCATATACTACCCAAATATCTGGATAGCATATGCTATCCTAATCTATATCTG
GGTAGCATATGCTATCCTAATCTATATCTGGGTAGCATAGGCTATCCTAATCTATATCTGGGTAGCATATGCTATCCTAATCTATATCTGGGTAGTAT
ATGCTATCCTAATTTATATCTGGGTAGCATAGGCTATCCTAATCTATATCTGGGTAGCATATGCTATCCTAATCTATATCTGGGTAGTATATGCTATC
CTAATCTGTATCCGGGTAGCATATGCTATCCTCATGCATATACAGTCAGCATATGATACCCAGTAGTAGAGTGGGAGTGCTATCCTTTGCATATGC
CGCCACCTCCCAAGGGGGCGTGAATTTTCGCTGCTTGTCCTTTTCCTGCTGGTTGCTCCCATTCTTAGGTGAATTTAAGGAGGCCAGGCTAA
AGCCGTCGCATGTCTGATTGCTCACCAGGTAAATGTCGCTAATGTTTTCCAACGCGAGAAGGTGTTGAGCGCGGAGCTGAGTGACGTGACAA
CATGGGTATGCCCAATTGCCCCATGTTGGGAGGACGAAAATGGTGACAAGACAGATGGCCAGAAATACACCAACAGCACGCATGATGTCTAC
TGGGGATTTATTCTTTAGTGCGGGGGAATACACGGCTTTTAATACGATTGAGGGCGTCTCCTAACAAGTTACATCACTCCTGCCCTTCCTCACC
CTCATCTCCATCACCTCCTTCATCTCCGTCATCTCCGTCATCACCCTCCGCGGCAGCCCCTTCCACCATAGGTGGAAACCAGGGAGGCAAAT
CTACTCCATCGTCAAAGCTGCACACAGTCACCCTGATATTGCAGGTAGGAGCGGGCTTTGTCATAACAAGGTCCTTAATCGCATCCTTCAAAA
CCTCAGCAAATATATGAGTTTGTAAAAAGACCATGAAATAACAGACAATGGACTCCCTTAGCGGGCCAGGTTGTGGGCCGGGTCCAGGGGCC
ATTCCAAAGGGGAGACGACTCAATGGTGTAAGACGACATTGTGGAATAGCAAGGGCAGTTCCTCGCCTTAGGTTGTAAAGGGAGGTCTTACT
ACCTCCATATACGAACACACCGGCGACCCAAGTTCCTTCGTCGGTAGTCCTTTCTACGTGACTCCTAGCCAGGAGAGCTCTTAAACCTTCTGC
AATGTTCTCAAATTTCGGGTTGGAACCTCCTTGACCACGATGCTTTCCAAACCACCCTCCTTTTTTGCGCCTGCCTCCATCACCCTGACCCCG
GGGTCCAGTGCTTGGGCCTTCTCCTGGGTCATCTGCGGGGCCCTGCTCTATCGCTCCCGGGGGCACGTCAGGCTCACCATCTGGGCCACC
TTCTTGGTGGTATTCAAAATAATCGGCTTCCCCTACAGGGTGGAAAAATGGCCTTCTACCTGGAGGGGGCCTGCGCGGTGGAGACCCGGAT
GATGATGACTGACTACTGGGACTCCTGGGCCTCTTTTCTCCACGTCCACGACCTCTCCCCCTGGCTCTTTCACGACTTCCCCCCCTGGCTCT
TTCACGTCCTCTACCCCGGCGGCCTCCACTACCTCCTCGACCCCGGCCTCCACTACCTCCTCGACCCCGGCCTCCACTGCCTCCTCGACCC
CGGCCTCCACCTCCTGCTCCTGCCCCTCCTGCTCCTGCCCCTCCTCCTGCTCCTGCCCCTCCTGCCCCTCCTCCTGCTCCTGCCCCTCCTC
CTGCTCCTGCCCCTCCTGCTCCTGCCCCTCCCGCTCCTGCTCCTGCTCCTGTTCCACCGTGGGTCCCTTTGCAGCCAATGCAACTTGGACG
TTTTTGGGGTCTCCGGACACCATCTCTATGTCTTGGCCCTGATCCTGAGCCGCCCGGGGCTCCTGGTCTTCCGCCTCCTCGTCCTCGTCCT
CTTCCCCGTCCTCGTCCATGGTTATCACCCCCTCTTCTTTGAGGTCCACTGCCGCCGGAGCCTTCTGGTCCAGATGTGTCTCCCTTCTCTCC
TAGGCCATTTCCAGGTCCTGTACCTGGCCCCTCGTCAGACATGATTCACACTAAAAGAGATCAATAGACATCTTTATTAGACGACGCTCAGTGA
ATACAGGGAGTGCAGACTCCTGCCCCCTCCAACAGCCCCCCCACCCTCATCCCCTTCATGGTCGCTGTCAGACAGATCCAGGTCTGAAAATT
CCCCATCCTCCGAACCATCCTCGTCCTCATCACCAATTACTCGCAGCCCGGAAAACTCCCGCTGAACATCCTCAAGATTTGCGTCCTGAGCC
TCAAGCCAGGCCTCAAATTCCTCGTCCCCCTTTTTGCTGGACGGTAGGGATGGGGATTCTCGGGACCCCTCCTCTTCCTCTTCAAGGTCACC
AGACAGAGATGCTACTGGGGCAACGGAAGAAAAGCTGGGTGCGGCCTGTGAGGATCAGCTTATCGATGATAAGCTGTCAAACATGAGAATTC
TTGAAGACGAAAGGGCCTCGTGATACGCCTATTTTTATAGGTTAATGTCATGATAATAATGGTTTCTTAGACGTCAGGTGGCACTTTTCGGGGA
AATGTGCGCGGAACCCCTATTTGTTTATTTTTCTAAATACATTCAAATATGTATCCGCTCATGAGACAATAACCCTGATAAATGCTTCAATAATATT
GAAAAAGGAAGAGTATGAGTATTCAACATTTCCGTGTCGCCCTTATTCCCTTTTTTGCGGCATTTTGCCTTCCTGTTTTTGCTCACCCAGAAAC
GCTGGTGAAAGTAAAAGATGCTGAAGATCAGTTGGGTGCACGAGTGGGTTACATCGAACTGGATCTCAACAGCGGTAAGATCCTTGAGAGTT
TTCGCCCCGAAGAACGTTTTCCAATGATGAGCACTTTTAAAGTTCTGCTATGTGGCGCGGTATTATCCCGTGTTGACGCCGGGCAAGAGCAAC
TCGGTCGCCGCATACACTATTCTCAGAATGACTTGGTTGAGTACTCACCAGTCACAGAAAAGCATCTTACGGATGGCATGACAGTAAGAGAAT
TATGCAGTGCTGCCATAACCATGAGTGATAACACTGCGGCCAACTTACTTCTGACAACGATCGGAGGACCGAAGGAGCTAACCGCTTTTTTGC
ACAACATGGGGGATCATGTAACTCGCCTTGATCGTTGGGAACCGGAGCTGAATGAAGCCATACCAAACGACGAGCGTGACACCACGATGCCT
GCAGCAATGGCAACAACGTTGCGCAAACTATTAACTGGCGAACTACTTACTCTAGCTTCCCGGCAACAATTAATAGACTGGATGGAGGCGGAT
AAAGTTGCAGGACCACTTCTGCGCTCGGCCCTTCCGGCTGGCTGGTTTATTGCTGATAAATCTGGAGCCGGTGAGCGTGGGTCTCGCGGTA
TCATTGCAGCACTGGGGCCAGATGGTAAGCCCTCCCGTATCGTAGTTATCTACACGACGGGGAGTCAGGCAACTATGGATGAACGAAATAGA
CAGATCGCTGAGATAGGTGCCTCACTGATTAAGCATTGGTAACTGTCAGACCAAGTTTACTCATATATACTTTAGATTGATTTAAAACTTCATTTT
TAATTTAAAAGGATCTAGGTGAAGATCCTTTTTGATAATCTCATGACCAAAATCCCTTAACGTGAGTTTTCGTTCCACTGAGCGTCAGACCCCGT
AGAAAAGATCAAAGGATCTTCTTGAGATCCTTTTTTTCTGCGCGTAATCTGCTGCTTGCAAACAAAAAAACCACCGCTACCAGCGGTGGTTTG
TTTGCCGGATCAAGAGCTACCAACTCTTTTTCCGAAGGTAACTGGCTTCAGCAGAGCGCAGATACCAAATACTGTCCTTCTAGTGTAGCCGTA
GTTAGGCCACCACTTCAAGAACTCTGTAGCACCGCCTACATACCTCGCTCTGCTAATCCTGTTACCAGTGGCTGCTGCCAGTGGCGATAAGTC
GTGTCTTACCGGGTTGGACTCAAGACGATAGTTACCGGATAAGGCGCAGCGGTCGGGCTGAACGGGGGGTTCGTGCACACAGCCCAGCTT
GGAGCGAACGACCTACACCGAACTGAGATACCTACAGCGTGAGCTATGAGAAAGCGCCACGCTTCCCGAAGGGAGAAAGGCGGACAGGTAT
CCGGTAAGCGGCAGGGTCGGAACAGGAGAGCGCACGAGGGAGCTTCCAGGGGGAAACGCCTGGTATCTTTATAGTCCTGTCGGGTTTCGC
CACCTCTGACTTGAGCGTCGATTTTTGTGATGCTCGTCAGGGGGGCGGAGCCTATGGAAAAACGCCAGCAACGCGGCCTTTTTACGGTTCC
TGGCCTTTTGCTGGCCTTGAAGCTGTCCCTGATGGTCGTCATCTACCTGCCTGGACAGCATGGCCTGCAACGCGGGCATCCCGATGCCGCC
GGAAGCGAGAAGAATCATAATGGGGAAGGCCATCCAGCCTCGCGTCGAATTCGTCGACCTCGAAATTCTACCGGGTAGGGGAGGCGCTTTT
CCCAAGGCAGTCTGGAGCATGCGCTTTAGCAGCCCCGCTGGGCACTTGGCGCTACACAAGTGGCCTCTGGCCTCGCACACATTCCACATCC
ACCGGTAGGCGCCAACCGGCTCCGTTCTTTGGTGGCCCCTTCGCGCCACCTTCTACTCCTCCCCTAGTCAGGAAGTTCCCCCCCGCCCCG
CAGCTCGCGTCGTGCAGGACGTGACAAATGGAAGTAGCACGTCTCACTAGTCTCGTGCAGATGGACAGCACCGCTGAGCAATGGAAGCGG
GTAGGCCTTTGGGGCAGCGGCCAATAGCAGCTTTGCTCCTTCGCTTTCTGGGCTCAGAGGCTGGGAAGGGGTGGGTCCGGGGGCGGGCT
CAGGGGCGGGCTCAGGGGCGGGGCGGGCGCCCGAAGGTCCTCCGGAGGCCCGGCATTCTGCACGCTTCAAAAGCGCACGTCTGCCGCG
CTGTTCTCCTCTTCCTCATCTCCGGGCCTTTCGACCTGCATCCATCTAGATCTCGAGCAGCTGAAGCTTACCATGACCGAGTACAAGCCCACG
GTGCGCCTCGCCACCCGCGACGACGTCCCCAGGGCCGTACGCACCCTCGCCGCCGCGTTCGCCGACTACCCCGCCACGCGCCACACCGT
CGATCCGGACCGCCACATCGAGCGGGTCACCGAGCTGCAAGAACTCTTCCTCACGCGCGTCGGGCTCGACATCGGCAAGGTGTGGGTCG
CGGACGACGGCGCCGCGGTGGCGGTCTGGACCACGCCGGAGAGCGTCGAAGCGGGGGCGGTGTTCGCCGAGATCGGCCCGCGCATGG
CCGAGTTGAGCGGTTCCCGGCTGGCCGCGCAGCAACAGATGGAAGGCCTCCTGGCGCCGCACCGGCCCAAGGAGCCCGCGTGGTTCCT
GGCCACCGTCGGCGTCTCGCCCGACCACCAGGGCAAGGGTCTGGGCAGCGCCGTCGTGCTCCCCGGAGTGGAGGCGGCCGAGCGCGC
CGGGGTGCCCGCCTTCCTGGAGACCTCCGCGCCCCGCAACCTCCCCTTCTACGAGCGGCTCGGCTTCACCGTCACCGCCGACGTCGAGG
TGCCCGAAGGACCGCGCACCTGGTGCATGACCCGCAAGCCCGGTGCCTGACGCCCGCCCCACGACCCGCAGCGCCCGACCGAAAGGAG
CGCACGACCCCATGCATCGATGATATCAGATCCCCGGGATGCAGAAATTGATGATCTATTAAACAATAAAGATGTCCACTAAAATGGAAGTTTTT

CCTGTCATACTTTGTTAAGAAGGGTGAGAACAGAGTACCTACATTTTGAATGGAAGGATTGGAGCTACGGGGGTGGGGGTGGGGTGGGATTA
GATAAATGCCTGCTCTTTACTGAAGGCTCTTTACTATTGCTTTATGATAATGTTTCATAGTTGGATATCATAATTTAAACAAGCAAAACCAAATTAA
GGGCCAGCTCATTCCTCCCACTCATGATCTATAGATCTATAGATCTCTCGTGGGATCATTGTTTTTCTCTTGATTCCCACTTTGTGGTTCTAAGT
ACTGTGGTTTCCAAATGTGTCAGTTTCATAGCCTGAAGAACGAGATCAGCAGCCTCTGTTCCACATACACTTCATTCTCAGTATTGTTTTGCCA
AGTTCTAATTCCATCAGAAGCTGGTCGACATTGGATTGGAAGTAGTATACTAACCTTCACCTATTGCAATTCTCATGTTTGACAGCTTATCATCG
CAGATCCGGGCAACGTTGTTGCCATTGCTGCAGGCGCAGAACTGGTAGGTATGGAAGATCTATACATTGAATCAATATTGGCAATTAGCCATAT
TAGTCATTGGTTATATAGCATAAATCAATATTGGCTATTGGCCATTGCATACGTTGTATCTATATCATAATATGTACATTTATATTGGCTCATGTCCAA
TATGACCGCCAT

13.2 pCEP4 L1RP-ENm (H230A) (L1RP-ENm) and pCEP4 L1RP-RTm (D702Y) (L1RP-RTm) plasmids share the same sequences as pCEP4 L1RP except for the following minor key changes:

	EN (3362-3364 bp)	RT (4778-4780bp)
**pCEP4 L1RP**	H230 (CAC)	D702 (GAC)
**pCEP4 L1RP-ENm (H230A)**	A230 (GCC)	D702 (GAC)
**pCEP4 L1RP-RTm (D702Y)**	H230 (CAC)	Y702 (TAT)
