# Supplementary material for: Frequency and mechanisms of LINE-1 retrotransposon insertions at CRISPR/Cas9 sites
Source: Nat Commun. 2022 Jun 27;13:3685. doi: 10.1038/s41467-022-31322-3 (PMC9237045; doi:10.1038/s41467-022-31322-3)
Supplement: Supplementary file 2 — Reporting Summary [file 41467_2022_31322_MOESM2_ESM.pdf]

nature portfolio

Corresponding author(s): Roberto Chiarle

Last updated by author(s): 5/26/2022

Reporting Summary

Nature Portfolio wishes to improve the reproducibility of the work that we publish. This form provides structure for consistency and transparency in reporting. For further information on Nature Portfolio policies, see our Editorial Policies and the Editorial Policy Checklist.

Statistics

For all statistical analyses, confirm that the following items are present in the figure legend, table legend, main text, or Methods section.

n/a	Confirmed	
☐	☒	The exact sample size (*n*) for each experimental group/condition, given as a discrete number and unit of measurement
☐	☒	A statement on whether measurements were taken from distinct samples or whether the same sample was measured repeatedly
☐	☒	The statistical test(s) used AND whether they are one- or two-sided<br>*Only common tests should be described solely by name; describe more complex techniques in the Methods section.*
☒	☐	A description of all covariates tested
☒	☐	A description of any assumptions or corrections, such as tests of normality and adjustment for multiple comparisons
☐	☒	A full description of the statistical parameters including central tendency (e.g. means) or other basic estimates (e.g. regression coefficient) AND variation (e.g. standard deviation) or associated estimates of uncertainty (e.g. confidence intervals)
☐	☒	For null hypothesis testing, the test statistic (e.g. *F*, *t*, *r*) with confidence intervals, effect sizes, degrees of freedom and *P* value noted<br>*Give P values as exact values whenever suitable.*
☒	☐	For Bayesian analysis, information on the choice of priors and Markov chain Monte Carlo settings
☒	☐	For hierarchical and complex designs, identification of the appropriate level for tests and full reporting of outcomes
☒	☐	Estimates of effect sizes (e.g. Cohen's *d*, Pearson's *r*), indicating how they were calculated

Our web collection on statistics for biologists contains articles on many of the points above.

Software and code

Policy information about availability of computer code

Data collection	For western blotting, images were collected with Image Lab, and the relative expression of the ORF1p protein was measured by densitometry using Image J.
Data analysis	Open sources BOWTIE2 v2.1, BEDTOOLS v2.29, BLAST v2.11, R 3.6.2, R package 'ggplot2', Biostrings', 'ggseqlogo' and seqLogo' as well as lab-developed tools available at https://github.com/geatools/geat were used for data process and analysis.<br>FlowJo for analyzing the FACS data.

For manuscripts utilizing custom algorithms or software that are central to the research but not yet described in published literature, software must be made available to editors and reviewers. We strongly encourage code deposition in a community repository (e.g. GitHub). See the Nature Portfolio guidelines for submitting code & software for further information.

Data

Policy information about availability of data

All manuscripts must include a data availability statement. This statement should provide the following information, where applicable:

- Accession codes, unique identifiers, or web links for publicly available datasets
- A description of any restrictions on data availability
- For clinical datasets or third party data, please ensure that the statement adheres to our policy

All sequencing data has been deposited in the Gene Expression Omnibus database under accession number GSE178440. Source Data for figures are provided with the online version of the paper.

March 2021

Field-specific reporting

Please select the one below that is the best fit for your research. If you are not sure, read the appropriate sections before making your selection.

- [x] Life sciences
- [ ] Behavioural & social sciences
- [ ] Ecological, evolutionary & environmental sciences

For a reference copy of the document with all sections, see nature.com/documents/nr-reporting-summary-flat.pdf

Life sciences study design

All studies must disclose on these points even when the disclosure is negative.

Sample size	Sample size selection was based on the lab's previous experience to ensure reproducibility. The sample size for each group is detailed in the figure legends and Methods. All experiments were performed using independent biological (2-6) replicates.
Data exclusions	We didn't exclude data
Replication	All experiments were performed using independent biological (2-6) replicates. All attempts at replication were successful.
Randomization	It is not relevant to our study as it didn't involve mouse studies, patients or clinical trials
Blinding	It is not relevant to our study as it didn't involve mouse studies, patients or clinical trials

Reporting for specific materials, systems and methods

We require information from authors about some types of materials, experimental systems and methods used in many studies. Here, indicate whether each material, system or method listed is relevant to your study. If you are not sure if a list item applies to your research, read the appropriate section before selecting a response.

Materials & experimental systems

n/a	Involved in the study	
☐	☒	Antibodies
☐	☒	Eukaryotic cell lines
☒	☐	Palaeontology and archaeology
☒	☐	Animals and other organisms
☒	☐	Human research participants
☒	☐	Clinical data
☒	☐	Dual use research of concern

Methods

n/a	Involved in the study	
☒	☐	ChIP-seq
☐	☒	Flow cytometry
☒	☐	MRI-based neuroimaging

Antibodies

Antibodies used	Antibody against ORF1p and b-actin used for Western Blotting
Validation	ORF1p has been validated: Ardeljan, D. et al. Cell fitness screens reveal a conflict between LINE-1 retrotransposition and DNA replication. Nat Struct Mol Biol 27, 168-178 (2020).<br>B-actin is commercially available (Sigma: A5316).

Eukaryotic cell lines

Policy information about cell lines

Cell line source(s)	HEK293T cells (ATCC), HeLa cells (ATCC); The U2OS cells was provided by Jeremy Stark, and validated by STR profiling (PMID: 31753920)
Authentication	HEK293T and HeLa were authenticated as they were purchased from ATCC; U2OS cells were validated by STR profiling (PMID: 31753920)
Mycoplasma contamination	All cell lines tested negative for mycoplasma contamination
Commonly misidentified lines (See ICLAC register)	No commonly misidentified cell lines were used in the study

Flow Cytometry

Plots

Confirm that:

- [x] The axis labels state the marker and fluorochrome used (e.g. CD4-FITC).
- [x] The axis scales are clearly visible. Include numbers along axes only for bottom left plot of group (a 'group' is an analysis of identical markers).
- [x] All plots are contour plots with outliers or pseudocolor plots.
- [x] A numerical value for number of cells or percentage (with statistics) is provided.

Methodology

Sample preparation	The dissociated cells were washed by PBS and directly analyzed by FACS without staining
Instrument	FACSVerse flow cytometer (BD biosciences)
Software	BD FACSDiva Software for collecting the data and FlowJo for analyzing the data
Cell population abundance	we didn't sort the cells
Gating strategy	We used FLowJo to analyze the data. Live cells were gated from the FSC/SSC gate for further analysis. Then, we gated the cells positive on the 488nm channel to show the percentage of GFP+ cell populations (see supplementary Fig. 1c)

- [x] Tick this box to confirm that a figure exemplifying the gating strategy is provided in the Supplementary Information.
