# Supplementary material for: Frequency and mechanisms of LINE-1 retrotransposon insertions at CRISPR/Cas9 sites
Source: Nat Commun. 2022 Jun 27;13:3685. doi: 10.1038/s41467-022-31322-3 (PMC9237045; doi:10.1038/s41467-022-31322-3)
Supplement: Supplementary file 4 — Description of Additional Supplementary Files [file 41467_2022_31322_MOESM4_ESM.pdf]

Title: Supplementary Data 1:
Description: Information of Amplicon sequencing libraries

Title: Supplementary Data 2:
Description: Information of PolyA-seq libraries. We are uploading them as separate files.
